# Exosomes Play an Important Role in the Progression of Plasma Cell Mastitis via the PI3K-Akt-mTOR Signaling Pathway

**DOI:** 10.1155/2019/4312016

**Published:** 2019-06-09

**Authors:** Xiaohong Wang, Yong Han, Jian Liu, Yingzhe Zhang, Kai Cheng, Jiwei Guo, Qingqun Guo, Song Liu, Hongguang Sun, Yitong Hua, Guoqiang Zhang, Shujian Xu, Fengli Guo, Zhenlin Yang

**Affiliations:** ^1^Department of Thyroid and Breast Surgery, Binzhou Medical University Hospital, Binzhou, Shandong 256603, China; ^2^Cancer research institute, Binzhou Medical University Hospital, Binzhou, Shandong 256603, China

## Abstract

**Background:**

Plasma cell mastitis (PCM) is one of the most frequently encountered inflammatory diseases of the nonlactating breast. However, its pathogenesis has remained unknown.

**Methods:**

In this study, we observed the ultrastructure changes of PCM by a transmission electron microscope. The transcriptome expression difference of exosomes was detected by RNA-Seq; then, we confirmed the key difference genes by western blot and immunohistochemistry. Finally, we established the mouse PCM model by tissue homogenate injection to validate the role of exosomes on the progression of PCM.

**Results:**

The analysis of the exosomal transcriptome expression difference between PCM and normal mammary tissues using RNA-Seq showed the differential genes and enrichment pathways involved in the course of PCM. The decreased HSP90AA1 and EEF2, excessive production of p-AKT, and p-mTOR were consistent with clinical specimens. Inhibition of exosome secretion significantly inhibited inflammatory cell infiltration, and the mammary duct had maintained a better structure in the PCM mouse model.

**Conclusion:**

Our results revealed the role of exosomes acting as critical signal introduction facilitators in the progression of plasma cell mastitis and identified potential key genes in the regulation of this process. These results will help to dissect the molecular mechanism of PCM and provide therapeutic targets.

## 1. Introduction

Plasma cell mastitis (PCM) is an inflammatory disease of the breast parenchyma, characterized by a periductal phlogistic reaction, accompanied by ductal ectasia. The incidence of PCM is approximately 5% of breast cancer and has increased gradually in recent years [1]. PCM usually affects women of childbearing age; they were also reported in patients as young as 11 years old [2, 3] and as old as 80 years [4, 5]. The management of PCM remains to be a particular problem as lacking of molecular pathogenesis. But so far, treatment options for PCM are limited and surgery is still the most radical and effective treatment. However, the operation could not prevent the recurrence of the disease as a discrete mass even though the mammary gland was removed. The recurrence rate reached up to 79% if the lactiferous ducts are not excised and decreased 28% after the excision of the lactiferous ducts. Women might experience repeated incision which can lead to breast deformation and cause physical and psychological distress. Chinese medicine treatment has also been recommended but with visible individual diversities. Recently, albeit combined therapy of fiberoptic ductoscopy and traditional Chinese medicine has been reported and brought good curative effect [6], but the long-term efficacy needs further evaluation. No consensus currently exists as to the ideal treatment regimen, and recurrence rates remain as high as 50% [7]. Therefore, it is critical to investigate the mechanism underlying the pathological progression of PCM.

PCM is histopathologically defined as a chronic inflammation of the breast, with dilation of the mammary duct, plasma cell infiltration, and abscess formation [8]. Inflammatory response is a well-regulated process of an integrated and complex network of cellular communication. Recent evidence has shed light on a novel mode of intercellular communication mediated by exosomes in regulating inflammation [9, 10] and autoimmune diseases, such as arthritis [11, 12] and diabetes [13]. Exosomes from both immune and nonimmune cells, such as endothelial cells, contribute to antigen-specific and nonspecific immune regulation. Given their ability to modulate immune responses, exosomes have tremendous potential as therapeutic agents for treating a variety of human diseases and disorders, including reducing inflammation, treating autoimmune diseases, and stimulating antipathogen immune responses.

At present, most of current research on the PCM is about clinical features and treatment of the disease. Due to the lack of cell lines and animal models, the mechanism of PCM and pathological change were still unknown. In this study, the ultrastructure changes of PCM were observed by a transmission electron microscope. We analyzed the transcriptome expression difference of exosomes extracted from normal and PCM tissue by RNA-Seq; then, we confirmed the key difference genes by western blot analysis and immunohistochemistry. Finally, we established the mouse PCM model by tissue homogenate injection to investigate the role of exosomes on the progression of PCM. Our study will explore the pathogenesis of PCM from the perspective of exosomes and provide therapeutic targets for the clinical treatment of PCM.

## 2. Methods

### 2.1. Risk Factor Analysis

A retrospective chart review was performed on patients diagnosed with PCM at Binzhou Medical University Hospital between 1 January 2011 and 31 December 2017. The following data from each patient's chart were reviewed: age, body mass index (BMI), nipple or breast dysplasia, autoimmune diseases,active smoking, bacterial infection, mammary duct dilatation, mammary duct injury, taking psychotropic drugs, and menstrual disorder.

### 2.2. Transmission Electron Microscopy Processing and Observation

The PCM tissues and control tissues which were taken from normal tissues next to PCM were immobilized in 2.5% glutaraldehyde at 4°C for up to 36 hours and further processed by postfixation in osmium tetroxide, en bloc staining with uranyl acetate, dehydration in ethanol, and embedding in epoxy resin. Thin sections were poststained with lead citrate and examined using an HT7700 transmission electron microscope (HITACHI, Japan). Then, 5 different fields under the microscope were randomly chosen for quantification at an acceleration voltage of 200 kV by 2 pathologists in a blinded manner.

### 2.3. Extraction of Tissue Exosomes

The PCM tissues and normal control tissues next to PCM were separately homogenized at medium speed on ice with physiological saline at a ratio of 1 : 3. The tissue homogenate was filtered through a 40 *μ*m mesh filter and then a 0.2 *μ*m filter. Then, the filtrate was centrifuged to remove debris at 3000g for 10 min. Next, the supernatant was centrifuged at 10000g for 20 min to remove impurities. Then, the RIBO™ Exosome Isolation Reagent (RIBO, Guangzhou, China) (REI) was added following the reagent's instructions at 4°C overnight. Finally, the mixture was centrifuged at 1500g for 30 min to discard the supernatant and obtain exosomes for a follow-up test. The exosomes extracted from the PCM and the normal mammary tissues were named PCM/exo and N/exo, respectively. Exosomes were analyzed by transmission electron microscopy and ZetaView® NTA technique by Particle Metrix (Malvern, Worcestershire, UK) for identification and characterization.

### 2.4. RNA-Seq and Bioinformatic Analysis

Total RNA were respectively extracted from PCM/exo and N/exo according to the instructions of NEBNext® Poly(A) mRNA Magnetic Isolation Module (San Diego, CA, USA). The RNA-Seq and bioinformatic analysis, such as gene ontology (GO) enrichment analysis, and the Kyoto Encyclopedia of Genes and Genomes (KEGG) pathways analysis were performed as described before [14]. The differentially expressed genes (DEGs) between PCM/exo and N/exo were selected by the difference multiples (log_2_FoldChange|_>1_) and significant levels (*q* value < 0.001). The overall distribution of DEGs is counted by a volcano plot. The *p* value was calculated by the Fisher exact test, *p* < 0.05 was the significance threshold, and the distribution information and significance of the gene set in the KEGG category were obtained. According to genetic GO annotation, using a hypergeometric distribution method to calculate the *p* value and *p* < 0.05 for significant threshold having a statistical significance relative to the background of high frequency annotations, we get the distribution of genes in the category information and significance. The protein-protein interaction (PPI) network was constructed using the STRING online database.

### 2.5. Immunohistochemistry and Western Blot Analysis

Based on the above results, we detected the expression of mTOR, HSP90AA1, and EEF2 in 20 PCM tissues. 20 specimens of normal adjacent tissues randomized from our breast cancer pathology specimen bank served as controls. We further measure the activation of the PI3K-Akt-mTOR signaling pathway in PCM tissue sections. Tissue sections were subjected to routine toasting, dehydration, endogenous peroxidase inactivation, and antigen retrieval. Samples were incubated with primarily indicated antibodies diluted at 1 : 100 overnight at 4°C: PI3K p85 alpha (Proteintech, 60225-1-Ig), p-AKT (Tr308) and AKT (pan) (Cell Signaling Technology), phospho-mTOR (Abcam, ab109268), HSP90AA1 (Boster, BA0369), and EEF2 (Boster, BM1733), and secondary rabbit-anti-human Histostain™-SP Kit (SPN 9001 ZSGB-BIO) for 1 h at room temperature. Diaminobenzidine (DAB) substrate was used for sample staining. Three different fields under the microscope (×200) for each immunohistochemical slides were randomly chosen for scoring. Positive proportion and intensity were semiquantitatively scored by 2 pathologists in a blinded manner. Total protein was extracted from tissues using RIPA, and protein concentrations were measured using a BCA assay Kit (Beyotime Biotechnology); then, 25 *μ*g protein was loaded. The following antibodies were used: *β*-actin (1 : 2500), PI3K p85 alpha (1 : 1000), p-AKT (Tr308) and AKT (pan) (1 : 2000), and phospho-mTOR (1 : 2000). Secondary antibody was applied for 1 h at 37°C on the next day. An enhanced chemiluminescence (ECL) plus kit (Millipore, America) was applied for visualization. ImageJ software was used for the densitometric analysis of the bands, and all values were normalized to *β*-actin.

### 2.6. Murine Model of PCM Animal

Sexually mature female BALB/c mice aged 8 weeks purchased from Beijing Vitalriver Co., Ltd and were kept under a 12 h light/dark cycle at the Animal Care Facility and acclimatized for at least 5 days prior to experiments. We established the mouse PCM model by in situ injection of tissue homogenate. The RNA-Seq data suggested that exosomes may have played an important role during the progression of PCM. Therefore, we utilized GW4869 to block exosome-mediated gene transmission in the PCM mouse model to validate our assumption.

PCM tissues were acquired during surgery from patients, and 0.1g of tissue was homogenized for 5 minutes use the electrically driven tissue homogenizer (Tiangen Biotech Co. LTD., OSEY30) on ice with 3 volume physiological saline, then filtered through a 200-mesh strainer. We selected the 3rd and 4th pairs of lacteal glands of BALB/c mice as the injection sites. The mice were randomly divided into 4 groups with 6 mice in each group. Mice in groups A and B as control groups were injected with 0.02 ml physiological saline and 0.02 ml complete Freund's adjuvant (CFA) (Sigma-Aldrich Co. Ltd., USA), respectively. Mice in group C were injected with a 0.02 ml mixture of PCM tissue homogenate with CFA at the ratio of 1 : 1 [15]. Mice in group D were injected with 0.02 ml mixture for modeling, then locally injected in the inflammatory region with 1 *μ*g GW4869 every day [16] after 5 days of modeling. Animals were sacrificed 2 weeks after inoculation. Pathological changes were detected by HE staining. Positive results were designated for animals with PCM pathological changes, otherwise negative. All methods were approved by the Institutional Animal Care and Use Committee of Binzhou Medical University Hospital (No. 2018-020-01). All experiments were conducted in accordance with the guidelines of the Ministry of Health of PR China and the Animal Care Committee of Binzhou Medical University. Positive results were designated for animals with PCM pathological changes by 2 pathologists, otherwise negative. The degree of inflammatory cell infiltration was performed 3 times using serial sections from the same paraffin-embedded tissues, and 3 different fields under the microscope (×200) for each slides were randomly chosen for semiquantitative scoring by 2 pathologists in a blinded manner. Then, the obtained scores were expressed as means ± SD and analyzed by one-way ANOVA.

### 2.7. Statistical Analysis

All experiments were performed in triplicate, and representative data were shown from three separate experiments. A statistical analysis was performed using a *t*-test or one-way ANOVA using the SPSS 23.0 statistical software. All experiments were performed in triplicate, and *p* < 0.05 was considered statistically significant. GraphPad was used for graph generation.

## 3. Results

### 3.1. Demographics and Risk Factors

In this study, we analyzed the distributions of age and other risk factors for PCM. All these patients are Asian (Chinese Hans). The most frequent age of onset in the studied population was 30-39 years (65.0%), following 40-49 years (20.0%), and 19-29 years (15.0%), respectively. Briefly, a higher proportion of cases was overweight (35% overweight and 25% obese) and nipple or breast dysplasia (45%). The frequency of mammary duct dilatation and mammary duct injury was 25.0% and 5.0%, respectively. Among these samples, 1 (5.0%) is active smoking, 1 (5.0%) is taking psychotropic drug, and 2 (10.0%) had menstrual disorder. No patient had a history of autoimmune diseases, and no patient had positive bacterial culture (Table 1).

### 3.2. Transmission Electron Microscopy Processing and Observation

**Figure 1:**
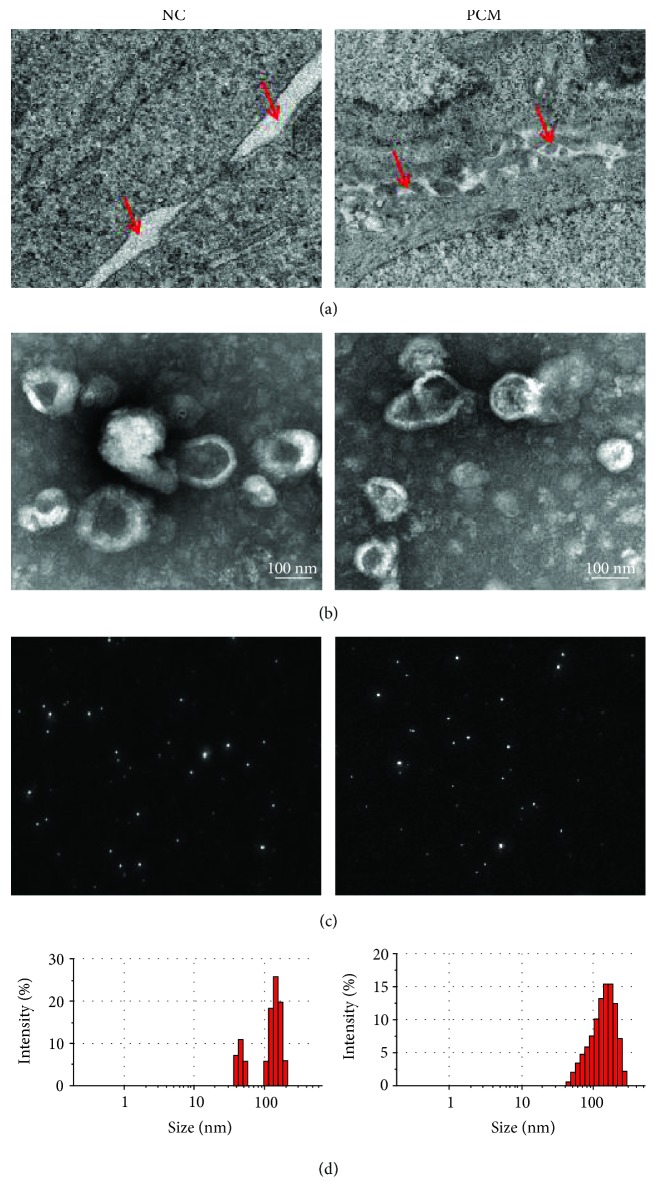
The ultrastructure changes of PCM and exosomes extracted from normal and PCM tissues were observed by a transmission electron microscope. (a) More microvesicles were found in the synaptic cleft of PCM indicated with red arrows. (b) A representative transmission electron microscopy image of PCM/exo and N/exo, showing a typical “saucer-like” morphology (scale bar, 100 nm). (c, d) Analysis of exosome size indicated similar particle size distribution of PCM/exo and N/exo.

The microvilli of normal breast epithelial cells were arranged neatly and developed well. The intercellular gap junctions, inlay connections, and desmosomes were also well developed. However, the ductal epithelial basement membrane of plasma cell mastitis was destroyed, and the gap junction and mosaic connection were reduced. There were more free ribosomes, microfilaments, lipid droplets, and cytoplasmic lumen between the two contiguous membranes suggesting a juxtacrine cell-to-cell signaling (chemical synapse), via juxtacrine, a specific case of phenomena. We proposed that exosomes might represent epithelial cells of the acini and ducts participate in juxtacrine/paracrine signaling and play a role in the progression of PCM (Figure 1(a)).

### 3.3. Identification and Characterization of Exosomes

Transmission electron microscopy analysis showed that the exosomes isolated from PCM and the normal breast tissues were morphologically homogeneous, ranging from 30 to 150 nm in size, with a typical round or cup shape appearance (Figure 1(b)). The particle size distribution of nano-AE PBS aqueous solution was detected using Zeta View S/N 252. About 77.9% N/exo displayed a size ranging from 39.41 to 147.71 nm and 74.12% PCM/exo ranging from 45.64 to 153.23 nm, and there was no statistically significant difference (Figures 1(c) and 1(d)).

### 3.4. Functional Annotation and Classification of the Reference Transcriptome

**Figure 2:**
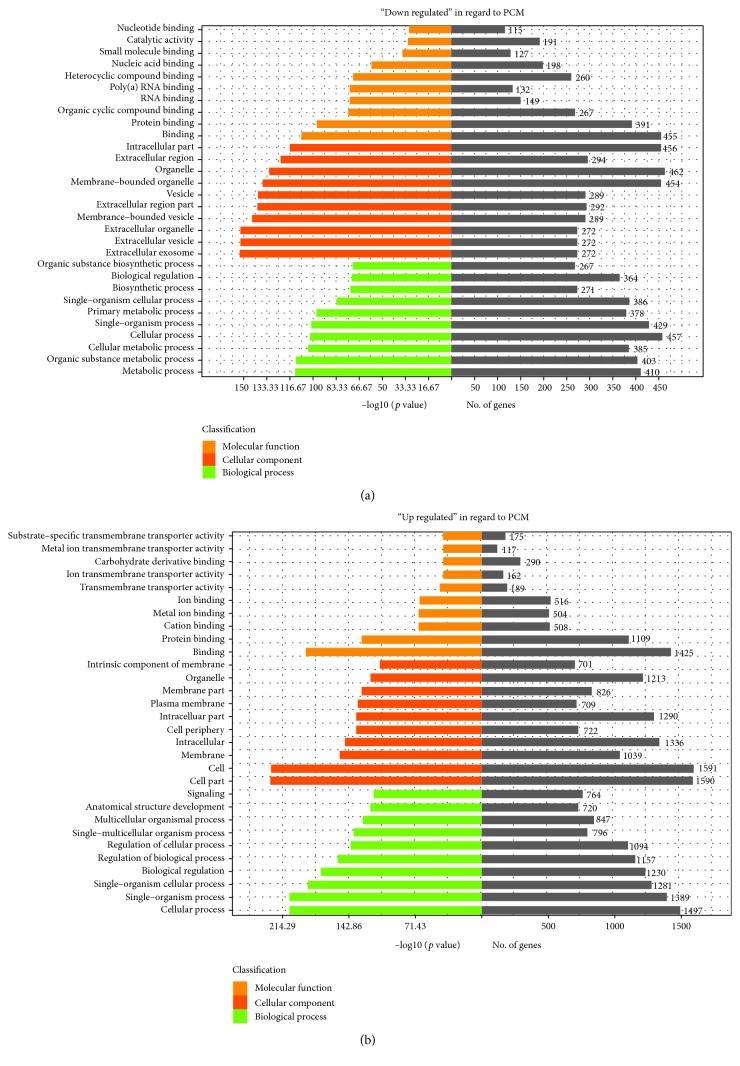
The top 30 most significant changes in the GO biological process. (a) The downregulated DEGs were enriched in binding, the metabolic process, and the biosynthetic process. (b) The upregulated DEGs were enriched in the biological process as a cellular process, regulation of biological process, and regulation of cellular process.

A total of 2978 DEGs (differential expression genes) including 2397 upregulated genes and 581 downregulated genes were screened. To explore potential function and pathways for these DEGs, GO functional enrichment of both upregulated and downregulated DEGs was analyzed. In the three main GO categories (cellular component, biological process, and molecular function), the downregulated DEGs were enriched in binding, metabolic process, and biosynthetic process (Figure 2(a)) and upregulated DEGs were enriched in the biological process as cellular process, regulation of biological process, and regulation of cellular process (Figure 2(b)).

**Figure 3:**
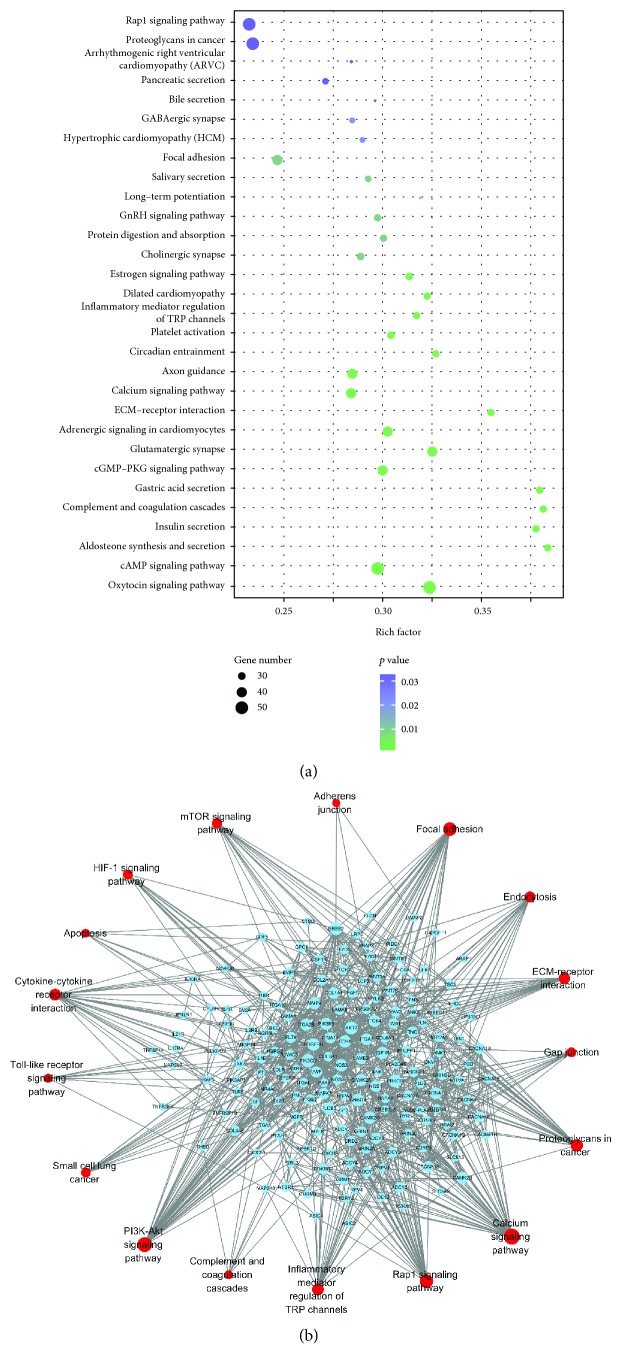
The KEGG pathway analysis and the PPI network of DEGs. (a) The top 20 most significant KEGG pathway terms. (b) The PPI network of overexpressed DEGs.

Pathway-based analysis, performed by searching against the KEGG database, helps to further understand the biological functions and interactions of genes. There were 1024 unigenes mapped to 30 KEGG pathways. The main pathways were Rap1 signaling pathway, cAMP signaling pathway, calcium signaling pathway, oxytocin signaling pathway, cGMP-PKG signaling pathway, focal adhesion, axon guidance, gastric secretion, complement and coagulation cascades, insulin secretion, and aldosterone secretion pathway (Figure 3(a)).

### 3.5. Protein-Protein Interaction (PPI) Network Analysis of the Different mRNA Expression

To determine the interaction relationship between the proteins expressed by these upregulated DEGs, a PPI network was constructed as demonstrated in Figure 3(b), such as the mTOR signaling pathway, PI3K-Akt signaling pathway, adherens junction, focal adhesion, gap junction, endocytosis, ECM-receptor, proteoglycans in cancer, inflammatory mediator regulation of TRP channels, calcium signaling pathway, Rap1 signaling, complement and coagulation cascades, cytokine-cytokine receptor interaction, apoptosis, and Toll-like receptor signaling pathway. These annotations would be a valuable resource for further research on specific processes, functions, and pathways of PCM.

### 3.6. AKT-mTOR Signaling Pathway Activation Was Detected in PCM, and Inhibiting Exosome Secretion Reduced Inflammation In Vivo

**Figure 4:**
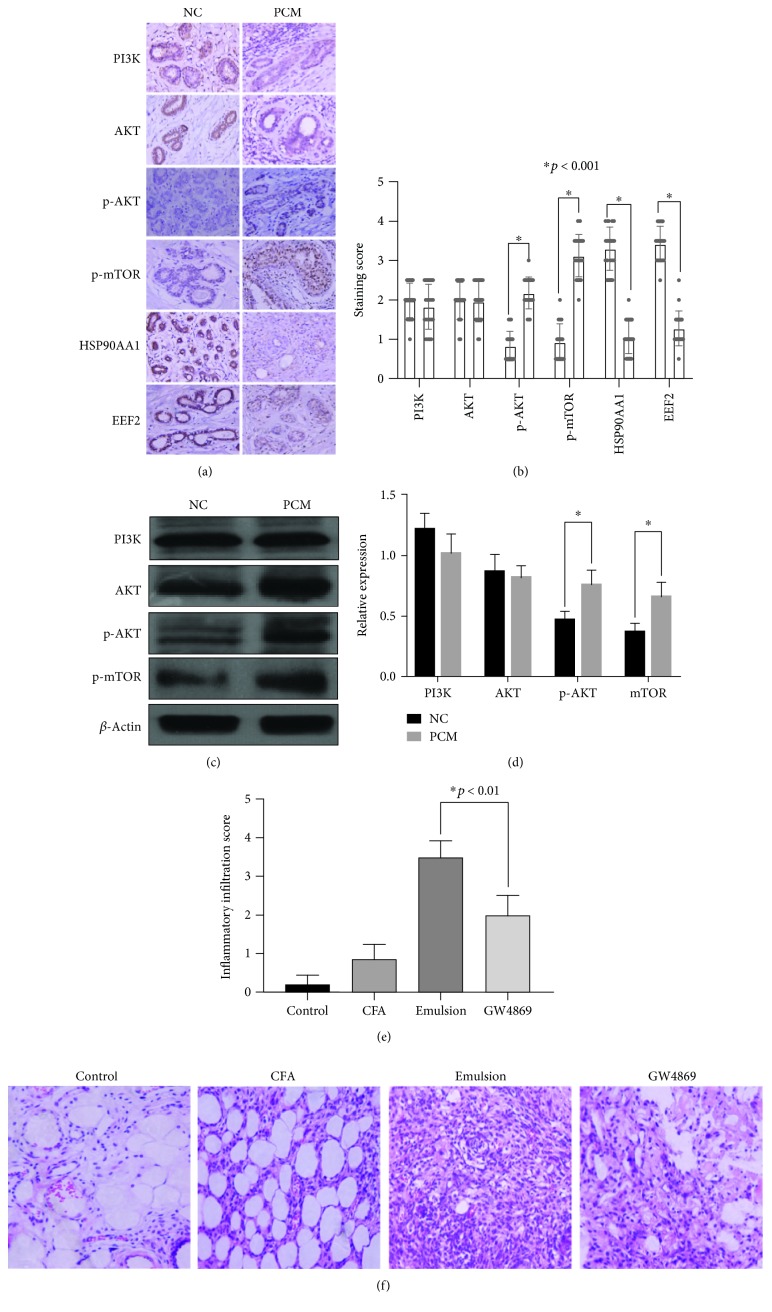
AKT-mTOR signaling pathway activation was detected in PCM, and inhibiting exosome secretion reduced inflammation in vivo. (a) The expression of PIK3K, AKT, p-AKT, p-mTOR, HSP90AA1, and EEF2 was detected in PCM tissue sections by immunohistochemical staining. (b) Immunohistochemical staining scores were analyzed by the Mann-Whitney *U* test. There were no statistical differences for PIK3K and AKT in PCM tissues compared with NC tissues, while p-AKT and p-mTOR were overexpressed and HSP90AA1 and EEF2 were downexpressed in PCM tissues. ^∗^ *p* < 0.001 versus NC groups. (c) Western blots analyze changes of PI3K-Akt-mTOR pathway-related proteins. (d) Densitometric quantification for the changes of PI3K-Akt-mTOR pathway-related proteins was measured by QUANTITY ONE software. All values are expressed as the mean ± SD. ^∗^ *p* < 0.05 versus NC groups. (e, f) Pathological changes were detected by HE staining and inhibition of exosomes secretion by GW4869 significantly inhibited inflammatory cell infiltrate. Inflammatory infiltration scores were analyzed by the Mann-Whitney *U* test. ^∗^ *p* < 0.01.

Based on the above results, we detected the expression of mTOR, HSP90AA1, and EEF2 in PCM tissue sections. We found that HSP90AA1 and EEF2 were both downregulated in PCM which were consistent with the RNA-Seq results for PCM/exo and N/exo (Figure 4(a)). Furthermore, immunohistochemistry staining and western blotting were performed to detect activated p-AKT and p-mTOR in PCM. PCM tissues had higher level of p-AKT and p-mTOR compared to normal tissues (control) as shown in Figures 4(a) and 4(b) was the quantification of p-AKT, total AKT, and p-mTOR. There was a significant increase of p-AKT and p-mTOR in PCM compared with the control group (Figures 4(c) and 4(d)). This suggests that the activated AKT-mTOR signaling pathway may play an important role in PCM, and this process may be mediated by exosomes.

We hypothesized that the reduction of exosome secretion may suppress plasma cell mastitis. GW4869, an inhibitor of neutral sphingomyelinase (nSMase), has been reported to significantly decrease the release of exosomes from cells [17]. To test our hypothesis, we used GW4869 in the PCM model. It is evident that mice in the emulsion group (CFA+homogenate) exhibiting histological features of PCM had a significant infiltration of many plasma cells and some lymphocytes around the mammary ducts, while the histological analysis of the mammary gland of mice in the GW4869-treated group found that fewer infiltration of plasma cells and lymphocytes around the mammary ducts and the mammary duct had maintained a better structure (Figure 4(f)). Our results suggest that inhibition of exosome secretion significantly inhibited inflammatory cell infiltration (Figure 4(e)). Also, manifestation of PCM was not observed in only physiological saline group and CFA control group which was only slight infiltration of neutrophils and lymphocytes were observed.

## 4. Discussion

PCM is a refractory mastitis, the therapeutic options are very limited, and its etiology and pathogenesis remain unclear. Some studies have suggested that this disease is closely related to smoking. However, there are 20 patients in this study, and only 1 patient had a smoking history. 9 patients (45%) were accompanied with nipple retraction or breast dysplasia, and 12 patients (60%) were overweight which may be both the important factors for PCM. The goal of this study was to understand the roles of exosomes in the development of PCM. Our results suggest that increased exosome secretion of mRNAs, which can regulate several inflammatory pathways, may play an active role in the pathogenesis of PCM. To study this possibility, we analyzed the transcriptome expression difference of exosomes extracted from normal and PCM tissue by RNA-Seq, then confirmed the key difference genes in tissue specimens, and investigated the effects of inhibiting exosome release on the PCM model in mice.

In our study, we conducted a functional enrichment analysis of the overexpressed DEGs mainly enriched in the cytomembrane, such as the biological process, cellular process, regulation of biological process, and regulation of cellular process, and the downregulated DEGs were enriched in binding, metabolic process, and biosynthetic process. Through PPI analyses, we found that 3 mRNAs (mTOR, HSP90AA1, and EEF2) had higher degrees and protein-protein pairs. This expression was significantly different between PCM and normal breast tissues. mTOR belongs to a family of phosphatidylinositol kinase-related kinases. There is a growing appreciation of mTOR in adaptive immunity for its crucial roles in keeping a proper balance between T cell quiescence and activation [18]. Jones et al. also found that mTOR signaling plays critical but diverse roles in the early and late phases of antibody responses and plasma cell differentiation [19]. mTOR activity is required to maintain the canonical endocytic recycling pathway against lysosomal delivery which may be related to the secretion of exosomes and the separation of contents [20]. HSP90AA1 is a protein-coding gene which is involved in maintaining the homeostasis of cells. When cells are subjected to external stimulation such as heat, hypoxia, and radiation, they can be secreted extracellularly to participate in tissue repair. After the wound was treated by HSP90AA1 in deep second degree scald mice, inflammation was reduced, granulation tissue showed significant development, and the epidermal cells at the wound margins progressed more rapidly [21]. Consequently, HSP90AA1 may be a promising candidate for PCM treatment. EEF2 is an essential factor for protein synthesis and decreased expression of EEF2 can inhibit the elongation of the polypeptide chain or increase the specific protein synthetic, then reduce the consumption of nutrients and energy by cells to help cells spend an unfavorable environment [22]. The phosphorylation phenotype of EEF2 could promote nuclear dysfunction and promote cell apoptosis. The lysed EEF2 fragment can also undergo nuclear translocation, resulting in the loss of nuclear stability and dysfunction [23]. Decreased expression of EEF2 may play an important role in the development of PCM, and some regulatory mechanisms for this process are likely to become new targets.

The activation of Akt and mTOR is closely related to inflammation, and the inhibition of their phosphorylation inhibits nuclear translocation of NF-*κ*B. Subsequently, the production of inflammatory factors such as IFN-*γ*, TNF-*α*, and IL-1*β* are also inhibited [24]. Inflammation that occurs in LPS-induced keratinocytes can also be controlled by inhibition of Akt or mTOR. Inflammation induced by proinflammatory factors can be controlled by inhibiting this pathway [25]. Inhibition of PI3K/Akt/mTOR can also inhibit colonic inflammation in proinflammatory factor-induced colonic diseases and colitis [26]. In our study, we found that PCM tissues had a higher level of p-AKT and p-mTOR compared to normal tissues. This suggests that the activated AKT-mTOR signaling pathway may play an important role in PCM, and this process may be mediated by exosomes. The previous research has found that the IL-6/JAK2/STAT3 signaling pathway played a critical role in orchestrating the pathogenesis of PCM [27] which brought inspiration and support for our research. The activation of both STAT and PI3K/AKT signaling could be mediated by JAK, and inflammatory factors may upregulate the PI3K/AKT/mTOR signaling pathway by activating the JAK2/STAT3 signaling pathway [28].

## 5. Conclusions

Above all, exosomes can act as critical signal transduction facilitators representing epithelial cells of the acini and ducts via releasing a wide variety of biological molecules affecting the interaction of distant cells in the progression of plasma cell mastitis. With the development of novel therapeutic strategies targeting or utilizing exosomes, it will lead to more effective prevention and intervention strategies in PCM therapy. However, in exosome-based therapies, exosomes serving as predictive and prognostic biomarkers still need to be firmly validated by further clinical validation studies and animal testing.

## Figures and Tables

**Table 1:** Risk factor analysis of PCM.

Risk factors	Number of cases (*n* = 20)	Percentage (%)
Age (years)		
19-29	3	15.00
30-39	13	65.00
40-49	4	20.00
BMI		
<18.5 (underweight)	0	0
18.5≦BMI < 24.0 (normal)	8	40.00
24.0≦BMI < 28.0 (overweight)	7	35.00
≧28 (obese)	5	25.00
Nipple or breast dysplasia	9	45.00
Autoimmune diseases	0	0.00
Active smoking	1	5.00
Bacterial infection	0	0.00
Mammary duct dilatation	4	25.00
Mammary duct injury	1	5.00
Taking psychotropic drugs	1	5.00
Menstrual disorder	2	10.00

## Data Availability

The datasets used and/or analyzed during the current study are available from the corresponding authors on reasonable request.

## Abbreviations

PCM: Plasma cell mastitis
N/exo: Exosomes extracted from normal breast tissues
PCM/exo: Exosomes extracted from plasma cell mastitis tissues
GO pathway analysis: Gene ontology pathway analysis
KEGG pathway analysis: Kyoto Encyclopedia of Genes and Genomes pathway analysis
DEGs: Differential expression genes
PPI: Protein-protein interaction
BMI: Body mass index.

## Supplementary Materials

Supplementary Materials A total of 2978 DEGs (differential expression genes) including 581 downexpressed genes and 2397 upexpressed genes in PCM/exo.

## References

[1] Lannin D. R. (2004). Twenty-two year experience with recurring subareolar abscess andlactiferous duct fistula treated by a single breast surgeon. *American Journal of Surgery*.

[2] Katz U., Molad Y., Ablin J. (2007). Chronic idiopathic granulomatous mastitis. *Annals of the New York Academy of Sciences*.

[3] Bani-Hani K. E., Yaghan R. J., Matalka I. I., Shatnawi N. J. (2004). Idiopathic granulomatous mastitis: time to avoid unnecessary mastectomies. *The Breast Journal*.

[4] Lai E. C. H., Chan W. C., Ma T. K. F., Tang A. P. Y., Poon C. S. P., Leong H. T. (2005). The role of conservative treatment in idiopathic granulomatous mastitis. *The Breast Journal*.

[5] Asoglu O., Ozmen V., Karanlik H. (2005). Feasibility of surgical management in patients with granulomatous mastitis. *The Breast Journal*.

[6] Wu H. L., Yu J. J., Yu S. L., Zhou B. G., Bao S. L., Dong Y. (2016). Clinical efficacy of fiberoptic ductoscopy in combination with ultrasound-guided minimally invasive surgery in treatment of plasma cell mastitis. *Clinical and Experimental Obstetrics & Gynecology*.

[7] Ming J., Meng G., Yuan Q. (2013). Clinical characteristics and surgical modality of plasma cell mastitis: analysis of 91 cases. *The American Surgeon*.

[8] Liu L., Zhou F., Wang P. (2017). Periductal mastitis: an inflammatory disease related to bacterial infection and consequent immune responses?. *Mediators of Inflammation*.

[9] Console L., Scalise M., Indiveri C. (2019). Exosomes in inflammation and role as biomarkers. *Clinica Chimica Acta*.

[10] Scherer P. E. (2019). The many secret lives of adipocytes: implications for diabetes. *Diabetologia*.

[11] Tan L., Wu H., Liu Y., Zhao M., Li D., Lu Q. (2016). Recent advances of exosomes in immune modulation and autoimmune diseases. *Autoimmunity*.

[12] Turpin D., Truchetet M. E., Faustin B. (2016). Role of extracellular vesicles in autoimmune diseases. *Autoimmunity Reviews*.

[13] Dai Y. D., Sheng H., Dias P. (2017). Autoimmune responses to exosomes and candidate antigens contribute to type 1 diabetes in non-obese diabetic mice. *Current Diabetes Reports*.

[14] Wang X., Xu C., Hua Y. (2016). Exosomes play an important role in the process of psoralen reverse multidrug resistance of breast cancer. *Journal of Experimental & Clinical Cancer Research*.

[15] Yu J. J., Bao S. L., Yu S. L. (2012). Mouse model of plasma cell mastitis. *Journal of Translational Medicine*.

[16] Matsumoto A., Takahashi Y., Nishikawa M. (2017). Accelerated growth of B16BL6 tumor in mice through efficient uptake of their own exosomes by B16BL6 cells. *Cancer Science*.

[17] Trajkovic K., Hsu C., Chiantia S. (2008). Ceramide triggers budding of exosome vesicles into multivesicular endosomes. *Science*.

[18] Liu Y., Zhang D. T., Liu X. G. (2015). mTOR signaling in T cell immunity and autoimmunity. *International Reviews of Immunology*.

[19] Jones D. D., Gaudette B. T., Wilmore J. R. (2016). mTOR has distinct functions in generating versus sustaining humoral immunity. *The Journal of Clinical Investigation*.

[20] Dauner K., Eid W., Raghupathy R., Presley J. F., Zha X. (2017). mTOR complex 1 activity is required to maintain the canonical endocytic recycling pathway against lysosomal delivery. *The Journal of Biological Chemistry*.

[21] Zhang Y., Bai X., Wang Y. (2014). Role for heat shock protein 90*α* in the proliferation and migration of HaCaT cells and in the deep second-degree burn wound healing in mice. *PLoS One*.

[22] Buttgereit F., Brand M. D. (1995). A hierarchy of ATP-consuming processes in mammalian cells. *The Biochemical Journal*.

[23] Susorov D., Zakharov N., Shuvalova E. (2018). Eukaryotic translation elongation factor 2 (eEF2) catalyzes reverse translocation of the eukaryotic ribosome. *The Journal of Biological Chemistry*.

[24] Qiu P., Liu Y., Zhang J. (2019). Review: the role and mechanisms of macrophage autophagy in sepsis. *Inflammation*.

[25] Ito M., Yurube T., Kakutani K. (2017). Selective interference of mTORC1/RAPTOR protects against human disc cellular apoptosis, senescence, and extracellular matrix catabolism with Akt and autophagy induction. *Osteoarthritis and Cartilage*.

[26] Kim H., Banerjee N., Barnes R. C. (2017). Mango polyphenolics reduce inflammation in intestinal colitis-involvement of the miR-126/PI3K/AKT/mTOR axis in vitro and in vivo. *Molecular Carcinogenesis*.

[27] Liu Y., Zhang J., Zhou Y. H. (2015). IL-6/STAT3 signaling pathway is activated in plasma cell mastitis. *International Journal of Clinical and Experimental Pathology*.

[28] Hou Y., Wang K., Wan W., Cheng Y., Pu X., Ye X. (2018). Resveratrol provides neuroprotection by regulating the JAK2/STAT3/PI3K/AKT/mTOR pathway after stroke in rats. *Genes & Diseases*.

