# Supplementary material for: Exosomes Play an Important Role in the Progression of Plasma Cell Mastitis via the PI3K-Akt-mTOR Signaling Pathway
Source: Mediators Inflamm. 2019 Jun 9;2019:4312016. doi: 10.1155/2019/4312016 (PMC6590603; doi:10.1155/2019/4312016)
Supplement: Supplementary Materials — A total of 2978 DEGs (differential expression genes) including 581 downexpressed genes and 2397 upexpressed genes in PCM/exo. [file 4312016.f1.pdf]

down-expressed DEGs in PCM/exo

gene	N/EXO	PCM/EXO	log2(Fold_change)	p-value	q-value
A2M	17.2908	2.63411	-2.714617122	1.09E-08	5.23E-07
ACADM	19.6066	3.62633	-2.434757255	2.47E-05	0.000364094
ACADVL	69.6438	18.9947	-1.874397997	1.26E-09	7.61E-08
ACAT1	25.0722	3.42751	-2.870855813	2.45E-06	5.37E-05
ACLY	97.0387	11.8396	-3.034939883	3.12E-40	2.22E-37
ACO1	22.8722	5.23651	-2.126917623	2.89E-06	6.17E-05
ACSL1	86.3046	9.36099	-3.204704437	1.19E-41	9.04E-39
ACSS2	53.4167	12.5551	-2.089017332	7.51E-11	5.87E-09
ACTB	634.69	260.729	-1.283501054	3.90E-29	1.89E-26
ACTG1	231.503	114.676	-1.013467401	2.34E-09	1.33E-07
ADD3	13.7548	1.04289	-3.721276261	6.50E-10	4.17E-08
ADGRG6	9.1008	1.9816	-2.199327596	2.29E-05	0.000341997
ADH1A	45.4825	1.43687	-4.98431011	1.01E-12	1.04E-10
ADH1B	133.344	1.53184	-6.443743484	5.74E-73	6.68E-70
ADH1C	30.4917	3.67799	-3.051427125	9.57E-07	2.41E-05
ADH4	110.959	3.0046	-5.206709942	4.95E-41	3.60E-38
ADH5	16.5378	2.43812	-2.761926288	2.75E-05	0.000399206
AFF4	15.769	5.08453	-1.632904847	4.03E-07	1.14E-05
AGPS	17.8014	2.08079	-3.096787431	1.23E-14	1.63E-12
AHCYL1	21.5799	7.02815	-1.618471292	1.89E-05	0.000292237
AHSG	142.507	10.1394	-3.812988598	6.52E-29	3.12E-26
AIF1L	57.8658	10.9684	-2.399357845	3.22E-15	4.65E-13
AK4	25.1465	7.49262	-1.746815423	3.72E-09	2.02E-07
AKR1A1	67.8537	16.0965	-2.075680462	2.14E-07	6.70E-06
AKR1C3	24.6118	1.52395	-4.013462699	4.02E-08	1.63E-06
AKR1D1	19.7802	2.86313	-2.788391931	3.77E-06	7.69E-05
ALAS1	29.8111	8.08677	-1.882214126	5.62E-05	0.000710982
ALB	3643.89	2.58451	-10.46137291	0	0
ALDH1A1	32.9126	1.63763	-4.328958658	9.85E-13	1.02E-10
ALDH1A3	29.7142	8.48261	-1.808572401	3.03E-06	6.46E-05
ALDH1L1	41.6033	15.7017	-1.405777202	1.00E-05	0.000174357
ALDH9A1	44.8328	6.72454	-2.737047118	1.03E-10	7.81E-09
ALDOA	146.663	69.1461	-1.084785168	4.23E-10	2.82E-08
ALDOB	164.714	4.2644	-5.271476604	7.14E-68	8.05E-65
AMBP	163.117	11.1222	-3.874393057	1.35E-30	7.14E-28
ANAPC13	23.8101	2.80821	-3.083850949	6.84E-06	0.000126517
ANGPTL3	11.5594	0.438217	-4.721277253	5.24E-07	1.43E-05
AOX1	16.7903	2.88305	-2.541960245	1.05E-07	3.69E-06
APCS	94.2173	6.4821	-3.861458807	1.15E-12	1.18E-10
APOA1	142.008	17.1895	-3.04637272	8.46E-19	2.55E-16
APOA1-AS	60.5895	7.65648	-2.984314617	8.23E-05	0.000977166
APOA2	628.458	6.37932	-6.622269873	4.46E-59	4.58E-56
APOB	113.537	3.69367	-4.941963738	2.93E-251	7.30E-248
APOC1	209.79	29.6917	-2.820814309	6.44E-16	1.70E-13
APOC2	66.8468	8.86955	-2.913925683	5.25E-06	0.000101799
APOC3	265.142	12.9399	-4.356866846	1.25E-21	4.46E-19
APOE	110.146	19.4863	-2.498885017	5.27E-13	5.75E-11
APOH	332.72	2.51242	-7.049087121	4.46E-81	5.99E-78

APP	33.9845	4.93867	-2.78268242	6.73E-13	7.21E-11
ARCN1	25.482	4.61543	-2.464941546	8.79E-09	4.35E-07
ARF1	88.683	34.7123	-1.353210615	9.17E-07	2.33E-05
ARF4	47.611	13.13	-1.858428015	2.37E-05	0.000350829
ARG1	19.5446	1.44071	-3.761918188	2.67E-05	0.000388311
ARHGAP18	15.3199	3.07988	-2.314460834	4.70E-05	0.000613083
ARHGAP5	8.6259	1.12208	-2.942499453	4.33E-09	2.31E-07
ARHGEF12	14.101	2.62369	-2.426128303	3.16E-11	2.65E-09
ARL6IP1	43.4258	11.8579	-1.872703891	9.67E-07	2.43E-05
ARPC5	44.3861	14.0518	-1.659353003	4.60E-05	0.000609486
ASPH	11.7435	2.79397	-2.071474014	3.45E-09	1.88E-07
ATF4	69.7191	26.5824	-1.391082581	1.64E-05	0.000261552
ATP1B1	28.7958	3.72279	-2.951402261	2.17E-07	6.76E-06
ATP5A1	72.254	7.10148	-3.346885829	5.41E-20	1.77E-17
ATP5B	145.427	24.3246	-2.579807159	2.69E-21	9.41E-19
ATP5C1	74.7158	12.851	-2.539532732	4.76E-08	1.87E-06
ATP6V1A	19.0978	4.03561	-2.24254779	5.85E-07	1.58E-05
AZGP1	111.716	12.9488	-3.108945513	3.70E-15	5.28E-13
B4GALT1	71.9538	12.8322	-2.487302347	1.25E-22	4.63E-20
BAAT	19.3378	4.52075	-2.09678963	2.32E-05	0.00034746
BCHE	9.61549	0.510331	-4.235855193	4.03E-05	0.000544435
BHMT	38.985	5.86242	-2.733350899	1.55E-09	9.13E-08
BLOC1S5-T	24.7146	6.94428	-1.831466532	2.92E-05	0.000418702
BTBD1	23.656	4.05427	-2.544692073	2.31E-07	7.12E-06
BTF3	97.5406	24.5038	-1.99299735	9.04E-08	3.25E-06
BTN1A1	124.733	10.7312	-3.538959885	4.84E-41	3.60E-38
C1QBP	79.6126	17.0497	-2.223250425	3.53E-07	1.02E-05
C1R	38.7098	8.94078	-2.11422625	1.72E-06	3.95E-05
C1S	46.5585	5.18614	-3.166311521	1.52E-14	1.97E-12
C3	102.552	18.5628	-2.465869381	1.75E-37	1.13E-34
C4BPA	23.8985	1.72962	-3.788393054	2.58E-08	1.11E-06
C5	14.793	3.0303	-2.287382126	2.00E-07	6.31E-06
C6	17.1978	2.49921	-2.782679986	2.42E-07	7.38E-06
C8A	26.4844	5.04585	-2.3919736	6.89E-06	0.000127367
CALM1	37.7851	10.736	-1.815360864	1.35E-08	6.33E-07
CALM2	39.1456	14.1171	-1.471406402	2.02E-07	6.35E-06
CAND1	12.5173	2.21447	-2.498890046	3.26E-07	9.61E-06
CANX	64.9357	8.35489	-2.958319114	4.78E-30	2.42E-27
CAPRIN1	16.3361	3.58117	-2.189560692	3.53E-08	1.46E-06
CAPZA2	20.6839	1.64666	-3.65089363	1.82E-07	5.87E-06
CAT	29.6625	4.49802	-2.721278214	3.63E-07	1.04E-05
CCDC152	80.9629	1.48173	-5.771906477	6.37E-52	5.86E-49
CCDC186	9.10665	2.03755	-2.160084975	2.22E-06	4.92E-05
CCNB1	49.4278	6.95579	-2.829036412	9.88E-11	7.53E-09
CCNG1	25.6688	3.14387	-3.029402384	1.35E-07	4.59E-06
CCNI	51.0148	8.16564	-2.643277981	1.53E-09	9.09E-08
CCT5	47.1359	10.3702	-2.184382556	1.30E-12	1.32E-10
CCT6A	25.6095	6.14875	-2.058314031	3.00E-05	0.000428384
CCT8	48.4155	5.21368	-3.215095051	6.87E-13	7.34E-11
CD24P4	348.879	3.75867	-6.536360693	9.86E-147	1.72E-143
CD2AP	14.4069	1.27438	-3.498892502	2.10E-10	1.49E-08

CD302	11.0224	1.08334	-3.346880388	2.28E-06	5.05E-05
CD36	40.3925	1.03219	-5.290306977	2.56E-38	1.72E-35
CD55	28.1277	7.46424	-1.913924309	3.11E-06	6.56E-05
CD59	24.1244	7.78895	-1.630992299	1.06E-08	5.12E-07
CDC42	43.248	10.2471	-2.077417742	5.80E-09	3.00E-07
CDR1	37.5145	1.32736	-4.82081672	2.18E-09	1.25E-07
CEBPZ	20.4876	2.84784	-2.846810992	1.58E-07	5.21E-06
CEBPZOS	24.6118	5.87809	-2.065930821	7.76E-07	2.01E-05
CEL	185.857	13.5497	-3.777860211	1.53E-54	1.49E-51
CELP	79.1574	7.43961	-3.411425327	3.88E-10	2.61E-08
CENPF	16.0923	3.05627	-2.396526633	1.19E-12	1.22E-10
CFH	30.0673	0.689538	-5.446421452	2.67E-24	1.07E-21
CFHR1	30.0578	1.32941	-4.498881403	1.72E-07	5.55E-06
CFI	13.1057	0.772855	-4.083852836	1.20E-05	0.000201663
CHCHD2	131.161	43.7307	-1.584620459	5.36E-06	0.000103714
CHD1	6.79234	1.06814	-2.668807925	4.14E-05	0.000558438
CHP1	46.77	14.947	-1.645727477	3.41E-07	9.92E-06
CHRDL2	93.2404	16.6511	-2.485337706	1.32E-19	4.14E-17
CLINT1	24.1244	2.24066	-3.428497407	2.84E-12	2.81E-10
CLTC	17.5925	5.22071	-1.752642586	8.28E-08	3.03E-06
CLU	85.5711	11.354	-2.91392299	3.76E-26	1.64E-23
CMPK1	22.2264	4.17789	-2.411427881	6.32E-07	1.69E-05
CNBP	38.8813	10.9548	-1.827513311	2.15E-07	6.73E-06
CNDP2	35.4112	14.457	-1.292437527	3.06E-06	6.49E-05
CNOT7	14.6883	2.59855	-2.498888706	3.54E-06	7.32E-05
COPA	23.2218	7.91215	-1.553338122	5.15E-06	0.0001001
COPB2	27.3942	3.50017	-2.968373574	1.30E-09	7.81E-08
COPG1	38.0465	7.43207	-2.355927747	2.93E-09	1.63E-07
CP	30.7071	2.22238	-3.788394843	3.57E-21	1.23E-18
CPB2	35.8724	2.68497	-3.739896397	2.22E-09	1.26E-07
CPS1	70.3501	1.39841	-5.652693162	2.13E-77	2.66E-74
CRYBG3	10.4243	2.08474	-2.322011136	8.65E-09	4.29E-07
CS	27.8371	7.09743	-1.971640302	9.82E-06	0.000171242
CSDE1	48.5844	6.19655	-2.970956045	2.21E-20	7.43E-18
CSN1S1	894.152	0	#NAME?	2.81E-197	6.13E-194
CSN1S2AP	2195.72	2.2134	-9.954214182	0	0
CSN2	13485.4	4.43146	-11.5713286	0	0
CSN3	1375.09	2.13396	-9.331777197	1.50E-241	3.48E-238
CSRP2	36.9576	1.40106	-4.721280427	5.24E-07	1.43E-05
CTNNA1	33.0533	9.87763	-1.742557482	6.25E-09	3.20E-07
CTNND1	29.162	6.24528	-2.223251507	1.26E-12	1.28E-10
CYB5A	135.679	16.0022	-3.083855275	9.40E-13	9.80E-11
CYP2C8	48.1534	2.98164	-4.013459683	2.16E-14	2.73E-12
CYP2E1	163.704	6.20602	-4.721277417	6.30E-43	5.11E-40
CYP3A4	34.9078	2.77904	-3.650890931	4.19E-13	4.64E-11
CYP3A5	21.8417	2.14671	-3.346885934	5.95E-11	4.76E-09
DAB2	59.3121	8.71301	-2.767083349	1.04E-23	4.04E-21
DAP	122.765	12.8839	-3.252258037	2.05E-30	1.07E-27
DDX17	22.3538	5.75226	-1.958319309	6.92E-07	1.82E-05
DDX3X	38.3312	6.20552	-2.626895151	4.39E-21	1.50E-18
DDX5	39.4025	6.31141	-2.642252917	2.07E-15	3.08E-13

DDX6	13.106	2.38682	-2.457065761	2.07E-07	6.49E-06
DDX60L	9.45845	1.43428	-2.721277087	3.63E-07	1.04E-05
DEK	30.5525	4.204	-2.861455861	3.17E-09	1.74E-07
DHX15	27.4679	4.57358	-2.586350825	7.34E-08	2.71E-06
DIAPH3	12.3202	3.01792	-2.029399208	6.54E-05	0.000810727
DLST	24.598	6.52755	-1.91392751	9.03E-06	0.000159798
DNAJA1	55.0751	8.84133	-2.639064894	9.95E-12	8.93E-10
DNAJA2	15.988	1.97993	-3.013468148	3.27E-06	6.89E-05
DNAJB6	30.5525	6.48617	-2.235851693	1.61E-07	5.28E-06
DNAJC12	22.3245	0.846322	-4.721277384	5.24E-07	1.43E-05
DPYSL2	42.8039	9.63033	-2.152085111	2.90E-14	3.63E-12
DPYSL3	45.0173	10.4147	-2.111858248	1.76E-15	2.69E-13
DSC2	12.9851	2.0511	-2.662387388	5.37E-07	1.46E-05
DSTN	86.5217	11.6036	-2.898489543	8.61E-15	1.17E-12
EDF1	89.9931	23.0854	-1.96283366	1.99E-06	4.49E-05
EEF1A1	960.195	67.752	-3.824991984	0	0
EEF1B2	96.5845	25.6306	-1.91392443	1.07E-06	2.67E-05
EEF1G	205.951	67.8261	-1.602388686	9.30E-13	9.75E-11
EEF2	378.061	37.9464	-3.316584104	2.31E-122	3.85E-119
EHF	39.7454	3.74502	-3.407742569	1.62E-25	6.84E-23
EHHADH	17.1719	4.2314	-2.020842699	3.84E-05	0.000526589
EID1	74.5768	8.03986	-3.213484609	3.56E-17	9.95E-15
EIF3E	42.0097	8.57546	-2.292436517	1.28E-05	0.00021476
EIF3H	68.7971	11.4949	-2.581353837	3.34E-08	1.39E-06
EIF4A1	83.0794	27.5584	-1.591998667	4.63E-07	1.29E-05
EIF4A2	28.3471	5.47088	-2.373356334	3.43E-05	0.000477807
EIF4B	74.2526	5.9325	-3.645729443	2.20E-36	1.39E-33
EIF4G2	58.1332	10.3834	-2.4850834	2.86E-19	8.75E-17
EIF4H	63.1631	11.8516	-2.414000139	1.90E-12	1.90E-10
ELF5	115.441	5.18946	-4.475427468	2.47E-44	2.05E-41
ELL2	22.5682	4.99077	-2.176957033	1.64E-09	9.60E-08
ELOVL5	27.1277	7.30795	-1.892228067	1.33E-06	3.19E-05
ENO1	211.224	59.4977	-1.827867964	3.07E-31	1.65E-28
ENSG0000C	58.9366	15.3656	-1.939459732	4.25E-06	8.55E-05
ENSG0000C	194.089	57.0521	-1.76636646	1.13E-14	1.51E-12
ENSG0000C	31.0332	4.60206	-2.75346077	4.60E-15	6.45E-13
ENSG0000C	61.1434	2.70427	-4.498885673	6.44E-09	3.29E-07
ENSG0000C	119.756	22.0012	-2.44444382	4.97E-06	9.80E-05
ENSG0000C	37.1286	4.10533	-3.176960816	1.41E-05	0.000231601
ENSG0000C	808.339	364.664	-1.148392673	2.19E-08	9.59E-07
ENSG0000C	19.1103	2.53565	-2.9139229	5.25E-06	0.000101799
ENSG0000C	69.492	2.30513	-4.913926882	5.70E-08	2.19E-06
ENSG0000C	638.148	150.285	-2.086190034	1.20E-21	4.31E-19
EPRS	16.5191	2.75053	-2.586353544	7.34E-08	2.71E-06
ERP29	66.211	14.642	-2.17695829	2.79E-07	8.39E-06
EZR	40.3974	13.1025	-1.624420334	2.30E-06	5.09E-05
F9	13.9133	1.69224	-3.039458547	2.37E-05	0.000350829
FABP3	472.062	14.4543	-5.029405706	2.92E-102	4.08E-99
FAM107B	27.2189	9.21565	-1.562450933	2.16E-06	4.83E-05
FAM162A	58.1519	7.71587	-2.913925588	5.25E-06	0.000101799
FAM46A	15.6301	3.6869	-2.083846817	2.21E-06	4.91E-05

FASN	1536.03	17.7867	-6.432263714	0	0
FBXL3	19.2234	2.30773	-3.058317179	3.98E-08	1.62E-06
FBXO7	39.9954	13.2204	-1.597068252	7.70E-05	0.0009306
FDPS	82.8461	19.891	-2.058317976	7.38E-09	3.71E-07
FGA	161.37	3.70476	-5.444848499	8.45E-110	1.23E-106
FGB	127.604	4.39615	-4.859289131	3.76E-73	4.53E-70
FGG	100.329	5.07129	-4.306242089	4.53E-30	2.32E-27
FGL1	34.4501	0.831092	-5.373356172	6.40E-11	5.09E-09
FKBP4	40.3599	11.1709	-1.853177177	2.13E-08	9.38E-07
FLJ41200	31.399	2.40712	-3.705338643	1.91E-10	1.36E-08
FN1	49.853	6.7106	-2.893166654	6.71E-40	4.69E-37
FTH1	2129.27	931.566	-1.19262901	1.92E-56	1.92E-53
G3BP2	20.3449	5.11475	-1.991931559	2.69E-06	5.82E-05
GALNT1	12.6971	0.786196	-4.013466191	4.02E-08	1.63E-06
GAPDH	533.159	262.008	-1.024954978	6.06E-18	1.76E-15
GAS5-AS1	35.109	1.24225	-4.820813458	1.44E-05	0.000235016
GATM	37.0074	4.05919	-3.188550005	1.02E-13	1.20E-11
GC	105.33	2.47189	-5.413158048	2.95E-42	2.29E-39
GLS	9.20202	2.09309	-2.136316245	1.79E-05	0.000280786
GLUD1	23.9348	6.35157	-1.9139246	3.11E-06	6.56E-05
GMFB	14.1764	2.40349	-2.56028851	5.52E-06	0.000106707
GNB1	41.1943	13.7658	-1.581356273	3.75E-06	7.66E-05
GNG12	25.4212	3.51965	-2.852528257	3.03E-11	2.56E-09
GNL3	27.3004	3.42111	-2.996385692	4.57E-07	1.28E-05
GOLGA7	28.0065	4.54182	-2.624419264	3.81E-06	7.76E-05
GPAM	11.4446	2.15969	-2.405770891	1.40E-06	3.34E-05
GPX4	114.621	47.008	-1.285893182	1.78E-05	0.000279296
GSTA1	68.0805	2.21869	-4.939461622	1.78E-16	4.83E-14
GSTT1	28.3793	0	#NAME?	3.98E-09	2.14E-07
H19	157.621	16.2664	-3.276492858	8.56E-43	6.80E-40
H3F3A	78.0068	10.5533	-2.885905694	6.76E-09	3.42E-07
H3F3B	54.5055	19.4361	-1.487663055	1.83E-06	4.18E-05
HBP1	23.6642	4.78457	-2.306244973	5.78E-06	0.000110263
HDLBP	54.8713	16.4658	-1.736579148	5.10E-17	1.40E-14
HECTD1	14.8684	3.94563	-1.913921827	4.51E-08	1.80E-06
HIBADH	36.585	3.23619	-3.498884041	7.20E-10	4.58E-08
HIF1A	28.8285	7.84637	-1.877398499	1.98E-07	6.29E-06
HIF1A-AS2	35.6396	2.52205	-3.820812362	9.26E-11	7.10E-09
HIST1H1C	179.747	58.2991	-1.624422175	2.30E-06	5.09E-05
HIST1H2AH	162.1	10.7541	-3.913925394	1.29E-11	1.13E-09
HIST1H2AM	130.084	26.554	-2.29244242	1.28E-05	0.00021476
HIST1H2BC	189.14	54.1285	-1.804994188	3.58E-05	0.000499035
HIST1H2BD	101.759	23.0032	-2.145249923	1.32E-06	3.17E-05
HIST1H2BJ	324.2	11.6503	-4.79844508	8.95E-26	3.81E-23
HIST1H3B	206.488	21.005	-3.297253251	9.03E-12	8.15E-10
HIST1H3D	100.531	21.0616	-2.254953499	2.66E-07	8.04E-06
HIST1H3G	146.089	12.9226	-3.498879281	2.47E-09	1.40E-07
HIST1H3H	185.447	28.2513	-2.714617769	1.09E-08	5.23E-07
HIST1H4A	78.5988	6.95258	-3.498886941	5.37E-05	0.000688905
HIST1H4B	95.5515	8.45216	-3.498886538	1.51E-05	0.000246127
HIST1H4C	162.438	22.1056	-2.877405371	3.40E-07	9.91E-06

HIST1H4E	103.409	4.57358	-4.498894056	1.72E-07	5.55E-06
HIST1H4H	91.2083	6.9154	-3.721280509	7.62E-06	0.000138493
HMGCS1	18.658	4.80122	-1.958321399	6.92E-07	1.82E-05
HMGCS2	33.5942	8.21566	-2.031763792	6.12E-06	0.000115292
HMGN1	41.1077	8.26419	-2.314463326	4.70E-05	0.000613083
HNRNPA1	113.54	22.0953	-2.361389222	2.18E-17	6.25E-15
HNRNPA2B1	39.4905	5.12335	-2.946346277	1.17E-14	1.55E-12
HNRNPC	35.964	11.532	-1.640910748	6.62E-06	0.000122725
HNRNPF	34.7151	12.0272	-1.529262513	2.06E-05	0.000313817
HNRNPH1	55.3281	5.45884	-3.341346065	5.92E-15	8.14E-13
HNRNPK	39.2872	12.8316	-1.614358276	3.86E-06	7.86E-05
HNRNPU	24.9137	7.87065	-1.662384605	5.09E-08	1.99E-06
HOTS	62.685	13.1968	-2.247932116	2.82E-16	7.56E-14
HP	605.776	1.78616	-8.405779292	9.25E-187	1.70E-183
HPS3	15.3429	3.44514	-2.15493668	3.01E-05	0.000430346
HPX	105.089	16.2013	-2.69743018	4.62E-15	6.45E-13
HRG	45.9501	4.70636	-3.287384416	3.07E-11	2.59E-09
HSD17B6	24.0946	2.66415	-3.176962633	1.41E-05	0.000231601
HSDL2	20.2264	4.08951	-2.306239668	5.78E-06	0.000110263
HSP90AA1	118.419	21.9022	-2.43475288	6.25E-34	3.70E-31
HSP90AB1	132.98	25.0149	-2.410349693	4.16E-27	1.86E-24
HSP90B1	74.9188	10.3924	-2.849798926	9.14E-20	2.93E-17
HSPA13	15.8893	1.08116	-3.877403621	1.06E-09	6.47E-08
HSPA8	225.828	32.2419	-2.808215705	4.74E-47	4.14E-44
HSPA9	27.7988	8.2376	-1.754726628	1.30E-05	0.000216387
HSPD1	40.0093	7.96293	-2.328964107	6.78E-08	2.53E-06
IDH1	38.5505	5.11506	-2.913926504	4.18E-11	3.45E-09
IDH2	94.471	19.2665	-2.293777018	1.48E-12	1.49E-10
IER3IP1	32.4011	6.01879	-2.428497407	5.53E-05	0.000705723
IGFBP3	53.7137	3.93213	-3.771907179	9.17E-19	2.74E-16
IL33	24.1757	1.68025	-3.846809854	3.13E-10	2.14E-08
IL6ST	15.5246	2.22562	-2.802276902	2.09E-13	2.37E-11
ILF2	28.124	4.97551	-2.498885482	1.75E-05	0.000275648
IPO5	11.3668	1.72367	-2.721270662	3.63E-07	1.04E-05
IQGAP1	22.9801	4.60355	-2.319566355	3.93E-12	3.79E-10
IRX2	44.6345	10.1525	-2.136324238	2.74E-09	1.53E-07
ITGB1	40.5398	6.32826	-2.679458191	1.45E-14	1.90E-12
ITIH2	22.9216	3.2441	-2.820817556	7.36E-08	2.72E-06
ITM2B	38.5532	7.95733	-2.276494276	3.51E-06	7.27E-05
IVNS1ABP	24.3714	6.77541	-1.846808972	3.44E-06	7.16E-05
KCTD3	24.1843	2.03229	-3.572892591	8.14E-13	8.62E-11
KIAA1522	45.202	13.9176	-1.699476158	1.87E-11	1.62E-09
KLF9	17.7783	5.79382	-1.617530608	6.50E-05	0.000806613
KNG1	53.8624	4.38834	-3.617531302	6.61E-23	2.51E-20
KPNB1	26.2643	8.32498	-1.657584423	5.57E-06	0.000106758
KRT18	88.8909	33.1369	-1.423597091	1.56E-05	0.000250465
KRT19	102.697	42.7837	-1.263260879	4.05E-05	0.000547172
KRT8	94.6656	30.6405	-1.627400428	2.23E-09	1.27E-07
KYNU	36.2944	5.56483	-2.705337446	1.66E-07	5.43E-06
LALBA	9137.12	4.14481	-11.10621782	0	0
LARP4	12.315	1.5379	-3.001383026	2.73E-09	1.53E-07

LDHA	93.5305	27.6905	-1.756045796	2.13E-11	1.82E-09
LDHB	103.619	14.827	-2.804989939	2.07E-15	3.08E-13
LIFR	8.90026	1.86463	-2.254958098	2.66E-07	8.04E-06
LMAN1	14.1104	3.03124	-2.218778898	1.03E-05	0.000178321
LPL	55.9233	2.64595	-4.401591791	2.19E-31	1.20E-28
LRRC59	38.4501	14.344	-1.422539957	8.28E-05	0.000982592
LTF	464.65	12.4817	-5.218258061	9.35E-261	2.51E-257
LTN1	8.1648	1.55557	-2.39197423	6.89E-06	0.000127367
LUM	20.7269	1.426	-3.86145847	3.02E-07	8.98E-06
LUZP6	40.1494	8.65671	-2.213487693	7.48E-11	5.86E-09
MAL2	20.7514	3.2123	-2.691530133	2.59E-06	5.61E-05
MALAT1	155.425	6.87418	-4.49888714	2.62E-196	5.39E-193
MATR3	26.0378	6.54975	-1.991095811	1.84E-09	1.07E-07
MBNL3	4.93731	1.09184	-2.176963774	5.06E-05	0.000657738
MCL1	38.1738	15.0897	-1.339018683	1.22E-05	0.000204464
MFGE8	62.5795	26.2493	-1.253411181	4.55E-05	0.000603937
MICU1	38.2706	7.7378	-2.306241175	4.15E-08	1.68E-06
MIER1	8.8247	2.34181	-1.913923205	7.73E-05	0.000933751
MORF4L2	42.0097	4.88952	-3.102957734	1.49E-10	1.10E-08
MRC1	25.3466	1.2456	-4.346879482	3.12E-20	1.04E-17
MRPL30	13.9662	3.23105	-2.111864556	3.93E-05	0.000536192
MSMO1	34.9641	4.83251	-2.855029747	2.23E-08	9.76E-07
MTPN	40.1494	8.65671	-2.213487693	7.48E-11	5.86E-09
MYH9	36.1259	17.8039	-1.020840227	5.77E-06	0.000110263
MYOF	24.2125	5.92133	-2.031758885	3.27E-10	2.22E-08
NACA	39.1836	12.4777	-1.650897923	4.84E-07	1.34E-05
NAMPT	38.2873	14.8641	-1.365033813	2.59E-06	5.60E-05
NCKAP1	15.5474	4.03984	-1.944303277	2.11E-05	0.000320308
NCL	34.0778	10.9471	-1.638283465	5.48E-05	0.000700719
NCOA4	42.9771	11.088	-1.954568974	3.56E-10	2.41E-08
NDRG1	61.6497	23.0527	-1.419158141	8.97E-08	3.23E-06
NDUFS1	16.3711	2.29287	-2.835924797	1.13E-06	2.79E-05
NDUFV2	41.9194	6.02557	-2.79844844	6.03E-05	0.000757821
NEAT1	22.4983	6.57687	-1.774342935	9.90E-23	3.72E-20
NEK7	20.0395	2.50253	-3.001387244	2.73E-09	1.53E-07
NFIA	9.2377	3.0105	-1.617530577	6.50E-05	0.000806613
NIPSNAP1	37.0332	10.5983	-1.804986346	3.58E-05	0.000499035
NME1	99.2587	22.1592	-2.163287762	1.67E-07	5.45E-06
NME1-NME2	188.152	54.5898	-1.785195308	7.27E-12	6.64E-10
NME2	180.196	68.2627	-1.400397603	6.58E-08	2.47E-06
NORAD	31.01	4.59862	-2.753460633	4.60E-15	6.45E-13
NPAT	8.7389	1.89739	-2.203435445	8.49E-05	0.000998703
NPM1	172.476	18.8891	-3.190769756	1.35E-31	7.46E-29
NRIP1	9.67402	1.76851	-2.451581144	1.01E-06	2.52E-05
NUCKS1	23.3705	3.20094	-2.868121061	1.32E-14	1.74E-12
NUFIP2	8.06216	2.06021	-1.968375014	5.77E-06	0.000110263
NUP155	11.2923	1.99775	-2.498891403	1.75E-05	0.000275648
NUPR1	122.897	34.031	-1.852528248	1.19E-06	2.91E-05
NUSAP1	21.8437	1.75656	-3.636392487	5.26E-08	2.05E-06
OLAH	111.598	8.93145	-3.643272949	2.67E-25	1.10E-22
ORM1	92.9321	9.24801	-3.328962146	6.96E-10	4.46E-08

ORM2	63.5123	6.63955	-3.257878646	3.35E-07	9.75E-06
P4HB	73.7207	21.4025	-1.784290444	1.26E-09	7.61E-08
PABPC1	158.462	15.072	-3.394194141	3.35E-49	3.00E-46
PAPOLA	11.1285	2.33145	-2.254959751	2.66E-07	8.04E-06
PAPSS2	17.6837	3.57541	-2.306239571	5.78E-06	0.000110263
PCBP2	38.4195	10.3313	-1.894816942	2.72E-07	8.20E-06
PCNA	57.4502	17.0391	-1.75346278	5.32E-05	0.000683276
PDCD4	36.8977	3.14727	-3.551358034	4.63E-17	1.28E-14
PDCD6IP	11.7319	3.37272	-1.798452222	6.13E-05	0.000765813
PDHA1	34.7386	9.71022	-1.838963726	5.05E-07	1.40E-05
PEBP1	122.192	13.8969	-3.136314834	2.65E-21	9.36E-19
PGK1	74.4143	18.3241	-2.021837546	6.88E-11	5.45E-09
PGRMC1	50.7882	4.71717	-3.428499844	2.84E-12	2.81E-10
PIGR	133.37	16.6374	-3.002932286	1.21E-52	1.14E-49
PJA2	22.0187	3.18713	-2.788399522	1.49E-10	1.10E-08
PKM	99.7716	43.4968	-1.197719943	6.54E-09	3.33E-07
PLG	43.8916	3.1673	-3.792619434	4.19E-24	1.66E-21
PLIN2	176.529	23.2172	-2.926639309	8.08E-34	4.71E-31
PLIN3	49.9808	18.6056	-1.425637078	5.76E-05	0.000728906
PON1	27.8147	4.92078	-2.498888623	3.92E-05	0.000536192
PPDPF	175.608	27.7387	-2.662386489	3.17E-12	3.12E-10
PPIA	213.262	36.6709	-2.539919343	1.07E-38	7.31E-36
PPP1CB	25.3504	4.48482	-2.498886523	1.25E-10	9.27E-09
PPP1CC	32.9921	2.23169	-3.885912067	3.84E-12	3.74E-10
PPP1R3C	34.1043	3.18435	-3.420882831	3.31E-11	2.77E-09
PPP2R5C	16.4502	3.16704	-2.37689813	3.93E-07	1.12E-05
PRDX1	112.067	26.8613	-2.060760482	1.22E-09	7.42E-08
PRDX3	74.879	6.3586	-3.557780107	1.80E-15	2.71E-13
PRDX6	43.7706	8.00171	-2.451581912	1.01E-06	2.52E-05
PRELID3B	24.7174	3.86827	-2.675766594	1.18E-06	2.89E-05
PRKD3	6.60427	0.803263	-3.039454773	2.37E-05	0.000350829
PRPS2	28.7783	6.10951	-2.23585279	4.69E-06	9.28E-05
PSAP	137.982	42.1236	-1.711779434	3.14E-17	8.91E-15
PSMC2	22.7716	4.70003	-2.276493792	3.51E-06	7.27E-05
PTBP3	8.87745	2.46051	-1.85118795	6.96E-05	0.000851569
PTMA	137.158	29.6175	-2.211316999	2.09E-11	1.80E-09
PTP4A1	31.6002	5.33637	-2.566003084	1.91E-13	2.17E-11
PYROXD1	12.9919	0.731318	-4.150969751	5.18E-09	2.70E-07
RAB18	15.3183	3.3875	-2.176963347	3.71E-06	7.60E-05
RAB31	18.3017	4.31707	-2.083853272	1.37E-05	0.000226879
RACK1	307.591	68.4099	-2.168736265	5.83E-21	1.98E-18
RAD21	14.2945	2.52888	-2.498889672	1.75E-05	0.000275648
RAD23A	45.4932	13.9662	-1.70371138	7.76E-05	0.000933973
RALB	23.7083	4.9569	-2.257882159	6.35E-05	0.00078845
RAN	49.7649	11.513	-2.111864732	1.27E-08	6.01E-07
RAPGEF2	29.2916	2.21186	-3.727155022	2.29E-25	9.50E-23
RARS	22.7716	3.62574	-2.650888284	1.87E-05	0.000289966
RBBP4	17.0177	4.40845	-1.948692663	2.93E-08	1.24E-06
RBM39	20.6426	4.95621	-2.058315472	3.00E-05	0.000428384
RBM47	20.4285	6.32462	-1.691532569	9.41E-06	0.00016447
RBP4	117.627	12.6345	-3.218778776	2.68E-15	3.92E-13

RECQL	18.0534	4.44862	-2.020840791	3.84E-05	0.000526589
REEP5	21.8947	5.67184	-1.948692942	6.16E-05	0.00076833
RHOA	87.6219	23.6497	-1.889469616	2.14E-10	1.51E-08
RIDA	62.6614	5.96918	-3.391972339	1.57E-08	7.14E-07
RMRP	23947.4	355.464	-6.074021762	0	0
RN7SK	49318.4	764.742	-6.011009076	0	0
RN7SL1	1018270	41727.8	-4.608967481	0	0
RN7SL2	914861	25423.9	-5.169295303	0	0
RN7SL4P	1881.17	69.7303	-4.753700731	4.75E-79	6.15E-76
RNF145	15.8218	4.09867	-1.948685998	6.16E-05	0.00076833
RNU4-1	1218.28	56.876	-4.420881933	1.01E-26	4.47E-24
RNU4-2	1762.62	574.747	-1.616722545	1.66E-10	1.21E-08
RNU5A-1	833.013	81.054	-3.361383721	5.12E-12	4.80E-10
RNU5B-1	499.808	70.0012	-2.835922435	1.13E-06	2.79E-05
RNVU1-7	178.285	0	#NAME?	4.87E-08	1.91E-06
RNY1	1638.75	41.9616	-5.287382281	1.15E-32	6.48E-30
RNY3	1242.17	67.6172	-4.199328544	6.16E-19	1.87E-16
RPL10	160.238	38.02	-2.075385882	1.60E-23	6.12E-21
RPL10A	243.995	58.1541	-2.06889877	5.71E-11	4.57E-09
RPL11	199.844	71.3901	-1.485078331	1.58E-05	0.000252401
RPL12	265.807	97.315	-1.449644996	7.77E-07	2.01E-05
RPL13A	338.354	134.683	-1.328965682	7.43E-12	6.77E-10
RPL14	156.061	30.2811	-2.36562053	1.67E-11	1.46E-09
RPL17	134.084	40.0296	-1.743997989	5.90E-08	2.26E-06
RPL17-C18	66.5923	22.0895	-1.591994606	2.24E-06	4.97E-05
RPL19	233.081	61.0891	-1.931844514	3.75E-14	4.66E-12
RPL22	72.4542	16.126	-2.167680695	2.74E-10	1.89E-08
RPL3	320.505	53.4798	-2.583280888	6.16E-33	3.52E-30
RPL36AL	160.467	29.1358	-2.461411809	4.25E-08	1.72E-06
RPL36AP42	206.27	36.4918	-2.49888979	3.26E-07	9.61E-06
RPL39	325.651	100.821	-1.691530488	6.86E-07	1.80E-05
RPL4	240.106	34.401	-2.803149047	5.07E-30	2.53E-27
RPL41	792.718	389.725	-1.024351255	4.12E-09	2.21E-07
RPL5	160.705	40.1375	-2.001392151	5.18E-10	3.38E-08
RPL6	107.435	31.1903	-1.784294734	1.26E-09	7.61E-08
RPL7	279.463	27.2953	-3.355932829	4.54E-26	1.96E-23
RPL7A	355.903	50.8554	-2.807011216	1.05E-27	4.89E-25
RPLP0	296.86	134.706	-1.1399686	5.72E-09	2.97E-07
RPN1	43.7145	14.7308	-1.569276115	4.42E-05	0.000587648
RPPH1	21636.1	4850.37	-2.157273761	0	0
RPS11	384.721	152.806	-1.332111389	5.15E-08	2.01E-06
RPS12	397.213	125.119	-1.666611953	3.83E-09	2.07E-07
RPS13	230.299	79.8561	-1.528033626	1.44E-05	0.000234282
RPS20	147.559	36.2208	-2.026401601	1.15E-11	1.02E-09
RPS26	121.471	32.2347	-1.913925464	1.54E-05	0.000248612
RPS3A	129.566	35.2319	-1.878732999	1.30E-13	1.51E-11
RPS4X	259.968	25.7013	-3.33842081	2.11E-27	9.70E-25
RPS6	299.794	67.0769	-2.160083589	1.08E-15	2.44E-13
RPS8	380.173	79.4862	-2.257879761	7.98E-18	2.30E-15
RRM1	53.1763	4.22135	-3.655007042	1.01E-23	3.96E-21
SCARNA10	590.682	13.0624	-5.498889751	2.85E-35	1.75E-32

SCARNA12	126.34	15.9652	-2.984308946	8.23E-05	0.000977166
SCARNA13	336.689	0	#NAME?	3.54E-22	1.30E-19
SCARNA17	69.4508	5.11948	-3.761922128	2.67E-05	0.000388311
SCARNA2	406.094	39.0007	-3.380241788	1.03E-19	3.27E-17
SCARNA5	192.822	6.2023	-4.958322545	2.37E-10	1.66E-08
SCARNA6	201.52	3.24105	-5.95831794	1.38E-11	1.21E-09
SCARNA7	236.273	3.91873	-5.91392479	5.81E-16	1.55E-13
SCD	24.9538	7.56799	-1.72127744	4.79E-07	1.33E-05
SCP2	28.9754	2.33005	-3.636395759	3.65E-14	4.55E-12
SEC14L1	42.9041	13.1795	-1.702819882	4.76E-12	4.53E-10
SEC24D	17.8677	4.84692	-1.882213761	5.62E-05	0.000710982
SELENOM	101.806	22.8138	-2.157843915	8.15E-06	0.000146188
SELENOP	141.571	1.17402	-6.913924972	3.51E-63	3.83E-60
SEPHS2	37.3102	8.98424	-2.054101721	2.83E-06	6.06E-05
SERBP1	22.334	4.39727	-2.344561635	2.25E-11	1.92E-09
SERP1	43.0163	8.01782	-2.423601504	6.90E-11	5.45E-09
SERPINA1	105.736	28.41	-1.895995919	1.50E-19	4.67E-17
SERPINC1	73.1426	9.43533	-2.954567029	3.85E-12	3.74E-10
SET	39.5271	8.6162	-2.197718469	1.25E-09	7.59E-08
SF3B1	14.6928	2.67156	-2.459355032	9.37E-08	3.34E-06
SFXN1	22.6657	5.91454	-1.938172844	2.50E-06	5.48E-05
SH3BP4	38.5488	7.06928	-2.44705076	2.75E-15	4.00E-13
SKIL	10.7175	2.19233	-2.289431536	1.59E-06	3.71E-05
SKP1	28.8885	6.86757	-2.07262368	8.12E-06	0.000145923
SLC25A3	57.4154	13.755	-2.06148161	7.53E-08	2.78E-06
SLC2A2	29.7574	1.62948	-4.190764929	1.21E-15	2.44E-13
SLC38A2	33.9243	3.44212	-3.300949748	1.07E-18	3.18E-16
SLC38A4	22.0227	1.40692	-3.968379153	5.27E-13	5.75E-11
SLC39A6	15.895	3.2807	-2.276497437	2.85E-05	0.000409
SLC39A9	12.6269	2.82955	-2.157855954	8.15E-06	0.000146188
SLC40A1	13.0725	1.67028	-2.968373194	1.12E-05	0.000190868
SLC9A3R1	53.179	13.043	-2.027580909	3.46E-07	1.00E-05
SLCO1B1	15.7367	0.618672	-4.668814486	1.96E-08	8.68E-07
SLCO1B3	12.9346	0.715096	-4.176954696	4.45E-07	1.24E-05
SND1	39.8457	16.4482	-1.27649433	4.56E-05	0.000605562
SNHG10	47.2154	7.69357	-2.617532389	1.06E-08	5.13E-07
SNORA23	360.972	9.12296	-5.306241132	2.74E-13	3.06E-11
SNORA48	433.167	60.6677	-2.835922815	1.13E-06	2.79E-05
SNORA53	331.373	10.3454	-5.001394725	4.40E-15	6.25E-13
SNORA63	252.681	19.1582	-3.721283262	7.62E-06	0.000138493
SNORA73A	1082.92	8.32966	-7.022453345	2.74E-46	2.33E-43
SNORA73B	2128.14	35.467	-5.906971948	2.63E-62	2.78E-59
SNORA81	219.017	0	#NAME?	3.26E-10	2.22E-08
SNORD133	315.851	0	#NAME?	9.71E-17	2.65E-14
SNORD16	243.656	12.9318	-4.235850744	4.03E-05	0.000544435
SNORD3A	1302.5	160.903	-3.017020242	2.59E-27	1.17E-24
SOD1	90.8036	24.0965	-1.913925885	9.03E-06	0.000159798
SOSTDC1	28.6808	4.61273	-2.636392512	8.43E-06	0.00015112
SOX9	32.1986	8.87315	-1.859479699	2.43E-07	7.40E-06
SPOPL	9.34852	1.35317	-2.788394887	3.77E-06	7.69E-05
SPP1	478.179	30.8918	-3.952254932	6.67E-110	1.01E-106

SQSTM1	59.8736	21.0557	-1.507709178	5.58E-08	2.15E-06
SRP9	66.1783	8.78085	-2.913925725	4.18E-11	3.45E-09
SRSF1	21.5893	3.81942	-2.498890988	2.73E-10	1.89E-08
SRSF2	32.8932	10.7656	-1.611360639	4.55E-05	0.000603937
SRSF5	37.7987	9.00185	-2.070043187	5.09E-05	0.000658307
SRSF7	23.4944	3.98328	-2.560288124	5.52E-06	0.000106707
ST13	32.6599	8.06507	-2.017761413	1.21E-07	4.16E-06
STAG2	6.63512	1.04341	-2.668816393	4.14E-05	0.000558438
STK3	14.5989	2.58274	-2.498885347	3.92E-05	0.000536192
SUB1	23.6222	4.79365	-2.300946851	7.21E-07	1.87E-05
SUCLG2	23.7892	4.20862	-2.498887598	3.54E-06	7.32E-05
TALAM1	165.018	7.90887	-4.38300801	6.44E-188	1.25E-184
TARS	26.1665	6.53534	-2.001386765	7.78E-06	0.000141309
TAX1BP1	57.1277	3.76061	-3.925151845	5.26E-28	2.48E-25
TBC1D4	19.717	6.2385	-1.660168967	2.33E-06	5.15E-05
TBL1XR1	8.24058	0.728933	-3.498887763	2.47E-09	1.40E-07
TC2N	16.8436	1.09784	-3.939460805	1.59E-13	1.82E-11
TF	272.378	9.57568	-4.83009143	5.24E-118	8.31E-115
TFRC	33.1317	3.90763	-3.083846455	3.56E-18	1.04E-15
THRSP	61.9465	9.49793	-2.705337743	1.66E-07	5.43E-06
TIMM23	32.4875	3.83164	-3.083850804	6.84E-06	0.000126517
TM9SF3	12.6987	2.10616	-2.591993868	1.61E-07	5.28E-06
TMBIM6	68.247	14.1289	-2.27211648	1.24E-15	2.44E-13
TMED2	40.7983	6.21528	-2.714617747	1.09E-08	5.23E-07
TMED7	19.1903	4.88032	-1.975329612	1.67E-05	0.000266218
TMEM236	14.2959	3.16142	-2.17695683	3.71E-06	7.60E-05
TMPO	18.1183	3.51357	-2.36643805	6.12E-10	3.94E-08
TMSB10	768.377	265.612	-1.532494008	1.07E-13	1.25E-11
TMX2-CTNN	30.4035	6.55106	-2.214437147	7.64E-13	8.11E-11
TNPO1	10.0731	2.52458	-1.996392442	4.58E-06	9.12E-05
TOP2A	21.1765	4.0461	-2.387860282	9.86E-10	6.11E-08
TPM4	61.6646	28.8522	-1.095761185	6.19E-05	0.000771635
TPT1	176.137	52.7408	-1.739706639	2.54E-35	1.58E-32
TRIP12	12.0622	2.56075	-2.235854743	5.69E-09	2.95E-07
TRNA	2471.88	399.824	-2.628171728	1.40E-14	1.84E-12
TRNN	1802.39	330.676	-2.446420969	1.10E-10	8.23E-09
TRNP	2651.56	88.7476	-4.900989479	5.20E-30	2.56E-27
TRNS1	2189.38	81.2142	-4.752646568	7.87E-25	3.19E-22
TRNY	3913.27	711.902	-2.458624094	2.50E-19	7.72E-17
TRPS1	10.0764	3.14085	-1.681753349	1.60E-05	0.00025544
TTC37	11.1064	1.058	-3.391979728	1.57E-08	7.14E-07
TTR	225.559	5.10405	-5.46571858	1.34E-37	8.84E-35
TUBA1B	216.831	63.9336	-1.761924794	3.48E-17	9.81E-15
TWF1	25.3809	5.11385	-2.311261479	3.28E-07	9.64E-06
UAP1	25.5807	3.39417	-2.913925013	1.02E-07	3.60E-06
UBA3	13.7984	0.813704	-4.083853095	1.20E-05	0.000201663
UBB	236.763	78.2058	-1.598096131	4.53E-15	6.41E-13
UBC	229.989	77.374	-1.571644097	9.88E-22	3.59E-19
UGP2	99.9821	5.6602	-4.142744893	4.56E-34	2.75E-31
UGT2B10	35.4152	0.469905	-6.235855761	4.59E-20	1.51E-17
UGT2B15	23.7398	0.381807	-5.958320417	1.38E-11	1.21E-09

UGT2B4	30.0687	5.14222	-2.547799256	5.09E-07	1.40E-05
UGT2B7	49.041	0.456631	-6.746815782	6.96E-20	2.25E-17
UQCRC2	33.0606	3.89923	-3.083851746	1.18E-08	5.63E-07
VDAC1	47.8397	15.1958	-1.654535713	1.34E-05	0.000221422
VPS35	18.5811	3.11422	-2.576893141	1.52E-08	6.98E-07
VTN	63.8908	19.2667	-1.729498716	6.53E-06	0.000122249
VTRNA1-1	696.161	171.544	-2.020842328	3.84E-05	0.000526589
WDR26	9.92779	2.44635	-2.020841773	3.84E-05	0.000526589
XBP1	64.261	15.1582	-2.083844984	5.90E-08	2.26E-06
XDH	980.251	9.57576	-6.677620404	0	0
XRCC5	29.8355	4.90126	-2.605805369	2.21E-09	1.26E-07
YAP1	25.6301	4.68543	-2.451585747	1.05E-11	9.36E-10
YBX1	53.7022	25.335	-1.083849363	5.89E-05	0.000741325
YKT6	47.5685	16.6751	-1.51231112	8.17E-06	0.000146487
YME1L1	27.7008	4.90064	-2.498885566	2.73E-10	1.89E-08
YWHAE	62.8791	19.2355	-1.708809237	3.27E-06	6.89E-05
YWHAH	57.0751	10.3377	-2.464946241	8.79E-09	4.35E-07
YWHAZ	42.7116	16.4112	-1.379947209	7.29E-06	0.000133214
ZBTB41	6.2622	1.30929	-2.257884904	6.35E-05	0.00078845
ZBTB44	5.09795	0.766609	-2.733354383	1.25E-05	0.00020989
ZFP91	16.1728	4.81884	-1.746811669	2.21E-05	0.000331229
ZNF652	10.8505	2.80556	-1.951400853	8.38E-08	3.06E-06
ZNF664	23.6669	5.58264	-2.083851316	3.60E-07	1.04E-05

up-expressed DEGs in PCM/exo

gene	N/EXO	PCM/EXO	log2(Fold_change)	p-value	q-value
AARS2	1.01906	14.1523	3.795725634	3.92E-05	0.000536192
AATBC	0	21.8436	Inf	4.32E-09	2.31E-07
AATK	0	15.6047	Inf	1.20E-07	4.12E-06
ABCA2	0	14.3859	Inf	8.55E-11	6.57E-09
ABCA3	2.20737	15.295	2.792659727	5.17E-06	0.00010028
ABCA4	0	8.94364	Inf	4.32E-06	8.63E-05
ABCA7	0	17.9529	Inf	5.58E-11	4.48E-09
ABCB8	0	13.3824	Inf	6.61E-06	0.00012264
ABCB9	0	13.8945	Inf	3.96E-05	0.000536192
ABCC1	0.742402	11.4923	3.952323152	9.34E-06	0.000163578
ABCC10	0	16.0149	Inf	5.58E-08	2.15E-06
ABCC12	0	10.176	Inf	5.57E-05	0.000705723
ABCC8	0	20.1348	Inf	5.58E-09	2.90E-07
ABCC9	21.3052	949.982	5.478622678	1.33E-15	2.44E-13
ABCG4	0	22.451	Inf	4.70E-08	1.85E-06
ABLIM2	0	13.3221	Inf	2.82E-06	6.03E-05
ABTB2	0	17.6714	Inf	6.61E-08	2.47E-06
ACAN	0	12.7758	Inf	3.64E-10	2.46E-08
ACBD4	0	17.5617	Inf	2.38E-05	0.000350829
ACE	0	15.7755	Inf	1.55E-07	5.11E-06
ACHE	0	18.5778	Inf	2.18E-05	0.000327135
ACSF3	2.26868	18.2619	3.008911839	4.29E-05	0.000573472
ACTBP10	0	33.1776	Inf	2.18E-05	0.000327135
ACTN3	0	17.3407	Inf	2.00E-05	0.000306049
ACVR2B	0	7.16349	Inf	1.84E-07	5.88E-06

ADAM11	0	14.3264	Inf	6.61E-06	0.00012264
ADAM19	0.746382	12.6103	4.078544594	2.59E-06	5.61E-05
ADAM33	0	19.4322	Inf	2.38E-07	7.25E-06
ADAM8	0	15.9313	Inf	5.57E-05	0.000705723
ADAMTS10	1.11667	17.0883	3.935734074	1.10E-05	0.000187799
ADAMTS12	0	7.91948	Inf	1.42E-06	3.37E-05
ADAMTS13	0	17.7114	Inf	1.84E-08	8.19E-07
ADAMTS14	0	15.489	Inf	7.20E-08	2.67E-06
ADAMTS15	0	16.0214	Inf	2.82E-08	1.19E-06
ADAMTS16	0	10.8263	Inf	4.31E-05	0.000573472
ADAMTS17	0.766456	11.8647	3.952328839	9.34E-06	0.000163578
ADAMTS2	1.33969	15.3463	3.517919754	1.17E-07	4.07E-06
ADAMTS7	0	16.6273	Inf	2.38E-08	1.03E-06
ADAMTS8	0	12.8931	Inf	6.61E-05	0.000810778
ADAMTSL1	0	11.908	Inf	1.20E-08	5.70E-07
ADAMTSL4	0	15.7883	Inf	1.84E-06	4.18E-05
ADARB2	0.578344	14.0174	4.599147102	3.07E-09	1.69E-07
ADCY1	0.753654	12.8998	4.097302549	4.60E-12	4.41E-10
ADCY3	3.08882	19.308	2.644071026	2.75E-05	0.000399206
ADCY4	0	17.1497	Inf	6.07E-06	0.000114316
ADCY5	0	14.1918	Inf	4.32E-08	1.73E-06
ADCY7	0	11.9032	Inf	7.20E-07	1.87E-05
ADCY9	0	11.0457	Inf	8.54E-08	3.08E-06
ADCYAP1R1	0	15.1445	Inf	5.12E-09	2.68E-07
ADD2	0	12.7657	Inf	5.57E-07	1.50E-05
ADGRA1	0	15.521	Inf	3.64E-06	7.46E-05
ADGRA2	0.807612	17.431	4.43184918	3.55E-08	1.47E-06
ADGRB1	0	13.5681	Inf	6.61E-07	1.74E-05
ADGRB2	0	18.0665	Inf	6.61E-09	3.35E-07
ADGRD1	0	13.9077	Inf	5.12E-07	1.40E-05
ADGRF3	0	15.0879	Inf	7.20E-08	2.67E-06
ADGRG1	1.94226	17.7819	3.19460124	6.47E-06	0.000121228
ADGRL1	1.24046	14.8679	3.58325377	4.59E-08	1.82E-06
ADM2	0	12.8292	Inf	6.61E-05	0.000810778
ADORA1	0	20.6743	Inf	1.20E-05	0.000201663
ADRA2B	0	16.668	Inf	2.82E-05	0.000404432
ADRB3	0	20.1932	Inf	4.70E-05	0.000613083
AEBP1	0	17.618	Inf	1.20E-06	2.91E-05
AFF3	1.18669	10.6545	3.166447878	8.78E-06	0.000155394
AFG3L1P	0	14.9098	Inf	1.84E-05	0.000285786
AGAP1	0.428708	8.57038	4.321291929	1.53E-07	5.11E-06
AGRN	0	14.6893	Inf	8.55E-10	5.35E-08
AHNAK2	0	16.0027	Inf	1.33E-15	2.44E-13
AIM1L	0	20.875	Inf	7.21E-10	4.58E-08
AIPL1	0	22.3086	Inf	2.38E-07	7.25E-06
AJUBA	0	15.7563	Inf	1.69E-07	5.47E-06
AKAP2	1.37291	10.9905	3.000948063	4.63E-05	0.000611824
AKNA	2.6518	17.2799	2.704050993	1.43E-05	0.000233738
AKT2	2.75837	18.381	2.736327363	9.97E-06	0.000173762
ALG1L6P	0	16.9615	Inf	3.34E-05	0.000466589
ALG1L9P	0	14.7028	Inf	2.38E-05	0.000350829

ALK	0	13.4169	Inf	1.10E-07	3.84E-06
ALPK2	0	9.62108	Inf	1.69E-06	3.89E-05
ALPK3	1.78552	14.807	3.051863171	1.03E-09	6.30E-08
ALS2CL	0	15.0586	Inf	1.43E-08	6.56E-07
ALX4	0	17.3496	Inf	1.31E-08	6.13E-07
AMER1	0	12.5596	Inf	1.43E-09	8.49E-08
AMER2	0	7.19449	Inf	5.12E-07	1.40E-05
AMER3	0	10.8531	Inf	1.69E-06	3.89E-05
AMOTL1	0.541519	8.47845	3.968716678	7.96E-06	0.000143202
AMPD2	0	22.5828	Inf	1.01E-09	6.23E-08
AMZ1	0	13.0212	Inf	3.34E-07	9.75E-06
ANGPT4	0	12.3081	Inf	2.00E-05	0.000306049
ANK1	1.05013	14.5374	3.791129426	1.68E-09	9.85E-08
ANK2	1.34237	6.50108	2.275897028	8.04E-05	0.000955605
ANK3	0.514287	4.16252	3.016811613	3.98E-05	0.000538538
ANKH	0	6.46038	Inf	5.12E-05	0.000658307
ANKRD11	0	15.8792	Inf	2.33E-14	2.93E-12
ANKRD20A1	0	14.9721	Inf	5.12E-07	1.40E-05
ANKRD33B	1.04428	9.46826	3.180590734	7.54E-06	0.000137281
ANKRD34A	0	16.5884	Inf	1.31E-05	0.000216387
ANKRD35	0	17.0476	Inf	2.18E-05	0.000327135
ANKRD52	2.81488	12.0514	2.098055427	7.02E-05	0.000857252
ANKRD63	0	14.0107	Inf	1.55E-05	0.000248612
ANKS6	0	10.8711	Inf	3.64E-07	1.04E-05
ANO7	1.09558	17.9285	4.032488049	4.19E-06	8.44E-05
AOC3	3.51935	21.169	2.588572217	4.89E-05	0.0006363
AP3B2	0	18.5537	Inf	1.69E-06	3.89E-05
AP4S1	0	9.13523	Inf	2.00E-05	0.000306049
AP5B1	4.43348	17.6476	1.992960524	4.27E-05	0.000573472
AP5Z1	0	16.1677	Inf	3.96E-08	1.62E-06
APBA2	0	17.7683	Inf	5.12E-06	9.95E-05
APBB1	0	18.0006	Inf	4.70E-06	9.28E-05
APC2	0	15.9668	Inf	2.33E-14	2.93E-12
APOBEC3A	0	43.2851	Inf	7.84E-06	0.000141315
APOBEC3F	0	11.1341	Inf	2.82E-05	0.000404432
APOBR	1.28511	25.9182	4.334001769	1.30E-07	4.45E-06
APOL1	0	20.3117	Inf	8.54E-06	0.000151469
AQP2	0	12.7905	Inf	4.70E-05	0.000613083
AQP6	0	22.089	Inf	1.69E-05	0.000266218
AQP7P1	0	44.1904	Inf	1.55E-12	1.56E-10
AR	0.899267	8.47165	3.235821554	4.09E-06	8.24E-05
ARAP1	4.31556	21.1484	2.292928834	6.68E-06	0.000123809
ARAP3	0	15.7285	Inf	1.42E-07	4.77E-06
ARC	0	18.5701	Inf	3.64E-05	0.00050071
ARHGAP1	0	23.642	Inf	1.84E-07	5.88E-06
ARHGAP22	0	15.6486	Inf	1.84E-08	8.19E-07
ARHGAP23	0	19.7064	Inf	1.84E-10	1.32E-08
ARHGAP27	1.93878	15.4348	2.992965779	4.99E-05	0.000648193
ARHGAP27F	3.39118	28.0474	3.048007771	2.95E-05	0.000421596
ARHGAP30	0	12.9562	Inf	2.18E-05	0.000327135
ARHGAP31	0	10.7245	Inf	6.61E-08	2.47E-06

ARHGAP33	0	13.6145	Inf	1.31E-05	0.000216387
ARHGAP39	0	13.0872	Inf	2.18E-06	4.85E-05
ARHGAP4	0	15.468	Inf	6.07E-05	0.000757821
ARHGAP44	0	13.605	Inf	1.69E-05	0.000266218
ARHGAP45	2.68677	18.9536	2.818527166	6.56E-08	2.47E-06
ARHGAP6	0	12.2968	Inf	3.07E-07	9.07E-06
ARHGEF10L	2.68196	17.3183	2.690937694	1.66E-05	0.000264306
ARHGEF11	1.41476	11.1379	2.976848005	5.79E-05	0.00073122
ARHGEF15	0	14.01	Inf	1.42E-05	0.000232345
ARHGEF17	1.24744	15.2827	3.614857116	2.87E-08	1.21E-06
ARHGEF19	1.45858	19.0951	3.710566053	8.00E-05	0.000951668
ARHGEF4	0	17.4576	Inf	7.84E-07	2.02E-05
ARHGEF40	0	14.3303	Inf	7.84E-08	2.89E-06
ARHGEF9	0	8.75285	Inf	6.07E-05	0.000757821
ARID3B	0	13.9182	Inf	1.42E-05	0.000232345
ARID5A	0	20.6139	Inf	1.84E-05	0.000285786
ARL10	0	14.79	Inf	8.53E-05	0.000998703
ARMC5	0	17.3484	Inf	5.58E-08	2.15E-06
ARMCX4	0.641032	6.09563	3.249307053	3.51E-06	7.27E-05
ARNT2	0	12.684	Inf	1.31E-07	4.45E-06
ARPP21	0	7.90245	Inf	3.96E-05	0.000536192
ARSA	0	17.7818	Inf	4.70E-07	1.31E-05
ASAP3	0	14.7424	Inf	1.01E-05	0.000174751
ASB1	0.707275	12.7003	4.166447498	9.86E-07	2.47E-05
ASB6	2.10867	16.4142	2.960539196	6.73E-05	0.000824237
ASIC2	0	15.64	Inf	3.34E-06	6.95E-05
ASIC4	0	27.192	Inf	3.07E-07	9.07E-06
ASTN1	0	7.16102	Inf	3.34E-05	0.000466589
ASTN2	0	20.6199	Inf	1.82E-14	2.32E-12
ATAD3C	0	17.5373	Inf	2.82E-06	6.03E-05
ATCAY	0.967658	15.236	3.976843138	7.35E-06	0.000133983
ATG2A	0.764652	18.5329	4.599141526	3.07E-09	1.69E-07
ATG4B	0	18.1468	Inf	5.57E-05	0.000705723
ATG9A	0	13.3085	Inf	7.84E-05	0.000933973
ATG9B	0.937861	17.1727	4.194598959	7.14E-07	1.87E-05
ATOH8	1.66233	15.2191	3.194604331	6.47E-06	0.000121228
ATP10A	0	12.1043	Inf	6.61E-06	0.00012264
ATP13A1	0	16.3863	Inf	6.61E-06	0.00012264
ATP13A2	0	17.6052	Inf	1.42E-06	3.37E-05
ATP1A2	0	12.2281	Inf	3.34E-06	6.95E-05
ATP1A3	0	21.373	Inf	2.82E-07	8.42E-06
ATP1B2	0	15.9791	Inf	3.34E-05	0.000466589
ATP2A1	0	15.0174	Inf	4.31E-05	0.000573472
ATP2A3	1.99514	17.6483	3.144967331	1.10E-05	0.000187799
ATP2B2	0	10.7591	Inf	4.70E-09	2.48E-07
ATP2B3	0	15.7369	Inf	2.38E-09	1.35E-07
ATP4A	0	17.441	Inf	8.54E-06	0.000151469
ATP8B2	0	9.09582	Inf	2.18E-05	0.000327135
ATP8B3	0	16.0878	Inf	3.34E-08	1.39E-06
ATXN7L1	1.25289	9.75274	2.96054784	6.73E-05	0.000824237
AUTS2	0.695565	11.6287	4.063360674	3.04E-06	6.47E-05

AXIN1	0	14.1331	Inf	6.61E-05	0.000810778
AXL	2.98355	17.3304	2.538203094	8.05E-05	0.00095706
B3GNTL1	0	13.0798	Inf	1.55E-05	0.000248612
B4GALNT1	0	18.3123	Inf	1.42E-06	3.37E-05
B4GALNT3	0	18.6818	Inf	5.57E-06	0.000106758
B4GALNT4	0	14.8512	Inf	7.20E-05	0.000872523
B9D1	0	17.4496	Inf	1.42E-07	4.77E-06
BAHCC1	0.473901	18.3608	5.275899404	5.55E-15	7.66E-13
BAHD1	2.01702	15.9685	2.984931505	5.38E-05	0.000688905
BAIAP2	2.98538	17.341	2.538200612	8.05E-05	0.00095706
BAIAP2-AS	2.20454	19.6956	3.159323808	9.47E-06	0.000165243
BAIAP3	3.83711	20.7045	2.431852334	1.43E-05	0.000232682
BAZ2A	1.06621	9.19557	3.108447387	1.61E-05	0.000257503
BBS5	1.54359	75.2341	5.607025207	1.55E-15	2.44E-13
BCAM	0	17.5892	Inf	1.20E-05	0.000201663
BCAN	0	16.4974	Inf	3.34E-06	6.95E-05
BCDIN3D	0	51.7431	Inf	7.99E-15	1.09E-12
BCL9	0	12.6004	Inf	3.64E-07	1.04E-05
BCORL1	2.6524	14.6052	2.461111849	1.01E-05	0.000174751
BCR	4.22099	19.1665	2.182933594	3.51E-06	7.27E-05
BCRP2	0	46.1718	Inf	1.01E-09	6.23E-08
BCRP3	0	36.9048	Inf	5.57E-05	0.000705723
BDKRB2	0	12.3248	Inf	6.61E-05	0.000810778
BDNF	0	6.80239	Inf	5.57E-05	0.000705723
BEAN1	0	10.1253	Inf	4.70E-05	0.000613083
BEND3	0	11.715	Inf	3.64E-07	1.04E-05
BEND4	0	6.08842	Inf	4.31E-05	0.000573472
BFSP2-AS1	9.9655	58.4737	2.552773786	7.05E-09	3.56E-07
BIN1	2.49265	19.0725	2.935741733	8.42E-05	0.000997309
BMF	0	13.8935	Inf	3.64E-06	7.46E-05
BMP1	0	15.8028	Inf	3.34E-06	6.95E-05
BMP6	0	14.9231	Inf	2.59E-05	0.000377427
BMP7	0	15.3988	Inf	8.54E-06	0.000151469
BMP8A	0	11.1098	Inf	7.84E-06	0.000141315
BRAT1	0	19.1709	Inf	2.00E-05	0.000306049
BRF1	1.80922	15.7637	3.123166444	1.38E-05	0.0002283
BRIP1	1.19352	20.2703	4.086072794	5.85E-12	5.43E-10
BRSK2	0	11.2303	Inf	7.20E-06	0.000131598
BSG	6.11434	36.2372	2.567202801	6.06E-05	0.000757821
BSN	0	18.5022	Inf	1.33E-15	2.44E-13
BTBD11	0	8.53584	Inf	7.84E-05	0.000933973
BTBD9	1.03883	9.5107	3.194591946	6.47E-06	0.000121228
BUB1B-PAK	0	15.2243	Inf	1.55E-05	0.000248612
C10orf105	0	11.969	Inf	1.55E-05	0.000248612
C10orf71	0	14.1077	Inf	7.20E-07	1.87E-05
C11orf21	0	17.1468	Inf	5.57E-05	0.000705723
C11orf72	12.0275	165.97	3.786513785	1.33E-15	2.44E-13
C11orf95	0	14.2863	Inf	2.59E-07	7.83E-06
C12orf65	0	29.5643	Inf	9.30E-06	0.000162965
C14orf132	0	9.16215	Inf	1.20E-06	2.91E-05
C16orf96	0	17.1115	Inf	1.69E-05	0.000266218

C17orf1070		16.3641	Inf	8.53E-05	0.000998703
C17orf51	0	11.1143	Inf	5.12E-08	2.00E-06
C19orf57	0	18.6651	Inf	3.64E-05	0.00050071
C1QTNF5	0	15.7049	Inf	9.30E-06	0.000162965
C1QTNF8	0	19.3635	Inf	3.96E-06	7.98E-05
C1orf106	0	19.8514	Inf	5.58E-08	2.15E-06
C1orf167	2.0861	18.5452	3.152165604	1.02E-05	0.000176179
C1orf226	0	12.6723	Inf	4.31E-05	0.000573472
C1orf229	0	25.5811	Inf	2.00E-05	0.000306049
C20orf2030		12.1413	Inf	1.84E-05	0.000285786
C21orf62	0	17.3637	Inf	1.31E-06	3.13E-05
C2CD4C	0	19.3177	Inf	1.20E-05	0.000201663
C2orf71	0	10.4003	Inf	9.30E-07	2.34E-05
C2orf81	0	21.6396	Inf	4.31E-05	0.000573472
C2orf82	0	17.354	Inf	9.30E-06	0.000162965
C3orf18	0	19.6699	Inf	3.07E-05	0.000432954
C6orf132	2.89121	17.7743	2.620047437	1.28E-06	3.10E-05
C7orf43	0	24.6224	Inf	7.20E-06	0.000131598
C9orf131	0	20.1509	Inf	1.31E-06	3.13E-05
C9orf139	2.55472	22.5982	3.144970779	1.10E-05	0.000187799
CABIN1	3.21404	16.5464	2.364057575	2.56E-06	5.60E-05
CABP4	0	16.7093	Inf	1.10E-06	2.72E-05
CACFD1	0	18.7092	Inf	4.31E-05	0.000573472
CACNA1A	0	12.9668	Inf	3.34E-10	2.27E-08
CACNA1B	0.497766	18.669	5.229033152	1.47E-14	1.91E-12
CACNA1C	0.687858	12.3212	4.162888193	1.08E-12	1.11E-10
CACNA1E	0.323002	7.82862	4.599143014	3.07E-09	1.69E-07
CACNA1F	0	13.5456	Inf	1.42E-07	4.77E-06
CACNA1G	0	17.9304	Inf	3.36E-13	3.73E-11
CACNA1H	0.602513	18.7603	4.960546719	5.05E-12	4.78E-10
CACNA1I	0	15.2565	Inf	1.44E-13	1.66E-11
CACNA1S	0	13.1387	Inf	2.00E-07	6.31E-06
CACNA2D2	0	15.2952	Inf	3.64E-08	1.49E-06
CACNA2D4	0.912058	12.0209	3.720275532	7.39E-05	0.000893921
CACNB1	0	16.1477	Inf	3.64E-06	7.46E-05
CACNB3	0	18.6548	Inf	1.20E-05	0.000201663
CACNG2	0	14.9627	Inf	2.82E-06	6.03E-05
CACNG8	0.556865	15.1716	4.767901811	1.89E-10	1.35E-08
CACTIN	0	25.8041	Inf	1.69E-08	7.60E-07
CAD	0.685005	14.8453	4.437747919	3.28E-08	1.37E-06
CALN1	0.976481	8.50805	3.123164634	1.38E-05	0.0002283
CAMK1D	1.09916	10.5978	3.269291493	2.79E-06	6.03E-05
CAMK2A	0	15.0308	Inf	8.54E-07	2.17E-05
CAMK2B	0	16.0626	Inf	8.54E-07	2.17E-05
CAMK2G	0	11.3079	Inf	2.18E-05	0.000327135
CAMKV	0	19.4639	Inf	5.12E-06	9.95E-05
CAMSAP1	1.27719	10.5068	3.040278275	3.18E-05	0.000447066
CAMTA1	1.04968	8.44942	3.00890273	4.29E-05	0.000573472
CAND2	0	15.4889	Inf	1.42E-06	3.37E-05
CAPN15	0	17.0825	Inf	2.00E-07	6.31E-06
CAPN5	0	15.2093	Inf	3.34E-06	6.95E-05

CARD11	0	16.8855	Inf	5.57E-07	1.50E-05
CARD14	1.98417	15.7962	2.992969996	4.99E-05	0.000648193
CARMIL2	0	14.6285	Inf	4.32E-06	8.63E-05
CARMIL3	0	19.3977	Inf	3.96E-08	1.62E-06
CARNS1	0	17.8115	Inf	1.55E-06	3.62E-05
CASKIN1	0.855685	14.5327	4.086079152	2.39E-06	5.25E-05
CASKIN2	1.88152	16.8097	3.159323447	9.47E-06	0.000165243
CBX2	0	18.2916	Inf	2.59E-08	1.11E-06
CBX7	0	12.7808	Inf	6.07E-05	0.000757821
CC2D1B	0.86189	16.1628	4.229029583	4.77E-07	1.32E-05
CCDC120	2.15054	18.9278	3.137735864	1.19E-05	0.000201663
CCDC136	0	14.721	Inf	5.57E-06	0.000106758
CCDC142	2.2462	22.3527	3.314890811	1.63E-06	3.79E-05
CCDC144B	0	7.5842	Inf	3.96E-06	7.98E-05
CCDC157	0	22.7349	Inf	1.20E-11	1.06E-09
CCDC168	0.453948	4.7784	3.395928676	5.98E-07	1.61E-05
CCDC177	0	13.4094	Inf	2.82E-05	0.000404432
CCDC180	1.30124	11.5678	3.152155533	1.02E-05	0.000176179
CCDC183-A	0	21.8601	Inf	6.07E-05	0.000757821
CCDC185	0	27.9429	Inf	1.69E-05	0.000266218
CCDC33	0	15.7035	Inf	1.20E-05	0.000201663
CCDC40	0	13.3163	Inf	2.38E-08	1.03E-06
CCDC57	0	11.1342	Inf	1.31E-07	4.45E-06
CCDC8	0	25.3413	Inf	1.01E-07	3.58E-06
CCDC85B	0	47.2347	Inf	1.84E-05	0.000285786
CCDC86	0	28.666	Inf	2.00E-05	0.000306049
CCDC88B	1.98095	16.6466	3.070963171	2.35E-05	0.000350829
CCDC88C	3.23023	16.6869	2.369007167	2.38E-06	5.25E-05
CCDC97	2.50933	19.866	2.984927301	5.38E-05	0.000688905
CCL5	11.1428	362.064	5.022061028	1.55E-15	2.44E-13
CD209	0	12.6553	Inf	2.82E-05	0.000404432
CD22	0	20.6699	Inf	2.59E-06	5.60E-05
CD44	4.37837	17.9447	2.035092023	2.54E-05	0.000373958
CD68	2.62561	94.2944	5.16644755	5.95E-14	7.19E-12
CD74	57.9789	173.347	1.580063002	1.02E-08	4.94E-07
CD82	8.52442	56.5531	2.729932527	1.07E-05	0.000184675
CD8A	0	17.1344	Inf	7.20E-06	0.000131598
CD96	0	12.6879	Inf	3.64E-06	7.46E-05
CDAN1	0	12.303	Inf	1.84E-05	0.000285786
CDC42BPG	1.97293	19.3715	3.295523996	2.05E-06	4.60E-05
CDH15	0	19.6005	Inf	2.82E-05	0.000404432
CDH23	0.35521	12.8196	5.17353524	5.06E-14	6.14E-12
CDH24	0	20.6249	Inf	9.30E-07	2.34E-05
CDH4	0.715374	13.9848	4.289018126	2.30E-07	7.11E-06
CDHR1	1.99609	12.1832	2.609644441	3.94E-05	0.000536192
CDHR2	0	19.7996	Inf	6.61E-08	2.47E-06
CDK20	0	22.8364	Inf	3.07E-05	0.000432954
CDK5R2	0	20.9732	Inf	5.12E-05	0.000658307
CDR2L	0	21.6993	Inf	4.70E-07	1.31E-05
CDS2	0.5227	6.98166	3.739515036	6.31E-05	0.000783683
CEACAM5	0	15.0907	Inf	4.70E-05	0.000613083

CEBPA-AS10		28.7896	Inf	6.61E-06	0.00012264
CECR1	0	12.1313	Inf	1.55E-05	0.000248612
CECR2	1.50902	9.69974	2.68433415	1.78E-05	0.000279949
CECR6	0	13.5211	Inf	2.38E-06	5.24E-05
CECR7	0	23.5612	Inf	5.57E-06	0.000106758
CELF3	0	9.27486	Inf	3.96E-05	0.000536192
CELSR1	0	16.01	Inf	1.78E-15	2.69E-13
CELSR2	0.462346	16.768	5.180593905	4.31E-14	5.28E-12
CELSR3	0.814768	13.9098	4.093568547	4.98E-12	4.73E-10
CEMIP	0.670214	11.3827	4.086077175	2.39E-06	5.25E-05
CENPP	0	14.3491	Inf	5.57E-05	0.000705723
CEP164	0.855385	12.9386	3.918963803	1.29E-05	0.000214835
CEP170B	5.72467	19.1793	1.74428563	6.22E-05	0.000775844
CEP250	0.58515	18.0644	4.948199028	6.47E-12	5.95E-10
CERS1	1.29881	17.463	3.749039121	5.83E-05	0.000734196
CES3	1.12804	21.2536	4.235817099	4.40E-07	1.23E-05
CFAP46	0.588684	13.591	4.529014384	8.89E-09	4.38E-07
CFAP65	0	13.5086	Inf	9.31E-11	7.11E-09
CFAP74	0	16.4077	Inf	4.32E-08	1.73E-06
CFLAR	3.3669	14.0248	2.05848742	3.57E-09	1.94E-07
CGREF1	0	23.4862	Inf	6.61E-06	0.00012264
CHADL	0	22.2933	Inf	2.59E-05	0.000377427
CHD5	1.00862	17.353	4.104730455	3.91E-12	3.79E-10
CHGB	0	20.9678	Inf	3.07E-05	0.000432954
CHPF2	0	18.2671	Inf	1.01E-06	2.52E-05
CHRD	0	18.4411	Inf	2.18E-06	4.85E-05
CHRM3	1.53678	20.7079	3.752198703	2.10E-13	2.38E-11
CHRNA2	0	13.4739	Inf	3.07E-05	0.000432954
CHRNA4	0	17.8258	Inf	5.12E-09	2.68E-07
CHST11	0	13.0035	Inf	6.61E-07	1.74E-05
CHST15	0	9.51451	Inf	8.54E-06	0.000151469
CHST3	0	16.0728	Inf	4.32E-10	2.86E-08
CHST6	0	13.5861	Inf	1.69E-08	7.60E-07
CIC	2.33891	19.2927	3.044146808	1.19E-09	7.27E-08
CICP11	0	25.2569	Inf	4.32E-07	1.21E-05
CICP14	4.87963	41.1492	3.076020719	1.16E-07	4.05E-06
CIITA	0	17.873	Inf	1.10E-07	3.84E-06
CILP	0	12.4456	Inf	3.07E-05	0.000432954
CILP2	0	17.7598	Inf	7.20E-07	1.87E-05
CIZ1	1.3852	18.2569	3.72027562	7.39E-05	0.000893921
CLCN2	0	15.3295	Inf	8.53E-05	0.000998703
CLIC6	0	12.4486	Inf	7.84E-05	0.000933973
CLIP2	0	15.4974	Inf	7.20E-08	2.67E-06
CLIP3	0	15.8024	Inf	2.18E-05	0.000327135
CLN8	0	9.25976	Inf	3.64E-06	7.46E-05
CLSPN	0	6.24657	Inf	2.00E-05	0.000306049
CLSTN2	0	10.8604	Inf	5.12E-05	0.000658307
CLSTN3	1.21434	16.1124	3.729927085	6.83E-05	0.00083605
CNGB1	1.53751	13.0562	3.086067363	2.02E-05	0.000308151
CNNM3	0	14.205	Inf	1.84E-06	4.18E-05
CNNM4	0	10.6867	Inf	7.20E-05	0.000872523

CNPPD1	0	21.4059	Inf	2.59E-05	0.000377427
CNPY3	0	16.3128	Inf	7.20E-06	0.000131598
CNTN2	0.602141	17.0443	4.823044185	7.06E-11	5.56E-09
CNTN4	0	9.89214	Inf	4.31E-05	0.000573472
CNTNAP1	0	13.436	Inf	9.30E-07	2.34E-05
COL11A2	0	16.9632	Inf	4.32E-10	2.86E-08
COL16A1	0	17.6778	Inf	6.07E-09	3.12E-07
COL17A1	0	10.6805	Inf	1.31E-05	0.000216387
COL18A1	0	17.7287	Inf	7.85E-11	6.09E-09
COL1A1	0	18.7639	Inf	5.12E-10	3.35E-08
COL20A1	0	19.8378	Inf	1.42E-07	4.77E-06
COL22A1	0	10.3715	Inf	3.96E-06	7.98E-05
COL23A1	0	20.8282	Inf	6.07E-06	0.000114316
COL26A1	0	18.4943	Inf	2.82E-05	0.000404432
COL27A1	0	14.847	Inf	2.01E-10	1.42E-08
COL2A1	0.957957	18.8965	4.302014335	1.96E-07	6.23E-06
COL4A6	0	7.50649	Inf	6.61E-05	0.000810778
COL5A1	1.71811	14.8938	3.115817578	6.37E-08	2.41E-06
COL5A3	0	15.779	Inf	7.84E-09	3.92E-07
COL6A1	0	18.2627	Inf	4.32E-07	1.21E-05
COL6A3	0.455603	8.86623	4.282471636	2.49E-07	7.59E-06
COL6A4P2	0	5.5994	Inf	7.20E-05	0.000872523
COL7A1	0.531479	22.7072	5.416993321	1.55E-15	2.44E-13
COL8A1	0	16.7348	Inf	6.61E-05	0.000810778
COL8A2	0	13.8806	Inf	7.20E-06	0.000131598
COLCA1	0	12.7025	Inf	2.59E-07	7.83E-06
CORO2B	0	12.0314	Inf	5.57E-05	0.000705723
CORO7	0	20.724	Inf	1.10E-07	3.84E-06
CORO7-PAM	0	21.0934	Inf	4.32E-07	1.21E-05
CPAMD8	0	15.8372	Inf	1.31E-08	6.13E-07
CPLX2	0	18.8709	Inf	1.84E-08	8.19E-07
CPT1B	0	17.0676	Inf	4.31E-05	0.000573472
CRAMP1	0	13.5391	Inf	2.18E-09	1.25E-07
CRB2	0	23.3439	Inf	7.23E-13	7.69E-11
CREB3L3	1.86139	34.083	4.194600061	7.14E-07	1.87E-05
CRHR1	0	24.2714	Inf	5.12E-06	9.95E-05
CRHR1-IT1	0	10.5173	Inf	1.31E-05	0.000216387
CRHR1-IT1	0	21.4912	Inf	1.31E-05	0.000216387
CRHR2	0	16.2098	Inf	1.84E-05	0.000285786
CRMP1	0	14.754	Inf	2.82E-06	6.03E-05
CRNN	0	38.9824	Inf	7.20E-07	1.87E-05
CROCC	2.92856	20.4002	2.800319939	8.79E-08	3.17E-06
CROCCP1	1.5624	16.239	3.377627034	7.54E-07	1.96E-05
CROCCP2	2.76098	36.8782	3.739515898	6.31E-05	0.000783683
CRTC1	1.39392	14.6112	3.389855	6.46E-07	1.72E-05
CRX	0	24.982	Inf	4.70E-10	3.09E-08
CRY2	1.11564	15.0003	3.749047883	5.83E-05	0.000734196
CSF1	1.98296	15.6988	2.984926804	5.38E-05	0.000688905
CSF1R	2.38586	18.572	2.960547891	6.73E-05	0.000824237
CSF2RB	0	14.5821	Inf	1.55E-06	3.62E-05
CSF3R	0	23.591	Inf	1.55E-07	5.11E-06

CSMD1	0.680747	6.32273	3.215356989	5.15E-06	0.000100022
CSMD2	0	10.8694	Inf	1.86E-13	2.12E-11
CSPG4	1.17566	15.5993	3.729938492	4.70E-09	2.48E-07
CSPG4P11	2.63983	72.3882	4.777237612	1.55E-15	2.44E-13
CSPG4P13	0	17.1305	Inf	1.01E-07	3.58E-06
CTBP1-AS	2.50804	20.7432	3.04800621	2.95E-05	0.000421596
CTC1	1.37815	12.1906	3.144964308	1.10E-05	0.000187799
CTCFL	0	7.69944	Inf	1.01E-05	0.000174751
CTSD	20.6586	79.1867	1.938515651	9.47E-07	2.39E-05
CUEDC1	2.42143	19.9198	3.04027196	3.18E-05	0.000447066
CUL7	2.61247	16.6385	2.671039002	2.06E-05	0.000313817
CUL9	3.13546	16.3637	2.383749973	1.94E-06	4.39E-05
CUX1	2.50112	10.0388	2.004940643	1.46E-06	3.45E-05
CUX2	0	13.3525	Inf	2.59E-08	1.11E-06
CWC25	0	21.9546	Inf	1.55E-06	3.62E-05
CX3CL1	0	17.4087	Inf	2.00E-05	0.000306049
CXCR5	0	19.1459	Inf	1.42E-05	0.000232345
CXorf36	0.738801	11.6326	3.97684396	7.35E-06	0.000133983
CXorf40A	1.55807	19.6625	3.657614809	2.05E-12	2.04E-10
CYB561	2.85646	23.8776	3.063357602	2.53E-05	0.000372978
CYB561D1	0	14.9621	Inf	4.32E-07	1.21E-05
CYB561D2	0	22.6971	Inf	5.12E-05	0.000658307
CYB5R3	0	20.4683	Inf	1.20E-06	2.91E-05
CYFIP2	0	7.4626	Inf	7.84E-05	0.000933973
CYP11A1	0	22.1921	Inf	4.31E-05	0.000573472
CYP20A1	0.445319	10.163	4.512343354	1.14E-08	5.45E-07
CYP26B1	0	12.9621	Inf	1.55E-05	0.000248612
CYP2S1	0	21.8487	Inf	2.18E-05	0.000327135
CYP4F35P	0	17.8359	Inf	7.20E-05	0.000872523
CYTH3	0	11.3818	Inf	7.84E-05	0.000933973
DAB2IP	4.80743	19.5614	2.024671871	2.90E-05	0.000415005
DACT2	0	15.8215	Inf	3.34E-06	6.95E-05
DAGLA	1.69265	17.7425	3.389853712	6.46E-07	1.72E-05
DBET	2.89808	313.521	6.757320849	1.55E-15	2.44E-13
DCHS1	0.90587	13.9426	3.944051782	1.04E-10	7.84E-09
DCHS2	0.405756	5.70679	3.813995142	3.34E-05	0.000466775
DCLK2	0	13.4247	Inf	1.55E-05	0.000248612
DCUN1D2	0	17.6466	Inf	4.70E-05	0.000613083
DDA1	3.58494	22.1978	2.630395821	3.18E-05	0.000447066
DDR1	1.06493	16.6734	3.968717818	7.96E-06	0.000143202
DDX11L2	4.22831	38.7112	3.194597973	6.47E-06	0.000121228
DDX51	4.13152	22.5671	2.449476479	1.16E-05	0.000196688
DENND1A	0	12.9406	Inf	2.00E-06	4.49E-05
DENND2A	0	19.8188	Inf	6.61E-09	3.35E-07
DENND4B	0.856738	16.3694	4.256003553	3.45E-07	9.99E-06
DERL3	0	17.1077	Inf	3.64E-05	0.00050071
DESI1	0	15.9063	Inf	1.20E-05	0.000201663
DGAT1	1.30963	18.0718	3.786509029	4.24E-05	0.000571998
DGCR14	0	14.9123	Inf	1.42E-07	4.77E-06
DGCR5	0	15.5436	Inf	1.01E-05	0.000174751
DGKD	3.04713	19.3394	2.66602008	6.72E-07	1.77E-05

DGKQ	1.04956	18.2896	4.123166975	1.60E-06	3.72E-05
DGKZ	4.49468	22.1852	2.303307248	5.83E-06	0.000111177
DHX34	0	12.423	Inf	3.96E-05	0.000536192
DHX37	3.21235	19.3224	2.588573305	4.89E-05	0.0006363
DIDO1	0.731207	9.702	3.729930393	4.70E-09	2.48E-07
DIRAS1	0	16.2377	Inf	3.34E-05	0.000466589
DISC1	0	5.68672	Inf	5.57E-07	1.50E-05
DISP2	0	17.0505	Inf	7.84E-08	2.89E-06
DISP3	0	14.1372	Inf	8.54E-07	2.17E-05
DKFZP434A	0	19.02	Inf	2.82E-08	1.19E-06
DLEC1	0	14.4785	Inf	1.84E-07	5.88E-06
DLG5	1.30315	13.8903	3.414002698	4.74E-07	1.32E-05
DLGAP2	0	8.93259	Inf	2.18E-08	9.56E-07
DLGAP3	0	18.7806	Inf	1.10E-06	2.72E-05
DLGAP4	3.21076	17.6798	2.46111522	1.01E-05	0.000174751
DLL1	0	15.4973	Inf	7.20E-05	0.000872523
DLL4	0	17.0155	Inf	1.84E-05	0.000285786
DMBT1	1.26805	9.81466	2.952326658	7.25E-05	0.000878344
DMBX1	0	20.5337	Inf	1.42E-05	0.000232345
DMPK	0	16.6374	Inf	9.30E-06	0.000162965
DMTN	0	13.5574	Inf	3.64E-05	0.00050071
DNAH1	0.743422	16.21	4.446558898	2.44E-15	3.58E-13
DNAH10	0	6.90074	Inf	1.43E-08	6.56E-07
DNAH17	1.06532	11.5594	3.439707759	2.20E-10	1.55E-08
DNAH17-AS	0.943856	14.4438	3.935739768	1.10E-05	0.000187799
DNAH2	0	12.235	Inf	1.33E-15	2.44E-13
DNAH3	0	6.64051	Inf	9.30E-08	3.31E-06
DNAH9	0	8.51605	Inf	1.01E-10	7.68E-09
DNAJB5	0	16.8732	Inf	8.53E-05	0.000998703
DNAJC27-A	0	13.2804	Inf	8.54E-06	0.000151469
DNHD1	0.932299	12.8375	3.783427747	9.64E-14	1.13E-11
DNM1	0	16.0084	Inf	4.70E-06	9.28E-05
DNM1P41	0	31.7613	Inf	1.43E-12	1.45E-10
DNM1P46	0	20.1409	Inf	4.32E-07	1.21E-05
DNMT3A	1.60221	12.5428	2.968724287	6.24E-05	0.000777378
DNPEP	0	13.1817	Inf	2.00E-05	0.000306049
DOCK2	0	8.69614	Inf	5.12E-05	0.000658307
DOCK3	0	7.28691	Inf	6.07E-06	0.000114316
DOCK6	2.28428	13.74	2.588570596	4.89E-05	0.0006363
DOK1	0	21.7463	Inf	1.84E-05	0.000285786
DOK3	0	14.2509	Inf	8.53E-05	0.000998703
DOK7	0	19.8485	Inf	5.12E-07	1.40E-05
DOT1L	1.31069	17.3908	3.72992589	4.70E-09	2.48E-07
DPCR1	0.921893	23.0779	4.645768827	1.47E-09	8.72E-08
DPH7	0	10.2712	Inf	4.70E-05	0.000613083
DPP9	3.42374	20.493	2.581486146	5.25E-05	0.000674957
DPY19L2P2	0	17.8518	Inf	1.55E-07	5.11E-06
DRD2	0	20.6027	Inf	3.07E-05	0.000432954
DRD5P2	0	29.9885	Inf	3.34E-06	6.95E-05
DRP2	0	10.719	Inf	5.12E-07	1.40E-05
DSCAML1	0.70625	16.7426	4.567200812	5.02E-09	2.64E-07

DSG3	1.75577	24.4612	3.800319406	1.43E-09	8.53E-08
DUOX1	0	12.7519	Inf	1.10E-06	2.72E-05
DUOX2	0	14.6041	Inf	2.00E-08	8.81E-07
DUSP15	0	18.284	Inf	2.38E-05	0.000350829
DUSP5P1	0	26.6797	Inf	3.96E-06	7.98E-05
DYSF	0	14.807	Inf	1.01E-09	6.23E-08
ECEL1	0	17.8616	Inf	6.61E-05	0.000810778
EFCC1	0	19.863	Inf	4.70E-05	0.000613083
EFR3B	2.54405	13.7273	2.431848962	1.43E-05	0.000232682
EGFL7	0	30.3705	Inf	3.64E-07	1.04E-05
EGR3	0	14.0688	Inf	6.61E-06	0.00012264
EHD2	0	16.1166	Inf	2.00E-05	0.000306049
ELF4	0	13.622	Inf	1.84E-05	0.000285786
ELFN1	0	13.9386	Inf	6.07E-05	0.000757821
ELFN2	0	15.3004	Inf	5.13E-12	4.80E-10
ELK2BP	0	22.2962	Inf	2.38E-05	0.000350829
ELMO1	0	8.25883	Inf	3.07E-05	0.000432954
ELN	2.43656	20.3676	3.063358337	2.53E-05	0.000372978
EMC3-AS1	0	12.2085	Inf	2.18E-05	0.000327135
EMILIN1	0	22.1113	Inf	6.07E-08	2.30E-06
EMX2OS	0	12.5298	Inf	7.84E-09	3.92E-07
ENC1	0	10.3099	Inf	1.55E-05	0.000248612
ENDOV	0	19.1155	Inf	5.57E-06	0.000106758
ENGASE	0	15.9226	Inf	1.31E-06	3.13E-05
ENPP7P13	0	10.7805	Inf	1.69E-05	0.000266218
ENSG0000C	0	11.5413	Inf	7.20E-06	0.000131598
ENSG0000C	0	30.5024	Inf	9.30E-08	3.31E-06
ENSG0000C	0	16.3193	Inf	8.54E-08	3.08E-06
ENSG0000C	0	21.6467	Inf	3.64E-05	0.00050071
ENSG0000C	0	26.1504	Inf	1.69E-08	7.60E-07
ENSG0000C	0	20.2578	Inf	5.57E-05	0.000705723
ENSG0000C	5.15402	24.467	2.247067177	1.37E-06	3.28E-05
ENSG0000C	0.310489	7.77254	4.645772073	1.47E-09	8.72E-08
ENSG0000C	0.455965	6.85663	3.910504681	1.97E-10	1.40E-08
ENSG0000C	0	20.5118	Inf	2.59E-05	0.000377427
ENSG0000C	0	16.4111	Inf	2.82E-07	8.42E-06
ENSG0000C	2.15912	172.08	6.316492192	1.55E-15	2.44E-13
ENSG0000C	0	19.4524	Inf	5.60E-13	6.09E-11
ENSG0000C	0	30.6831	Inf	4.32E-11	3.56E-09
ENSG0000C	2.08431	20.9261	3.32766169	1.40E-06	3.34E-05
ENSG0000C	0	4.36997	Inf	8.54E-08	3.08E-06
ENSG0000C	0	15.4128	Inf	4.70E-05	0.000613083
ENSG0000C	0	15.8657	Inf	2.82E-05	0.000404432
ENSG0000C	0	36.0468	Inf	3.07E-10	2.10E-08
ENSG0000C	0	17.1628	Inf	1.42E-05	0.000232345
ENSG0000C	0	15.6017	Inf	4.70E-05	0.000613083
ENSG0000C	0	42.5047	Inf	4.70E-08	1.85E-06
ENSG0000C	0	19.1639	Inf	5.12E-06	9.95E-05
ENSG0000C	0	15.4111	Inf	1.55E-05	0.000248612
ENSG0000C	0	9.45665	Inf	1.31E-05	0.000216387
ENSG0000C	0	33.5422	Inf	1.10E-07	3.84E-06

ENSG0000C	0	11.0919	Inf	2.59E-05	0.000377427
ENSG0000C	1.61469	15.0685	3.222206693	4.77E-06	9.41E-05
ENSG0000C	0	35.9409	Inf	3.07E-06	6.49E-05
ENSG0000C	0	16.3396	Inf	1.31E-05	0.000216387
ENSG0000C	26.4485	414.098	3.968714426	1.55E-15	2.44E-13
ENSG0000C	0	16.1959	Inf	3.07E-06	6.49E-05
ENSG0000C	0.300532	8.04167	4.741904615	1.55E-15	2.44E-13
ENSG0000C	0	15.2674	Inf	1.10E-05	0.000187799
ENSG0000C	0	17.7757	Inf	1.42E-07	4.77E-06
ENSG0000C	0	11.4629	Inf	3.07E-05	0.000432954
ENSG0000C	0	20.1402	Inf	6.61E-08	2.47E-06
ENSG0000C	2.40966	16.5546	2.780330649	5.31E-11	4.29E-09
ENSG0000C	0	23.1961	Inf	2.38E-05	0.000350829
ENSG0000C	50.7324	538.763	3.408671459	1.33E-15	2.44E-13
ENSG0000C	0	32.9376	Inf	1.10E-07	3.84E-06
ENSG0000C	0	16.8998	Inf	6.07E-06	0.000114316
ENSG0000C	0	13.0919	Inf	6.07E-05	0.000757821
ENSG0000C	0	9.9898	Inf	6.61E-05	0.000810778
ENSG0000C	0	54.1388	Inf	3.07E-10	2.10E-08
ENSG0000C	0	84.0867	Inf	6.22E-14	7.44E-12
ENSG0000C	0	35.0609	Inf	2.18E-09	1.25E-07
ENSG0000C	0	9.55664	Inf	1.84E-07	5.88E-06
ENSG0000C	0.731701	29.6435	5.340317756	2.22E-15	3.29E-13
ENSG0000C	0	19.87	Inf	2.00E-05	0.000306049
ENSG0000C	0	24.3036	Inf	6.07E-06	0.000114316
ENSG0000C	0	9.9506	Inf	4.31E-05	0.000573472
ENSG0000C	0	111.13	Inf	1.32E-13	1.53E-11
ENSG0000C	2.46366	250.18	6.666019482	1.55E-15	2.44E-13
ENSG0000C	0	9.24315	Inf	7.84E-05	0.000933973
ENSG0000C	0	19.9964	Inf	1.20E-06	2.91E-05
ENSG0000C	7.13071	160.843	4.495463608	1.33E-15	2.44E-13
ENSG0000C	1.94691	22.9049	3.556398165	1.98E-11	1.71E-09
ENSG0000C	0	28.6457	Inf	4.31E-05	0.000573472
ENSG0000C	3.42094	28.2935	3.048005948	2.95E-05	0.000421596
ENSG0000C	0	10.5208	Inf	7.85E-10	4.95E-08
ENSG0000C	0	18.3188	Inf	7.84E-06	0.000141315
ENSG0000C	55.1229	1579.82	4.840964591	1.55E-15	2.44E-13
ENSG0000C	0	16.0546	Inf	2.18E-05	0.000327135
ENSG0000C	11.4259	653.917	5.838727816	1.33E-15	2.44E-13
ENSG0000C	0	19.8375	Inf	5.57E-05	0.000705723
ENSG0000C	0	83.5421	Inf	1.20E-07	4.12E-06
ENSG0000C	1.23121	22.653	4.201553363	6.58E-07	1.74E-05
ENSG0000C	0	16.6112	Inf	6.61E-05	0.000810778
ENSG0000C	0	19.3328	Inf	3.34E-05	0.000466589
ENSG0000C	1.42198	11.0061	2.952330263	7.25E-05	0.000878344
ENSG0000C	0	9.67145	Inf	1.84E-05	0.000285786
ENSG0000C	0	19.4243	Inf	1.43E-08	6.56E-07
ENSG0000C	0	236.829	Inf	1.33E-15	2.44E-13
ENSG0000C	12.3829	301.388	4.605200846	1.33E-15	2.44E-13
ENSG0000C	0	98.6544	Inf	1.82E-14	2.32E-12
ENSG0000C	0	26.0459	Inf	6.61E-05	0.000810778

ENSG0000C	2.04538	19.1783	3.229034039	4.42E-06	8.82E-05
ENSG0000C	0	15.2738	Inf	8.54E-06	0.000151469
ENSG0000C	15.6692	602.236	5.264325522	1.55E-15	2.44E-13
ENSG0000C	0	8.28358	Inf	2.38E-05	0.000350829
ENSG0000C	0	26.2152	Inf	1.20E-06	2.91E-05
ENSG0000C	0	21.5425	Inf	4.32E-08	1.73E-06
ENSG0000C	0	15.9444	Inf	7.20E-09	3.62E-07
ENSG0000C	0	19.9998	Inf	1.10E-05	0.000187799
ENSG0000C	0	22.4801	Inf	1.31E-10	9.69E-09
ENSG0000C	0	12.5576	Inf	5.12E-06	9.95E-05
ENSG0000C	1.57325	15.6559	3.314886598	1.63E-06	3.79E-05
ENSG0000C	0	37.5694	Inf	1.33E-15	2.44E-13
ENSG0000C	0	16.6504	Inf	1.84E-06	4.18E-05
ENSG0000C	0	27.9448	Inf	3.64E-07	1.04E-05
ENSG0000C	0	16.6308	Inf	4.70E-05	0.000613083
ENSG0000C	8.62501	205.23	4.572571696	4.63E-09	2.46E-07
ENSG0000C	4.02239	104.607	4.700782617	1.55E-15	2.44E-13
ENSG0000C	0	14.31	Inf	5.57E-05	0.000705723
ENSG0000C	84.0195	4921.62	5.872265261	1.11E-15	2.44E-13
ENSG0000C	0	13.2245	Inf	1.55E-10	1.13E-08
ENSG0000C	52.2121	3542.39	6.084195058	1.33E-15	2.44E-13
ENSG0000C	0	34.046	Inf	3.96E-07	1.12E-05
ENSG0000C	0	19.5592	Inf	3.07E-05	0.000432954
ENSG0000C	0	12.9556	Inf	1.69E-05	0.000266218
ENSG0000C	0	23.4539	Inf	4.70E-05	0.000613083
ENSG0000C	59.5605	3049.51	5.678077771	1.11E-15	2.44E-13
ENSG0000C	0	283.965	Inf	7.99E-14	9.46E-12
ENSG0000C	0	15.206	Inf	3.64E-07	1.04E-05
ENSG0000C	0	15.5667	Inf	1.69E-05	0.000266218
ENSG0000C	0	102.257	Inf	1.43E-09	8.49E-08
ENSG0000C	0	16.3646	Inf	7.20E-07	1.87E-05
ENSG0000C	0	19.9351	Inf	3.96E-08	1.62E-06
ENSG0000C	0	95.7342	Inf	2.18E-07	6.76E-06
ENSG0000C	1.36963	11.8124	3.108444003	1.61E-05	0.000257503
ENSG0000C	0	27.0184	Inf	1.43E-08	6.56E-07
ENSG0000C	0	14.3458	Inf	1.20E-06	2.91E-05
ENSG0000C	6.93931	31.5354	2.18410811	6.26E-08	2.37E-06
ENSG0000C	0	252.819	Inf	5.14E-13	5.64E-11
ENSG0000C	0	14.3824	Inf	1.20E-05	0.000201663
ENSG0000C	0	1108.33	Inf	1.33E-15	2.44E-13
ENSG0000C	1.90023	22.8599	3.588573137	4.24E-08	1.72E-06
ENSG0000C	0	36.7005	Inf	2.00E-06	4.49E-05
ENSG0000C	0	10.3045	Inf	9.30E-06	0.000162965
ENSG0000C	0	35.5344	Inf	2.82E-05	0.000404432
ENSG0000C	0	53.8596	Inf	1.33E-15	2.44E-13
ENSG0000C	0	120.872	Inf	1.31E-06	3.13E-05
ENSG0000C	0	20.533	Inf	3.34E-06	6.95E-05
ENSG0000C	0	116.824	Inf	1.33E-15	2.44E-13
ENSG0000C	0	25.2626	Inf	1.69E-07	5.47E-06
ENSG0000C	29.4153	1389.74	5.562104441	1.33E-15	2.44E-13
ENSG0000C	0	12.3249	Inf	9.30E-08	3.31E-06

ENSG0000C0	165.54	Inf	6.07E-08	2.30E-06
ENSG0000C0	61.8848	Inf	6.61E-07	1.74E-05
ENSG0000C0	19.318	Inf	1.55E-05	0.000248612
ENSG0000C0	20.8547	Inf	1.55E-08	7.06E-07
ENSG0000C0	8.68594	Inf	2.00E-06	4.49E-05
ENSG0000C0	79.5715	Inf	9.31E-11	7.11E-09
ENSG0000C0	11.8223	Inf	9.30E-06	0.000162965
ENSG0000C0	7.13302	Inf	1.31E-05	0.000216387
ENSG0000C0	16.4658	Inf	2.00E-05	0.000306049
ENSG0000C0	17.5055	Inf	1.42E-06	3.37E-05
ENSG0000C0	21.4512	Inf	2.59E-06	5.60E-05
ENSG0000C0	26.7574	Inf	1.55E-07	5.11E-06
ENSG0000C0	18.6351	Inf	1.69E-07	5.47E-06
ENSG0000C1.11411	16.1623	3.85866893	2.25E-05	0.000336458
ENSG0000C0	16.9995	Inf	3.64E-06	7.46E-05
ENSG0000C0	68.8989	Inf	3.97E-09	2.13E-07
ENSG0000C0	12.5137	Inf	5.12E-05	0.000658307
ENSG0000C0	165.792	Inf	2.00E-08	8.81E-07
ENSG0000C1.84031	20.8367	3.501106096	1.48E-07	4.94E-06
ENSG0000C0	23.8769	Inf	2.59E-05	0.000377427
ENSG0000C0	23.2936	Inf	3.34E-07	9.75E-06
ENSG0000C0	11.1971	Inf	1.31E-07	4.45E-06
ENSG0000C1.22718	196.805	7.325276188	1.55E-15	2.44E-13
ENSG0000C1.08557	17.2846	3.992962555	6.26E-06	0.000117596
ENSG0000C0	18.0851	Inf	5.12E-11	4.15E-09
ENSG0000C0	13.1803	Inf	3.07E-07	9.07E-06
ENSG0000C0	24.5003	Inf	6.07E-08	2.30E-06
ENSG0000C0	17.3598	Inf	1.69E-05	0.000266218
ENSG0000C0	18.0334	Inf	5.57E-07	1.50E-05
ENSG0000C0	13.7136	Inf	3.64E-05	0.00050071
ENSG0000C0	31.0363	Inf	1.84E-07	5.88E-06
ENSG0000C0	38.0006	Inf	2.59E-05	0.000377427
ENSG0000C0	22.577	Inf	1.20E-05	0.000201663
ENSG0000C0	12.203	Inf	7.84E-06	0.000141315
ENSG0000C0	16.7563	Inf	2.59E-05	0.000377427
ENSG0000C0	23.8814	Inf	1.20E-05	0.000201663
ENSG0000C1.96695	15.5721	2.984931325	5.38E-05	0.000688905
ENSG0000C0	9.51489	Inf	1.84E-05	0.000285786
ENSG0000C58.3608	488.492	3.065263371	1.33E-15	2.44E-13
ENSG0000C0	23.3988	Inf	4.32E-08	1.73E-06
ENSG0000C0	34.3372	Inf	6.07E-05	0.000757821
ENSG0000C0	24.4591	Inf	1.20E-07	4.12E-06
ENSG0000C0	8.77266	Inf	8.53E-05	0.000998703
ENSG0000C1.82241	30.3064	4.055703015	3.30E-06	6.92E-05
ENSG0000C0	30.9513	Inf	1.20E-12	1.23E-10
ENSG0000C0	28.2023	Inf	5.12E-05	0.000658307
ENSG0000C0	17.8594	Inf	2.38E-05	0.000350829
ENSG0000C0	13.09	Inf	2.18E-07	6.76E-06
ENSG0000C0	50.7858	Inf	1.55E-06	3.62E-05
ENSG0000C2.64556	51.6008	4.28574839	6.06E-14	7.30E-12
ENSG0000C41.8771	1614.93	5.269166283	1.78E-15	2.69E-13

ENSG0000C	0	40.8764	Inf	2.38E-05	0.000350829
ENSG0000C	0	14.6708	Inf	2.82E-06	6.03E-05
ENSG0000C	0	327.848	Inf	1.98E-14	2.51E-12
ENSG0000C	0	8.85101	Inf	8.54E-07	2.17E-05
ENSG0000C	0	9.97473	Inf	2.59E-06	5.60E-05
ENSG0000C	0	16.1189	Inf	6.07E-08	2.30E-06
ENSG0000C	0	103.726	Inf	5.57E-05	0.000705723
ENSG0000C	0	13.1879	Inf	6.07E-05	0.000757821
ENSG0000C	0	30.6711	Inf	5.12E-10	3.35E-08
ENSG0000C	0	13.9815	Inf	4.70E-05	0.000613083
ENSG0000C	0	257.939	Inf	1.10E-08	5.30E-07
ENSG0000C	0	14.7168	Inf	4.70E-05	0.000613083
ENSG0000C	0	10.6867	Inf	3.34E-05	0.000466589
ENSG0000C	2.46024	14.1456	2.523482407	4.63E-06	9.22E-05
ENSG0000C	0	16.3181	Inf	5.57E-06	0.000106758
ENSG0000C	3.19969	52.9273	4.048008017	3.57E-06	7.37E-05
ENSG0000C	5.1513	35.5419	2.78651234	5.56E-06	0.000106758
ENSG0000C	0	16.7933	Inf	4.70E-07	1.31E-05
ENSG0000C	1.69559	20.2481	3.57792727	4.96E-08	1.94E-06
ENSG0000C	0	17.3218	Inf	2.82E-06	6.03E-05
ENSG0000C	0	14.4385	Inf	2.38E-07	7.25E-06
ENSG0000C	0	41.0314	Inf	4.70E-07	1.31E-05
ENSG0000C	1.12414	18.2964	4.024666187	4.54E-06	9.06E-05
ENSG0000C	0.72109	16.5841	4.523477576	9.65E-09	4.71E-07
ENSG0000C	0	17.2367	Inf	6.07E-05	0.000757821
ENSG0000C	0	6.3949	Inf	7.84E-05	0.000933973
ENSG0000C	0	9.2885	Inf	4.32E-06	8.63E-05
ENSG0000C	0	13.1645	Inf	4.31E-05	0.000573472
ENTPD4	2.47912	23.0259	3.215357536	1.29E-08	6.10E-07
ENTPD6	3.01804	18.8655	2.644066505	2.75E-05	0.000399206
EP300-AS1	0	36.9122	Inf	7.84E-05	0.000933973
EP400	1.18693	8.88928	2.904831717	1.27E-06	3.08E-05
EPHA10	0.733463	21.021	4.840963302	5.08E-11	4.13E-09
EPHA8	0	13.918	Inf	6.07E-07	1.62E-05
EPHB2	0	10.5041	Inf	2.01E-10	1.42E-08
EPHB3	0	17.2628	Inf	1.01E-06	2.52E-05
EPHB6	0	19.8591	Inf	2.18E-07	6.76E-06
EPN3	0	16.0262	Inf	7.20E-06	0.000131598
EPOP	0	19.7391	Inf	6.07E-06	0.000114316
EPPK1	2.43687	18.3223	2.910499422	1.40E-08	6.52E-07
EPS15L1	0	11.3789	Inf	1.69E-05	0.000266218
ERBB2	1.77301	14.5856	3.040272156	3.18E-05	0.000447066
ERCC6L2	0	5.36661	Inf	3.34E-06	6.95E-05
ERN2	0	17.9757	Inf	4.70E-06	9.28E-05
ERVK13-1	0.584588	10.3421	4.144965135	1.26E-06	3.04E-05
ESPL1	1.47158	15.0347	3.352858202	1.03E-06	2.55E-05
ESPN	0	15.7482	Inf	3.07E-05	0.000432954
ESPNL	0	19.2198	Inf	2.00E-08	8.81E-07
ESPNP	0	32.0888	Inf	5.12E-06	9.95E-05
ESRG	1.55294	34.0672	4.455309381	2.57E-08	1.11E-06
EVC	0.731591	13.7194	4.229036251	4.77E-07	1.32E-05

EVC2	0	11.2597	Inf	2.00E-05	0.000306049
EVI5L	0	21.9677	Inf	1.01E-07	3.58E-06
EVPL	3.64755	21.553	2.562889035	1.26E-07	4.30E-06
EVX1	0	21.7169	Inf	2.38E-06	5.24E-05
EXD3	0	16.988	Inf	1.10E-07	3.84E-06
EXOC7	2.70679	16.7603	2.630393104	3.18E-05	0.000447066
EXTL1	1.21373	18.5737	3.93574181	1.10E-05	0.000187799
EXTL3	1.53726	12.0343	2.96871913	6.24E-05	0.000777378
F2RL3	0	17.5308	Inf	1.10E-05	0.000187799
F7	0	22.9144	Inf	9.30E-07	2.34E-05
FAAP20	0.696858	17.198	4.625232284	2.04E-09	1.18E-07
FAIM2	2.05444	22.1709	3.431850234	3.76E-07	1.07E-05
FAM107A	0	13.8173	Inf	1.01E-05	0.000174751
FAM131B	0	18.1468	Inf	2.18E-07	6.76E-06
FAM155B	0	14.265	Inf	2.18E-05	0.000327135
FAM163A	0	12.1557	Inf	2.82E-05	0.000404432
FAM178B	0	19.2092	Inf	2.00E-05	0.000306049
FAM179A	0	16.8876	Inf	1.31E-05	0.000216387
FAM184B	0	13.0828	Inf	8.53E-05	0.000998703
FAM189A1	0	11.9968	Inf	2.59E-05	0.000377427
FAM193B	0	14.9281	Inf	2.00E-06	4.49E-05
FAM212B	0	12.0586	Inf	9.30E-07	2.34E-05
FAM219A	0	19.6313	Inf	1.31E-06	3.13E-05
FAM234A	0	15.0486	Inf	1.84E-05	0.000285786
FAM27E2	0	30.0059	Inf	5.12E-05	0.000658307
FAM27E3	0	60.0681	Inf	3.07E-08	1.29E-06
FAM30A	0	18.9231	Inf	1.78E-15	2.69E-13
FAM43A	0	18.9146	Inf	1.42E-05	0.000232345
FAM43B	0	21.7033	Inf	2.82E-05	0.000404432
FAM53B	0.848977	11.7903	3.795731148	3.92E-05	0.000536192
FAM65A	0	15.9723	Inf	1.31E-06	3.13E-05
FAM65C	0	13.63	Inf	3.34E-06	6.95E-05
FAM78B	0	7.84194	Inf	3.64E-05	0.00050071
FAM84A	0	6.88243	Inf	8.54E-06	0.000151469
FANCA	2.36368	14.4267	2.609634693	3.94E-05	0.000536192
FANCG	0	22.2821	Inf	1.69E-05	0.000266218
FAT2	0	9.19294	Inf	6.08E-12	5.62E-10
FAT4	0	5.26922	Inf	9.30E-08	3.31E-06
FBF1	3.12447	18.8859	2.595626143	4.55E-05	0.000603937
FBLIM1	2.58316	33.3606	3.690936325	8.82E-09	4.35E-07
FBLN2	0	15.6161	Inf	1.01E-06	2.52E-05
FBN3	0.513772	16.1789	4.976841445	3.63E-12	3.55E-10
FBXL18	0	17.068	Inf	4.46E-14	5.43E-12
FBXL19-AS	0	18.2199	Inf	1.20E-06	2.91E-05
FBXL20	1.87771	9.79965	2.383755958	2.49E-05	0.000367051
FBXO31	1.59592	14.6816	3.201548962	6.00E-06	0.000113855
FBXO41	0	14.4973	Inf	4.32E-09	2.31E-07
FBXO44	0	16.1327	Inf	2.38E-05	0.000350829
FCER1G	0	86.0661	Inf	7.84E-05	0.000933973
FCGBP	0	8.86316	Inf	6.10E-13	6.55E-11
FCGR2A	0	21.6506	Inf	5.57E-05	0.000705723

FCHO1	0	16.8484	Inf	2.82E-05	0.000404432
FCHSD1	1.12804	16.1647	3.840956601	2.63E-05	0.000383981
FCN1	0	10.1095	Inf	3.96E-07	1.12E-05
FDPSP2	0	15.3177	Inf	5.12E-07	1.40E-05
FENDRR	0	9.67196	Inf	4.31E-05	0.000573472
FER1L4	0	15.09	Inf	3.64E-09	1.98E-07
FER1L5	0.753538	15.464	4.359089465	9.43E-08	3.35E-06
FERMT3	0	27.5324	Inf	1.69E-06	3.89E-05
FGD1	0	18.8557	Inf	2.18E-07	6.76E-06
FGD2	0	18.8279	Inf	2.18E-05	0.000327135
FGD3	0	21.3612	Inf	4.32E-08	1.73E-06
FGD5	0	11.8419	Inf	1.84E-06	4.18E-05
FGF5	0	15.9764	Inf	2.82E-08	1.19E-06
FGR	0	18.6471	Inf	6.61E-05	0.000810778
FHAD1	0	10.5013	Inf	4.70E-05	0.000613083
FHDC1	0	9.57911	Inf	8.54E-06	0.000151469
FIBCD1	0	16.215	Inf	4.31E-05	0.000573472
FITM2	0	14.0002	Inf	4.32E-06	8.63E-05
FJX1	0	20.053	Inf	4.31E-05	0.000573472
FLCN	2.4047	18.3995	2.935737736	8.42E-05	0.000997309
FLG	2.29378	24.6523	3.425923321	1.33E-15	2.44E-13
FLG2	1.06843	12.4753	3.545510214	7.93E-08	2.91E-06
FLJ16779	1.27602	12.5853	3.30201676	1.90E-06	4.31E-05
FLJ36000	0	23.3803	Inf	4.32E-08	1.73E-06
FLJ42627	0	18.4533	Inf	8.55E-12	7.74E-10
FLNC	0	20.8501	Inf	1.55E-15	2.44E-13
FLT4	2.28821	15.1805	2.729927943	1.07E-05	0.000184675
FMN1	2.04901	7.94903	1.955851797	1.44E-05	0.000235016
FMN2	0	8.43247	Inf	3.96E-05	0.000536192
FMNL1	0	18.1648	Inf	1.20E-06	2.91E-05
FMNL3	0.435412	12.5174	4.845409951	4.68E-11	3.83E-09
FNDC1	0	14.7393	Inf	9.30E-09	4.55E-07
FOXD2	1.04238	14.0152	3.749039118	5.83E-05	0.000734196
FOXJ1	0	24.4828	Inf	5.12E-06	9.95E-05
FOXK1	6.09631	200.103	5.03666272	1.11E-15	2.44E-13
FOXP4	2.45745	17.7525	2.852788238	2.48E-06	5.44E-05
FRAS1	0.589076	6.8782	3.545505385	7.93E-08	2.91E-06
FREM2	0	6.02732	Inf	7.84E-09	3.92E-07
FREM3	0.735678	9.63117	3.710564714	8.00E-05	0.000951668
FRMD8	2.59762	24.3563	3.229032685	4.42E-06	8.82E-05
FRMPD1	0	11.9252	Inf	1.69E-05	0.000266218
FRMPD3	0	14.4001	Inf	1.69E-09	9.89E-08
FRMPD4	0	9.11515	Inf	4.32E-07	1.21E-05
FSTL4	0	10.7324	Inf	2.00E-05	0.000306049
FTL	441.994	951.697	1.106475538	4.13E-14	5.08E-12
FUT1	0	13.1575	Inf	2.82E-05	0.000404432
FUT4	0.805742	10.9761	3.767903761	4.97E-05	0.000647357
FUT6	0	18.2757	Inf	2.18E-05	0.000327135
FYCO1	2.28839	12.8033	2.484110857	7.59E-06	0.000138112
FZD3	0	3.65808	Inf	8.53E-05	0.000998703
FZD5	0	8.93116	Inf	1.69E-05	0.000266218

FZR1	2.68936	20.5776	2.935739934	8.42E-05	0.000997309
GAA	3.14125	19.7284	2.650863285	2.56E-05	0.000376121
GAB2	0	13.2719	Inf	1.42E-07	4.77E-06
GABBR1	0	11.3952	Inf	2.00E-05	0.000306049
GABBR2	0	10.8733	Inf	7.20E-06	0.000131598
GALNT16	0	12.2045	Inf	5.57E-05	0.000705723
GALNT6	0	14.3051	Inf	5.12E-06	9.95E-05
GALNT9	0	18.6005	Inf	3.96E-05	0.000536192
GAMT	0	26.1365	Inf	1.69E-05	0.000266218
GAREM2	1.64106	17.9276	3.449482473	2.98E-07	8.87E-06
GAS2L1P2	0	24.7736	Inf	2.82E-05	0.000404432
GAS6	0	24.9964	Inf	6.61E-06	0.00012264
GAS7	2.19609	12.0926	2.461115383	1.01E-05	0.000174751
GATA2	0	18.3235	Inf	2.18E-06	4.85E-05
GATA4	0	17.0959	Inf	3.64E-06	7.46E-05
GATS	0	10.1131	Inf	1.10E-05	0.000187799
GATSL2	0	17.6376	Inf	3.65E-12	3.56E-10
GBP4	3.17209	33.18	3.386808277	5.92E-13	6.38E-11
GBP5	0	15.1062	Inf	1.01E-05	0.000174751
GCK	0	22.8646	Inf	1.69E-06	3.89E-05
GDF5	0	22.2761	Inf	2.00E-05	0.000306049
GDF7	0	8.62385	Inf	1.10E-07	3.84E-06
GDPD5	0	19.0134	Inf	1.20E-05	0.000201663
GEMIN8	0	27.5116	Inf	3.64E-08	1.49E-06
GFAP	0	16.8834	Inf	2.59E-06	5.60E-05
GFOD2	0	12.0793	Inf	1.55E-07	5.11E-06
GGT5	0	27.9714	Inf	1.10E-06	2.72E-05
GGT8P	0	36.9629	Inf	2.38E-08	1.03E-06
GIGYF1	0.769968	18.185	4.561806627	5.45E-09	2.84E-07
GIPR	0	19.0039	Inf	7.84E-06	0.000141315
GJB3	0	22.5427	Inf	5.12E-05	0.000658307
GJD3	0	20.6892	Inf	1.20E-07	4.12E-06
GLI1	0	14.9955	Inf	3.96E-05	0.000536192
GLI2	0	12.6089	Inf	8.54E-08	3.08E-06
GLI3	0	7.13623	Inf	1.69E-05	0.000266218
GLIS1	0	20.359	Inf	2.18E-05	0.000327135
GLIS2	0	16.4939	Inf	8.54E-07	2.17E-05
GLP1R	0	8.83795	Inf	1.55E-05	0.000248612
GLUL	9.10759	25.0751	1.461114203	3.73E-06	7.64E-05
GLYCTK	2.5648	20.1918	2.97685129	5.79E-05	0.00073122
GNAO1	0	11.5885	Inf	2.38E-08	1.03E-06
GNL3L	1.68232	9.87122	2.552776249	6.99E-05	0.000853216
GOLGA2P7	2.90413	29.4138	3.340315239	1.20E-06	2.91E-05
GOLGA6L1	0	28.6471	Inf	7.21E-10	4.58E-08
GOLGA6L2	0	19.5625	Inf	9.30E-06	0.000162965
GOLGA6L6	1.21434	31.0432	4.676032961	9.00E-10	5.59E-08
GOLGA7B	0	10.6294	Inf	2.38E-06	5.24E-05
GORASP1	0	16.8686	Inf	6.07E-06	0.000114316
GOSR1	6.51363	47.9047	2.878633531	6.99E-14	8.31E-12
GPC1	2.64413	21.5179	3.024672273	3.69E-05	0.000508002
GPR153	0	17.5296	Inf	1.31E-06	3.13E-05

GPR158	0	7.82804	Inf	6.07E-05	0.000757821
GPR161	2.14321	11.1379	2.377632122	2.67E-05	0.000388676
GPR179	0.610668	18.7441	4.939904498	7.63E-12	6.94E-10
GPR26	0	9.32722	Inf	1.01E-08	4.93E-07
GPR37L1	0	26.6454	Inf	4.32E-06	8.63E-05
GPR78	0	15.9084	Inf	1.31E-06	3.13E-05
GPRC5A	0	18.8995	Inf	4.31E-05	0.000573472
GPRIN1	0	19.873	Inf	1.20E-07	4.12E-06
GPRIN2	0	29.7935	Inf	3.96E-05	0.000536192
GPSM1	0	18.7065	Inf	3.64E-08	1.49E-06
GREB1	1.88516	18.1762	3.269291734	1.01E-11	9.05E-10
GRID1	0	11.4969	Inf	3.07E-06	6.49E-05
GRID2IP	1.05206	15.9135	3.918962285	1.29E-05	0.000214835
GRIK3	0.528596	14.2144	4.749043885	2.63E-10	1.83E-08
GRIK4	0	9.02743	Inf	2.38E-05	0.000350829
GRIK5	0	14.9228	Inf	5.57E-06	0.000106758
GRIN1	0	17.5527	Inf	3.64E-07	1.04E-05
GRIN2A	0	5.85861	Inf	6.61E-08	2.47E-06
GRIN2B	1.64051	14.6565	3.159324333	9.47E-06	0.000165243
GRIN2C	0	20.5476	Inf	3.97E-09	2.13E-07
GRIN2D	0	11.5107	Inf	1.69E-05	0.000266218
GRIP2	0.631398	12.6782	4.327656431	1.41E-07	4.77E-06
GRM1	0	8.50901	Inf	1.84E-05	0.000285786
GRM4	2.3222	19.1548	3.044141827	1.19E-09	7.27E-08
GRM6	0.793281	12.4904	3.976843874	7.35E-06	0.000133983
GSE1	0.642384	11.1941	4.123158771	1.60E-06	3.72E-05
GSN-AS1	0	17.5573	Inf	1.20E-07	4.12E-06
GTF3C1	2.05704	12.5551	2.609631767	3.94E-05	0.000536192
GTPBP2	0	16.2638	Inf	8.53E-05	0.000998703
GUSBP11	0	15.1382	Inf	6.61E-05	0.000810778
H6PD	2.09777	12.7574	2.604405919	1.59E-06	3.71E-05
HAGLR	0	11.4703	Inf	4.70E-05	0.000613083
HAP1	2.46242	19.7124	3.000954677	4.63E-05	0.000611824
HAUS5	0	12.4046	Inf	4.70E-05	0.000613083
HCFC1	4.04841	16.3707	2.015688701	6.26E-06	0.000117604
HCG18	1.09582	9.49941	3.115827072	1.49E-05	0.000242721
HCN3	0	17.0243	Inf	3.96E-06	7.98E-05
HCN4	0	13.2992	Inf	1.01E-08	4.93E-07
HDAC10	0	22.9304	Inf	8.54E-06	0.000151469
HDAC4	1.62872	11.9099	2.870350789	1.98E-06	4.49E-05
HECTD4	1.57706	8.25849	2.388640471	1.81E-06	4.15E-05
HEG1	0	6.07326	Inf	3.07E-05	0.000432954
HELZ2	0.962213	14.4268	3.906251231	2.13E-10	1.51E-08
HEMK1	0.787513	15.0467	4.255999564	3.45E-07	9.99E-06
HES2	0	16.587	Inf	1.55E-06	3.62E-05
HEYL	0	12.4687	Inf	7.20E-05	0.000872523
HHIPL1	1.56157	14.3196	3.196922046	1.75E-08	7.88E-07
HIC1	0	16.3301	Inf	6.07E-05	0.000757821
HIC2	0	16.2329	Inf	6.08E-10	3.92E-08
HIF1AN	1.41005	12.0363	3.093573745	1.87E-05	0.000290534
HIF3A	0	11.9327	Inf	5.12E-07	1.40E-05

HIVEP3	0	12.9791	Inf	1.78E-15	2.69E-13
HK2P1	0	10.3824	Inf	1.84E-05	0.000285786
HLCS	0	8.36612	Inf	9.30E-06	0.000162965
HMCN2	0.688392	18.7245	4.765553053	1.55E-15	2.44E-13
HMGA1	16.2438	59.8096	1.8804879	9.80E-06	0.000170982
HNF1A	0	18.0549	Inf	8.54E-06	0.000151469
HOXA-AS3	0	15.9269	Inf	3.64E-06	7.46E-05
HPCAL1	0	19.5078	Inf	4.70E-05	0.000613083
HPN-AS1	0	17.6457	Inf	1.84E-07	5.88E-06
HR	0	23.1316	Inf	2.01E-11	1.73E-09
HRAT92	0	21.6391	Inf	5.57E-07	1.50E-05
HRNR	1.01186	15.574	3.944057932	1.04E-10	7.84E-09
HS6ST1	0	14.1796	Inf	2.82E-05	0.000404432
HSPA2	0	19.919	Inf	3.34E-05	0.000466589
HSPA6	0	23.5822	Inf	3.07E-05	0.000432954
HSPG2	2.03854	17.912	3.135318263	4.88E-15	6.77E-13
HTRA3	0	16.6969	Inf	2.59E-05	0.000377427
HTT	1.80821	9.66086	2.417589384	1.19E-06	2.91E-05
HYDIN	0.446953	7.55136	4.078541461	6.86E-12	6.29E-10
IBA57	1.23605	14.2138	3.523483296	1.08E-07	3.81E-06
ICA1L	0	6.63983	Inf	1.42E-05	0.000232345
IDO1	2.52363	100.008	5.308471195	3.33E-15	4.77E-13
IER5	0	22.6389	Inf	5.12E-05	0.000658307
IFFO2	0.835155	14.184	4.086076653	2.39E-06	5.25E-05
IFITM10	0	20.5709	Inf	5.12E-07	1.40E-05
IFNLR1	0	17.6656	Inf	2.18E-07	6.76E-06
IFRD2	0	26.5708	Inf	2.82E-05	0.000404432
IFT140	0	12.5194	Inf	3.96E-06	7.98E-05
IGDCC3	0	15.4947	Inf	2.00E-06	4.49E-05
IGDCC4	0	12.0311	Inf	3.64E-07	1.04E-05
IGF1R	0	8.06806	Inf	6.07E-09	3.12E-07
IGF2	0	16.9873	Inf	7.85E-10	4.95E-08
IGF2BP1	0.555722	8.40589	3.918965317	1.29E-05	0.000214835
IGFBP4	10.897	103.524	3.247962353	3.89E-14	4.79E-12
IGFN1	0	16.1693	Inf	1.55E-15	2.44E-13
IGSF3	0	10.2955	Inf	6.07E-07	1.62E-05
IGSF9	2.41005	20.4658	3.08608008	2.02E-05	0.000308151
IGSF9B	0	21.4715	Inf	3.07E-09	1.69E-07
IKBKE	0	19.2327	Inf	3.34E-06	6.95E-05
IKZF3	0	8.65738	Inf	6.61E-08	2.47E-06
IL10RA	0	15.1429	Inf	2.18E-05	0.000327135
IL16	1.16862	9.09671	2.960538976	6.73E-05	0.000824237
IL17RA	0	12.9699	Inf	4.70E-10	3.09E-08
IL17RD	1.08388	9.82732	3.180592994	7.54E-06	0.000137281
IL17REL	0	17.195	Inf	7.84E-06	0.000141315
IL1B	0	39.4227	Inf	1.55E-05	0.000248612
IL1RN	4.61252	66.9132	3.858663768	5.12E-10	3.35E-08
IL21R	0	17.6858	Inf	3.34E-08	1.39E-06
IL21R-AS1	0	21.2645	Inf	3.07E-05	0.000432954
IL2RB	0	16.137	Inf	2.18E-06	4.85E-05
IL4R	0	14.0289	Inf	3.34E-05	0.000466589

IL6R	0	10.3743	Inf	2.59E-06	5.60E-05
ILDR2	0	6.57702	Inf	4.70E-05	0.000613083
ING5	2.0971	12.9851	2.630389561	1.11E-06	2.74E-05
INMT	7.61724	75.1281	3.30201236	4.70E-12	4.49E-10
INS-IGF2	0	20.7532	Inf	7.85E-10	4.95E-08
INSRR	0	18.1964	Inf	5.12E-07	1.40E-05
INTS1	4.8913	18.5429	1.922577033	2.25E-05	0.000337253
IP6K1	1.95198	15.0219	2.944057123	7.81E-05	0.000933973
IQSEC1	0	8.15834	Inf	6.61E-06	0.00012264
IQSEC2	3.49729	16.0866	2.2015501	2.12E-05	0.000321455
IQSEC3	1.31795	21.9756	4.059535012	1.02E-11	9.15E-10
IRAIN	0	13.1112	Inf	1.69E-06	3.89E-05
IRGQ	3.01835	13.2161	2.130464496	7.30E-06	0.000133386
IRS4	0	14.6611	Inf	2.38E-05	0.000350829
IRX4	0	20.8742	Inf	5.12E-05	0.000658307
ISG20L2	0	32.9271	Inf	3.64E-08	1.49E-06
ISLR2	0	21.4971	Inf	1.01E-09	6.23E-08
ITGA10	0	9.57184	Inf	8.53E-05	0.000998703
ITGA11	0.972874	14.8018	3.927375849	1.19E-05	0.000201626
ITGA2B	0	17.5892	Inf	1.20E-05	0.000201663
ITGA3	4.83925	22.1737	2.195994147	2.26E-05	0.000338504
ITGA7	0	20.894	Inf	2.18E-08	9.56E-07
ITGAL	0	17.8746	Inf	1.84E-08	8.19E-07
ITGAX	0	12.3736	Inf	1.84E-05	0.000285786
ITGB2	0	26.0965	Inf	1.10E-07	3.84E-06
ITGB4	3.15107	18.3267	2.540033321	3.74E-06	7.64E-05
ITIH5	0	10.2882	Inf	2.18E-07	6.76E-06
ITIH6	0	13.0151	Inf	5.12E-06	9.95E-05
ITPK1	0.977362	20.4896	4.389854997	6.28E-08	2.38E-06
ITPR3	1.61826	13.6943	3.081060199	1.08E-07	3.81E-06
ITPRIP	1.35647	11.8789	3.130472201	1.28E-05	0.000214773
IVL	0	33.0809	Inf	1.55E-06	3.62E-05
JADE1	0.552697	7.38234	3.739517497	6.31E-05	0.000783683
JADE2	0.694772	11.4925	4.048009243	3.57E-06	7.37E-05
JAG2	0	17.6561	Inf	2.59E-09	1.46E-07
JAK3	0	12.2911	Inf	3.34E-06	6.95E-05
JAKMIP3	0	11.6641	Inf	3.34E-07	9.75E-06
JMJD7-PLA	0	20.8681	Inf	1.69E-06	3.89E-05
JPH2	0	18.9373	Inf	3.97E-10	2.65E-08
JPH3	0	16.839	Inf	3.34E-07	9.75E-06
JPH4	0	16.0522	Inf	1.55E-06	3.62E-05
JRK	0	15.6766	Inf	9.33E-13	9.75E-11
KALRN	0.70343	5.16451	2.876152705	1.84E-06	4.19E-05
KANK2	0	9.60081	Inf	7.84E-05	0.000933973
KAZALD1	1.31245	20.8971	3.992968379	6.26E-06	0.000117596
KAZN	1.56057	12.976	3.055700748	1.57E-07	5.17E-06
KBTBD12	0	10.8829	Inf	2.18E-05	0.000327135
KCNA1	0	8.26158	Inf	3.96E-06	7.98E-05
KCNA5	0	17.723	Inf	7.84E-05	0.000933973
KCNA6	0	13.7726	Inf	1.55E-07	5.11E-06
KCNA7	2.22924	20.5079	3.201555917	6.00E-06	0.000113855

KCNAB2	1.69265	15.4217	3.187606215	6.98E-06	0.000128893
KCNB1	0	9.71495	Inf	2.38E-10	1.66E-08
KCNC1	1.16498	15.406	3.725115238	5.08E-09	2.67E-07
KCNC4	1.09829	19.916	4.180596972	8.39E-07	2.16E-05
KCND1	0	14.8862	Inf	1.69E-06	3.89E-05
KCNH2	0	16.6669	Inf	2.59E-08	1.11E-06
KCNH3	0	16.7034	Inf	5.12E-06	9.95E-05
KCNH4	0	18.7895	Inf	8.54E-07	2.17E-05
KCNH6	0	13.2487	Inf	4.31E-05	0.000573472
KCNIP3	0	19.0026	Inf	2.18E-05	0.000327135
KCNJ12	0	25.7152	Inf	5.13E-12	4.80E-10
KCNJ5	0	22.5004	Inf	4.32E-06	8.63E-05
KCNK4-TEX	0	19.7155	Inf	4.31E-05	0.000573472
KCNK5	0	26.8914	Inf	3.34E-09	1.83E-07
KCNK9	0	12.558	Inf	3.07E-05	0.000432954
KCNMA1	0.595482	5.97853	3.327660994	1.40E-06	3.34E-05
KCNN3	0	6.05705	Inf	2.38E-07	7.25E-06
KCNQ1	4.16863	26.0578	2.644070054	2.75E-05	0.000399206
KCNQ1OT1	1.48845	64.1716	5.43005229	1.33E-15	2.44E-13
KCNQ2	1.03761	17.8977	4.108438007	3.61E-12	3.54E-10
KCNS1	0	11.3235	Inf	6.07E-06	0.000114316
KCNT1	0	17.8124	Inf	7.20E-08	2.67E-06
KCP	0	24.3604	Inf	9.31E-12	8.38E-10
KCTD11	0	22.7096	Inf	2.00E-06	4.49E-05
KDM2B	0.847943	11.4759	3.758496204	5.38E-05	0.000688952
KIAA0513	0	13.3848	Inf	2.18E-10	1.53E-08
KIAA0556	1.47158	15.23	3.371478063	8.15E-07	2.10E-05
KIAA1217	0	6.39887	Inf	1.55E-05	0.000248612
KIAA1462	0.523992	7.55514	3.849841799	2.43E-05	0.000358952
KIAA1549L	0	8.08562	Inf	1.55E-08	7.06E-07
KIAA1614	0	21.2677	Inf	4.70E-08	1.85E-06
KIAA1644	0	10.8708	Inf	9.30E-07	2.34E-05
KIAA1656	0	14.567	Inf	5.58E-10	3.61E-08
KIAA1683	0	21.4414	Inf	1.20E-08	5.70E-07
KIAA1755	0	13.7815	Inf	4.70E-08	1.85E-06
KIF13B	1.11182	9.68729	3.123169892	1.38E-05	0.0002283
KIF17	1.18915	15.673	3.720278741	7.39E-05	0.000893921
KIF18B	2.27238	24.9249	3.455311701	2.76E-07	8.30E-06
KIF19	0	15.5006	Inf	2.59E-05	0.000377427
KIF1A	0	17.1037	Inf	3.77E-14	4.67E-12
KIF21B	0	15.9588	Inf	4.46E-14	5.43E-12
KIF24	0	8.51459	Inf	5.12E-05	0.000658307
KIF26A	0	16.9056	Inf	2.82E-10	1.94E-08
KIF26B	0.668743	16.9182	4.660980387	1.15E-09	7.04E-08
KIF3C	0	16.6678	Inf	3.07E-08	1.29E-06
KIF7	0	16.4556	Inf	6.61E-07	1.74E-05
KIFC2	0	16.63	Inf	3.96E-05	0.000536192
KIRREL	0	10.2528	Inf	2.38E-07	7.25E-06
KLF7	0	5.97009	Inf	5.12E-05	0.000658307
KLHDC7A	0	15.3889	Inf	3.64E-07	1.04E-05
KLHL29	0	11.6159	Inf	1.55E-05	0.000248612

KLHL3	0	7.44434	Inf	1.69E-05	0.000266218
KLHL30	0	13.8033	Inf	7.84E-05	0.000933973
KMT2B	1.15054	14.1465	3.620062112	2.65E-08	1.13E-06
KMT2D	3.2623	12.9191	1.985544206	8.33E-10	5.24E-08
KNDC1	0.878199	14.9539	4.089830089	5.40E-12	5.04E-10
KREMEN1	1.30315	18.5012	3.827543787	8.92E-10	5.57E-08
KRT17P5	0	43.3384	Inf	3.35E-12	3.29E-10
KRT78	0	18.954	Inf	2.38E-05	0.000350829
KRT81	0	32.0474	Inf	1.01E-05	0.000174751
KSR1	0	16.2211	Inf	1.01E-06	2.52E-05
KSR2	0.57304	9.09869	3.988951093	4.33E-11	3.56E-09
KY	0	12.5449	Inf	1.31E-06	3.13E-05
L1CAM	0.929631	21.1336	4.506736555	1.23E-08	5.82E-07
L2HGDH	5.5967	127.797	4.513133743	1.11E-15	2.44E-13
LAIR1	0	35.63	Inf	5.12E-11	4.15E-09
LAMA1	0	6.3831	Inf	9.30E-06	0.000162965
LAMA5	0	17.7236	Inf	1.33E-15	2.44E-13
LAMB2	0.839037	17.664	4.395933835	5.79E-08	2.22E-06
LAMC3	0	18.167	Inf	5.58E-10	3.61E-08
LAPTM5	6.52651	40.7967	2.644068826	2.75E-05	0.000399206
LBH	0	17.1661	Inf	8.53E-05	0.000998703
LCP1	2.57157	23.6571	3.201551902	6.00E-06	0.000113855
LCP2	0	13.0411	Inf	2.82E-05	0.000404432
LCT	0	8.30678	Inf	6.07E-05	0.000757821
LDB3	0.747183	10.4427	3.804889333	3.62E-05	0.00050071
LDLR	5.53345	23.5761	2.091073926	1.24E-05	0.000207789
LDLRAD2	0	19.1248	Inf	4.70E-07	1.31E-05
LENG9	0	29.6508	Inf	2.38E-05	0.000350829
LETM1P2	0	15.5777	Inf	2.00E-05	0.000306049
LGALS1	52.3992	322.909	2.623510961	1.70E-09	9.91E-08
LGI3	0	15.4421	Inf	8.53E-05	0.000998703
LGI4	0	22.6874	Inf	4.32E-06	8.63E-05
LHFPL4	0	19.5214	Inf	1.20E-08	5.70E-07
LHX3	0	21.1078	Inf	3.34E-05	0.000466589
LHX5	0	24.4074	Inf	7.84E-05	0.000933973
LILRB4	0	14.6902	Inf	2.00E-05	0.000306049
LIMD1	0	8.98613	Inf	2.59E-05	0.000377427
LIMD2	0	18.7127	Inf	1.20E-05	0.000201663
LIMS2	0	19.9487	Inf	9.30E-06	0.000162965
LINC00174	0	11.4001	Inf	8.53E-05	0.000998703
LINC00176	0	18.8962	Inf	8.53E-05	0.000998703
LINC00202	0	8.47431	Inf	6.61E-05	0.000810778
LINC00294	0	28.8243	Inf	1.20E-08	5.70E-07
LINC00342	0	22.1869	Inf	2.00E-06	4.49E-05
LINC00482	0	18.7228	Inf	3.07E-05	0.000432954
LINC00504	11.4735	724.83	5.98126521	1.11E-15	2.44E-13
LINC00506	2.10049	276.752	7.041724015	1.55E-15	2.44E-13
LINC00514	0	25.5611	Inf	1.55E-07	5.11E-06
LINC00639	0	15.1161	Inf	4.70E-06	9.28E-05
LINC00649	0	35.1917	Inf	6.07E-09	3.12E-07
LINC00661	0	17.7996	Inf	1.42E-05	0.000232345

LINC008240	18.3996	Inf	1.31E-06	3.13E-05
LINC008420	17.8297	Inf	7.20E-05	0.000872523
LINC008980	12.4631	Inf	6.07E-05	0.000757821
LINC009100	11.4649	Inf	8.53E-05	0.000998703
LINC009500	10.5197	Inf	3.34E-05	0.000466589
LINC009570	47.105	Inf	1.42E-07	4.77E-06
LINC009650	79.1926	Inf	1.33E-15	2.44E-13
LINC009820	15.235	Inf	1.69E-05	0.000266218
LINC0100C3.32377	28.1829	3.083927613	1.33E-15	2.44E-13
LINC011010	27.5964	Inf	3.07E-05	0.000432954
LINC011050	5.19393	Inf	9.30E-06	0.000162965
LINC011280	10.3645	Inf	1.10E-08	5.30E-07
LINC011590	13.0197	Inf	6.61E-05	0.000810778
LINC011680	21.8845	Inf	1.31E-10	9.69E-09
LINC013100	15.997	Inf	5.57E-06	0.000106758
LINC013140	12.2929	Inf	8.54E-08	3.08E-06
LINC013910	14.2549	Inf	8.53E-05	0.000998703
LINC014510	17.1056	Inf	1.84E-05	0.000285786
LINC014880	17.9975	Inf	9.30E-06	0.000162965
LINC015020	26.4715	Inf	1.84E-06	4.18E-05
LINC015300	44.3477	Inf	6.07E-07	1.62E-05
LINC015310	19.9131	Inf	3.96E-06	7.98E-05
LINC017490	16.7762	Inf	2.59E-05	0.000377427
LINC018147.16718	281.537	5.295775138	1.33E-15	2.44E-13
LINC0187366.7552	3183.01	5.575367657	1.33E-15	2.44E-13
LINC019680	17.0202	Inf	8.54E-06	0.000151469
LINC019790	15.4563	Inf	1.84E-07	5.88E-06
LINC020120	13.6176	Inf	1.31E-05	0.000216387
LINC0204315.96	226.118	3.824543285	1.33E-15	2.44E-13
LINC020760	35.7878	Inf	3.07E-06	6.49E-05
LINC020780	12.7508	Inf	4.70E-05	0.000613083
LINC021080	352.785	Inf	1.33E-15	2.44E-13
LINGO1 0	12.6348	Inf	1.55E-05	0.000248612
LMF1 0	16.2664	Inf	5.57E-07	1.50E-05
LMTK3 0	16.1257	Inf	2.38E-07	7.25E-06
LMX1B 0	19.9633	Inf	1.84E-10	1.32E-08
LOC1001290	17.7905	Inf	6.07E-06	0.000114316
LOC1001271.4884	2044.49	4.837888033	1.55E-15	2.44E-13
LOC1001300	21.1249	Inf	1.69E-07	5.47E-06
LOC1001300	14.1642	Inf	3.64E-05	0.00050071
LOC1001310.927464	9.18851	3.308467745	4.04E-12	3.88E-10
LOC1001322.37772	33.3364	3.809447577	1.22E-09	7.42E-08
LOC1001330	25.5532	Inf	1.55E-07	5.11E-06
LOC1001330	22.1468	Inf	1.42E-05	0.000232345
LOC1001907.9464	46.2164	2.540031565	3.74E-06	7.64E-05
LOC1002870	23.9017	Inf	8.54E-06	0.000151469
LOC1002870	11.1826	Inf	1.55E-05	0.000248612
LOC1002870	17.6318	Inf	3.97E-11	3.29E-09
LOC1002890	24.1652	Inf	1.01E-05	0.000174751
LOC1004190	31.9041	Inf	8.54E-09	4.24E-07
LOC1004991.39972	11.8862	3.086077397	2.02E-05	0.000308151

LOC1005053.75145	43.8029	3.545506153	7.93E-08	2.91E-06
LOC1005060	7.52639	Inf	2.82E-09	1.57E-07
LOC1005060	14.3755	Inf	1.20E-05	0.000201663
LOC1005060	13.2264	Inf	2.38E-05	0.000350829
LOC1005070	11.1809	Inf	1.10E-05	0.000187799
LOC1009960	51.6992	Inf	6.07E-06	0.000114316
LOC1019264.47076	41.7219	3.222212853	4.77E-06	9.41E-05
LOC1019260.5361	1529.46	4.919564234	1.11E-15	2.44E-13
LOC1019260.775729	19.0759	4.620054578	2.22E-09	1.26E-07
LOC1019270	23.6948	Inf	6.07E-09	3.12E-07
LOC1019274.32398	415.569	6.586584378	1.55E-15	2.44E-13
LOC1019270	16.04	Inf	3.64E-05	0.00050071
LOC1019270	23.6342	Inf	2.00E-05	0.000306049
LOC1019270	17.9169	Inf	7.20E-05	0.000872523
LOC1019270	19.7495	Inf	1.20E-06	2.91E-05
LOC1019280	14.5156	Inf	1.55E-07	5.11E-06
LOC1019280	19.8563	Inf	2.00E-05	0.000306049
LOC1019282.07257	16.0874	2.956438447	6.17E-09	3.16E-07
LOC1019281.22625	312.502	7.993468476	1.55E-15	2.44E-13
LOC1019290	22.0178	Inf	1.84E-05	0.000285786
LOC1019290	21.0108	Inf	2.38E-07	7.25E-06
LOC1027230	65.9084	Inf	2.82E-08	1.19E-06
LOC1027240	36.9411	Inf	3.64E-06	7.46E-05
LOC1027240	33.3702	Inf	1.20E-07	4.12E-06
LOC1027240	27.0485	Inf	4.70E-08	1.85E-06
LOC1027250	16.9955	Inf	3.97E-09	2.13E-07
LOC1031710	57.2116	Inf	6.61E-07	1.74E-05
LOC1033440	12.5137	Inf	5.12E-05	0.000658307
LOC1053730	12.9296	Inf	7.84E-05	0.000933973
LOC1151100	16.9848	Inf	3.34E-07	9.75E-06
LOC1486960	13.7964	Inf	1.84E-05	0.000285786
LOC2840230	21.5103	Inf	3.96E-05	0.000536192
LOC2844122.07898	38.435	4.208472884	6.07E-07	1.62E-05
LOC2862970	32.2754	Inf	1.20E-07	4.12E-06
LOC3889960	69.7164	Inf	2.18E-11	1.87E-09
LOC4000022.46304	250.117	6.66601925	1.55E-15	2.44E-13
LOC4010520	34.0849	Inf	5.12E-06	9.95E-05
LOC4012860	16.6819	Inf	6.07E-07	1.62E-05
LOC4013570	40.5647	Inf	4.72E-13	5.20E-11
LOC4403001.88127	52.3085	4.797266575	1.55E-15	2.44E-13
LOC4410816.45203	100.04	3.954680006	1.11E-15	2.44E-13
LOC4412421.42698	19.5649	3.777230717	4.59E-05	0.000609123
LOC6428520	9.92671	Inf	1.42E-06	3.37E-05
LOC6429430	15.6113	Inf	3.07E-07	9.07E-06
LOC6431720	21.613	Inf	3.96E-05	0.000536192
LOC6434062.56548	78.7459	4.939904126	1.55E-15	2.44E-13
LOC6462143.55443	157.311	5.467857522	1.33E-15	2.44E-13
LOC6465550	14.8284	Inf	9.30E-06	0.000162965
LOC6489870	27.0411	Inf	1.31E-08	6.13E-07
LOC7287430	17.529	Inf	1.55E-05	0.000248612
LOC7287520	50.3005	Inf	1.42E-06	3.37E-05

LOC729218	0	16.718	Inf	7.20E-09	3.62E-07
LOC729603	0	18.1731	Inf	2.38E-05	0.000350829
LOC729732	0	20.7844	Inf	1.42E-07	4.77E-06
LOC730234	0	38.0965	Inf	1.55E-14	2.01E-12
LOC730668	0	16.9855	Inf	1.55E-06	3.62E-05
LOXHD1	0.619282	11.8324	4.256002406	3.45E-07	9.99E-06
LPIN3	0	16.5194	Inf	9.30E-07	2.34E-05
LPP	2.37276	33.0688	3.800832607	1.33E-15	2.44E-13
LRCH4	0	18.7823	Inf	1.10E-05	0.000187799
LRFN2	0	15.6579	Inf	8.53E-05	0.000998703
LRFN4	0	19.9502	Inf	8.53E-05	0.000998703
LRP1	1.63473	16.2244	3.311040848	7.33E-15	1.00E-12
LRP3	0	17.8346	Inf	3.96E-06	7.98E-05
LRP4	1.18467	11.7367	3.308469679	1.76E-06	4.04E-05
LRP5	0.910355	14.2532	3.968712801	7.96E-06	0.000143202
LRPAP1	5.00835	17.7209	1.823044585	4.96E-07	1.37E-05
LRRC14	0	13.5573	Inf	8.54E-07	2.17E-05
LRRC15	0	11.4659	Inf	2.59E-06	5.60E-05
LRRC20	2.68862	21.2854	2.984926432	5.38E-05	0.000688905
LRRC27	0	11.4882	Inf	9.31E-10	5.77E-08
LRRC28	0.773267	10.9441	3.823042862	3.09E-05	0.000435638
LRRC32	2.19214	16.7731	2.935737509	8.42E-05	0.000997309
LRRC37A4P	1.34654	12.8639	3.255999105	3.25E-06	6.85E-05
LRRC37A6P	0	10.0184	Inf	5.12E-06	9.95E-05
LRRC7	0	6.45742	Inf	7.84E-05	0.000933973
LRRC74B	0	12.4981	Inf	5.57E-06	0.000106758
LRRC8E	0	13.5821	Inf	7.84E-05	0.000933973
LRRK1	3.26461	11.6335	1.833302646	6.97E-05	0.000852231
LRRN2	0	16.3398	Inf	3.96E-05	0.000536192
LRTM2	0	18.1544	Inf	1.10E-06	2.72E-05
LRWD1	0	24.0213	Inf	3.64E-05	0.00050071
LSM11	0	9.62421	Inf	6.61E-06	0.00012264
LSP1	0	18.7791	Inf	3.96E-06	7.98E-05
LSR	0	24.4103	Inf	3.07E-05	0.000432954
LTB4R	0	13.8088	Inf	6.07E-06	0.000114316
LTBP2	0	16.3777	Inf	1.69E-12	1.69E-10
LTBP3	1.03639	19.4353	4.229040472	4.77E-07	1.32E-05
LTBP4	1.79027	21.9331	3.614860646	2.87E-08	1.21E-06
LTK	0	16.2664	Inf	7.84E-05	0.000933973
LYNX1	1.77463	19.8576	3.484101101	1.87E-07	5.96E-06
LYRM7	0	8.90135	Inf	3.07E-05	0.000432954
LZTS1	0	18.0826	Inf	6.07E-09	3.12E-07
MAB21L3	4.52753	584.447	7.01220419	1.55E-15	2.44E-13
MADD	0.760001	12.0336	3.98492318	6.78E-06	0.000125577
MAFB	0	14.9912	Inf	7.84E-05	0.000933973
MAG	0	24.3354	Inf	9.30E-06	0.000162965
MAGEC1	0	12.5697	Inf	3.96E-05	0.000536192
MALL	0	17.6029	Inf	4.31E-05	0.000573472
MAMDC4	0	23.673	Inf	6.61E-08	2.47E-06
MAML1	0	11.5241	Inf	3.96E-06	7.98E-05
MAN1C1	0	14.6752	Inf	2.59E-06	5.60E-05

MAN2A2	0.68108	10.9648	4.008911419	5.33E-06	0.000103296
MAP1A	0.948541	17.0326	4.166444732	9.99E-13	1.03E-10
MAP1B	0.79354	6.38763	3.008905896	4.29E-05	0.000573472
MAP1LC3C	0	59.3069	Inf	1.55E-06	3.62E-05
MAP1S	0	15.5964	Inf	2.82E-05	0.000404432
MAP2K3	0	21.3806	Inf	4.70E-05	0.000613083
MAP2K7	0	16.7756	Inf	1.42E-05	0.000232345
MAP3K14	0	17.5001	Inf	3.34E-07	9.75E-06
MAP3K9	0.837955	9.71006	3.534535535	9.27E-08	3.31E-06
MAP7D3	1.49094	11.7376	2.976843344	5.79E-05	0.00073122
MAPK7	0	17.9176	Inf	1.42E-05	0.000232345
MAPK8IP1	0	20.1744	Inf	5.12E-06	9.95E-05
MAPK8IP2	0	17.0995	Inf	2.00E-05	0.000306049
MAPK8IP3	0.860369	19.4068	4.495462897	1.45E-08	6.67E-07
MAPKBP1	0.672156	13.7939	4.359090497	9.43E-08	3.35E-06
MAPT	0	12.3955	Inf	1.01E-07	3.58E-06
MAPT-IT1	0	18.5802	Inf	2.82E-05	0.000404432
MARK4	0.929454	16.1144	4.115823196	1.73E-06	3.98E-05
MAST1	0	16.8116	Inf	1.42E-07	4.77E-06
MAST3	0	15.2147	Inf	3.64E-08	1.49E-06
MAST4	1.23402	9.42386	2.93295233	8.78E-07	2.24E-05
MC1R	0	18.4998	Inf	2.18E-05	0.000327135
MCF2L	0	11.8688	Inf	3.96E-08	1.62E-06
MDC1	1.32278	11.2913	3.093566554	1.87E-05	0.000290534
MDGA1	1.09422	22.504	4.362206722	9.99E-15	1.34E-12
MDN1	0.80291	5.20833	2.697510674	1.54E-05	0.000248612
MECP2	0.93633	10.146	3.437750173	3.48E-07	1.01E-05
MED12	1.39852	13.4223	3.262659117	3.01E-06	6.43E-05
MEF2D	0	14.8348	Inf	3.34E-08	1.39E-06
MEFV	0	14.0793	Inf	6.61E-05	0.000810778
MEG3	0.922068	10.9294	3.567197243	5.80E-08	2.23E-06
MEGF6	1.31016	19.1222	3.867433601	4.36E-10	2.89E-08
MEGF8	1.30916	17.525	3.742701111	2.66E-13	2.99E-11
METRN	2.98324	23.6179	2.984928741	5.38E-05	0.000688905
MEX3A	0	11.7549	Inf	1.20E-06	2.91E-05
MFNG	0	24.2169	Inf	6.61E-05	0.000810778
MFRP	0	15.7049	Inf	9.30E-06	0.000162965
MFSD2A	0	23.3742	Inf	6.61E-05	0.000810778
MFSD4B	0	14.9551	Inf	1.69E-05	0.000266218
MGAT3	0	14.399	Inf	6.61E-07	1.74E-05
MGAT4A	0	10.1753	Inf	6.61E-08	2.47E-06
MGAT5B	0	17.8316	Inf	2.82E-07	8.42E-06
MGC27345	0	11.5474	Inf	1.84E-10	1.32E-08
MGLL	0	16.4034	Inf	3.07E-07	9.07E-06
MGRN1	3.76536	18.4521	2.292925306	6.68E-06	0.000123809
MIAT	0	7.49265	Inf	5.12E-07	1.40E-05
MICAL3	3.9576	14.674	1.890564481	3.40E-08	1.41E-06
MICALL2	0	16.2743	Inf	8.53E-05	0.000998703
MIEF1	1.60882	12.3099	2.935744215	8.42E-05	0.000997309
MIEF2	0	19.5566	Inf	6.61E-05	0.000810778
MIER2	0	18.7017	Inf	5.57E-05	0.000705723

MIGA2	0.986264	17.1866	4.123166482	1.60E-06	3.72E-05
MIR600HG	1.62872	13.0384	3.00095633	4.63E-05	0.000611824
MIR9-3HG	0	13.3343	Inf	1.84E-06	4.18E-05
MIRLET7BH	2.0662	23.303	3.495463884	1.60E-07	5.24E-06
MISP	0	20.3203	Inf	1.69E-05	0.000266218
MKI67	2.72961	20.1095	2.881110469	6.51E-14	7.75E-12
MLLT6	5.198	20.0012	1.94405802	3.81E-06	7.75E-05
MLPH	0	14.1677	Inf	1.10E-05	0.000187799
MMEL1	0	17.6761	Inf	7.20E-05	0.000872523
MMP15	0	20.3866	Inf	6.61E-08	2.47E-06
MMP24	0	14.5245	Inf	7.20E-06	0.000131598
MMP25-AS1	0	24.3266	Inf	2.38E-05	0.000350829
MMP9	0	23.0661	Inf	4.31E-05	0.000573472
MMRN2	0	14.5405	Inf	1.10E-05	0.000187799
MN1	0	12.3796	Inf	1.69E-08	7.60E-07
MNT	0	11.0391	Inf	4.70E-05	0.000613083
MORN1	3.05573	16.5558	2.437747746	1.33E-05	0.00022015
MOV10	1.0834	16.1001	3.893431748	1.63E-05	0.000260839
MPO	0	20.5139	Inf	3.96E-06	7.98E-05
MPP2	0	17.1627	Inf	9.30E-09	4.55E-07
MPRIP	2.21044	10.4021	2.234469333	1.41E-05	0.000232345
MR1	0	6.70153	Inf	4.70E-05	0.000613083
MRC2	3.25309	18.92	2.540029447	3.74E-06	7.64E-05
MRGPRX3	0	48.9183	Inf	2.38E-07	7.25E-06
MROH2A	0	15.3834	Inf	1.69E-07	5.47E-06
MROH5	1.08148	15.2105	3.813988688	3.34E-05	0.000466775
MROH7-TTC	0	10.0048	Inf	4.32E-06	8.63E-05
MRPL57	0	21.5692	Inf	2.18E-05	0.000327135
MST1L	2.08789	19.2075	3.201552143	6.00E-06	0.000113855
MST1P2	0	28.9445	Inf	5.12E-07	1.40E-05
MST1R	0	19.6714	Inf	3.96E-08	1.62E-06
MTA3	2.17486	12.7612	2.552769559	6.99E-05	0.000853216
MTCL1	0.799923	15.2838	4.25599834	3.45E-07	9.99E-06
MTUS2	0.6772	12.4598	4.201555127	6.58E-07	1.74E-05
MUC12	0	32.7765	Inf	1.33E-15	2.44E-13
MUC16	0.222436	11.963	5.749045115	1.55E-15	2.44E-13
MUC17	0	12.7666	Inf	1.78E-15	2.69E-13
MUC19	0.591256	9.48381	4.003611936	1.78E-15	2.69E-13
MUC2	0	16.5869	Inf	9.33E-13	9.75E-11
MUC22	0	8.90115	Inf	4.70E-05	0.000613083
MUC3A	0	19.1976	Inf	4.32E-10	2.86E-08
MUC5AC	0	18.3878	Inf	7.85E-10	4.95E-08
MUC5B	0.543997	21.3894	5.297153508	1.55E-15	2.44E-13
MUC6	0.608913	37.0034	5.925277912	1.55E-15	2.44E-13
MVB12B	1.44175	14.2836	3.308466711	1.76E-06	4.04E-05
MXRA5	0	7.65742	Inf	6.61E-07	1.74E-05
MXRA7	1.54164	16.0914	3.383752035	6.98E-07	1.83E-05
MXRA8	0	20.6322	Inf	5.57E-06	0.000106758
MYADML2	0	26.5457	Inf	4.70E-06	9.28E-05
MYBPC2	0	18.9293	Inf	2.59E-06	5.60E-05
MYBPC3	0	13.5952	Inf	2.18E-05	0.000327135

MYCL	0	15.1493	Inf	6.61E-07	1.74E-05
MYH11	2.80791	17.076	2.604401468	1.59E-06	3.71E-05
MYH13	0	10.0715	Inf	1.20E-05	0.000201663
MYH3	0	9.20946	Inf	3.07E-05	0.000432954
MYH6	0	14.9467	Inf	4.32E-08	1.73E-06
MYH7	0	18.0113	Inf	7.85E-10	4.95E-08
MYH7B	0.740147	16.5641	4.484104176	1.71E-08	7.67E-07
MYLK	2.40441	11.6445	2.275893863	8.04E-05	0.000955605
MYLK2	0	20.3723	Inf	2.38E-05	0.000350829
MYO15A	0	12.6451	Inf	2.39E-13	2.70E-11
MYO15B	0	17.4882	Inf	9.99E-15	1.34E-12
MYO16	0	6.81767	Inf	7.20E-05	0.000872523
MYO18B	1.13632	12.6649	3.47839461	2.02E-07	6.35E-06
MYO1F	0	17.2507	Inf	1.20E-06	2.91E-05
MYO7A	0	14.5801	Inf	3.64E-10	2.46E-08
MYO7B	0	18.3594	Inf	4.71E-11	3.84E-09
MYO9B	0.639266	16.5684	4.695874114	6.48E-10	4.17E-08
MYOM3	0.840195	11.7427	3.804896155	3.62E-05	0.00050071
MYRF	0	19.4825	Inf	1.69E-10	1.23E-08
MYT1	0	11.2924	Inf	7.84E-06	0.000141315
NACAD	0	15.3012	Inf	1.01E-06	2.52E-05
NACC2	0	10.6843	Inf	8.54E-07	2.17E-05
NAT8L	1.66007	13.9502	3.070969824	2.35E-05	0.000350829
NAV1	0.367367	10.4637	4.832027236	5.99E-11	4.77E-09
NAV2	1.24336	10.9983	3.144964567	4.04E-08	1.64E-06
NBL1	0	22.9916	Inf	6.07E-07	1.62E-05
NBPF1	0.827216	14.2687	4.108445998	1.88E-06	4.26E-05
NCAM1	0	10.0105	Inf	8.54E-08	3.08E-06
NCAN	0	12.3814	Inf	2.82E-07	8.42E-06
NCKAP5L	1.98903	17.8582	3.166449732	8.78E-06	0.000155394
NCKIPSD	0	17.2424	Inf	6.61E-05	0.000810778
NCOR2	1.64614	16.4542	3.321296941	2.06E-09	1.19E-07
NDOR1	1.99841	19.1798	3.26266317	3.01E-06	6.43E-05
NDRG4	0	18.7983	Inf	2.00E-07	6.31E-06
NDUFA10	2.00314	11.7389	2.550962066	3.25E-06	6.85E-05
NECAB3	0	25.1575	Inf	7.20E-05	0.000872523
NECTIN1	1.59409	13.9597	3.130462949	1.28E-05	0.000214773
NECTIN2	1.36311	20.2568	3.893432393	1.63E-05	0.000260839
NEFH	0	26.2969	Inf	7.20E-09	3.62E-07
NEFHP1	0	18.3302	Inf	5.57E-05	0.000705723
NEFL	0	13.5793	Inf	6.07E-05	0.000757821
NEFM	0	17.8271	Inf	5.12E-06	9.95E-05
NES	0	19.0789	Inf	1.43E-09	8.49E-08
NEURL1	1.12935	15.0846	3.739511871	6.31E-05	0.000783683
NEURL1B	0	12.4809	Inf	2.38E-07	7.25E-06
NEURL4	0	18.6984	Inf	8.54E-09	4.24E-07
NEUROD2	0	16.7761	Inf	7.84E-05	0.000933973
NFAM1	1.73885	16.6118	3.256003017	3.25E-06	6.85E-05
NFASC	0	12.7092	Inf	1.55E-12	1.56E-10
NFATC1	0	9.82972	Inf	3.07E-05	0.000432954
NFATC4	1.55022	15.0154	3.275898032	2.58E-06	5.60E-05

NFKBIB	0	23.1417	Inf	3.96E-05	0.000536192
NFKBIE	0	19.7075	Inf	7.84E-05	0.000933973
NGEF	0	18.279	Inf	7.20E-06	0.000131598
NGFR	0	17.2676	Inf	1.55E-05	0.000248612
NHS	0	5.9004	Inf	4.31E-05	0.000573472
NICN1	0	22.0406	Inf	1.42E-06	3.37E-05
NINJ1	0	59.2245	Inf	5.57E-07	1.50E-05
NIPAL3	0	7.58277	Inf	2.59E-05	0.000377427
NKD1	1.1182	10.3363	3.208469693	5.56E-06	0.000106758
NKPD1	0	15.4508	Inf	1.10E-06	2.72E-05
NLGN2	0	16.7006	Inf	3.96E-07	1.12E-05
NLGN4X	0	7.74475	Inf	6.61E-05	0.000810778
NLRC3	0	14.7337	Inf	7.84E-09	3.92E-07
NLRC5	2.85187	16.3973	2.523478154	4.63E-06	9.22E-05
NLRP12	0	17.5399	Inf	2.59E-06	5.60E-05
NMNAT2	0	9.42126	Inf	3.96E-05	0.000536192
NOC2LP1	0	18.227	Inf	8.53E-05	0.000998703
NOL12	0	19.5798	Inf	3.07E-05	0.000432954
NOL6	0	14.7447	Inf	1.31E-06	3.13E-05
NOS1	0	12.3809	Inf	8.68E-14	1.02E-11
NOS2	0	14.6556	Inf	9.30E-06	0.000162965
NOS3	0	13.4627	Inf	2.18E-06	4.85E-05
NOTCH1	2.09462	12.9698	2.63039579	1.11E-06	2.74E-05
NOTCH2	0	8.93105	Inf	1.55E-09	9.13E-08
NOTCH3	1.20756	16.5032	3.772579014	2.31E-09	1.31E-07
NOTCH4	0	16.1797	Inf	5.58E-10	3.61E-08
NPAP1	0	10.2774	Inf	1.42E-07	4.77E-06
NPC1L1	0	20.1085	Inf	1.55E-09	9.13E-08
NPHP4	0	12.4911	Inf	2.00E-06	4.49E-05
NPHS1	0	15.3582	Inf	4.32E-07	1.21E-05
NPR1	0	14.1112	Inf	1.55E-05	0.000248612
NPTX1	0	15.0637	Inf	1.69E-07	5.47E-06
NPTXR	0	15.0482	Inf	5.58E-08	2.15E-06
NR2F1-AS1	0	7.8572	Inf	5.12E-05	0.000658307
NR2F2	0.797566	11.3585	3.832024606	2.85E-05	0.000409114
NR4A1	2.47241	19.3551	2.968723851	6.24E-05	0.000777378
NR6A1	0	9.45842	Inf	3.34E-06	6.95E-05
NRG1	0	8.50441	Inf	1.69E-08	7.60E-07
NRIP2	0	18.9375	Inf	5.57E-05	0.000705723
NRP2	0	8.32076	Inf	5.57E-06	0.000106758
NRXN2	0	16.1356	Inf	1.01E-10	7.68E-09
NRXN3	0	6.70631	Inf	9.30E-09	4.55E-07
NSD2	1.71217	6.76489	1.982240523	4.85E-05	0.000632797
NT5DC3	0	7.46916	Inf	4.31E-05	0.000573472
NTN1	0	11.4389	Inf	2.59E-06	5.60E-05
NTSR1	0	15.6484	Inf	5.12E-06	9.95E-05
NUAK1	0	12.4496	Inf	9.30E-08	3.31E-06
NUAK2	0	21.1956	Inf	1.01E-06	2.52E-05
NUP210	1.35214	13.5155	3.321298444	1.51E-06	3.57E-05
NUTM2B-AS	0	9.93607	Inf	6.61E-05	0.000810778
NUTM2D	0	30.9511	Inf	4.66E-15	6.49E-13

NUTM2G	0.928392	25.4579	4.777235516	1.61E-10	1.17E-08
NWD1	0	12.9267	Inf	7.21E-10	4.58E-08
NXPH3	1.75513	13.8175	2.976846815	5.79E-05	0.00073122
NYNRIN	0	15.3068	Inf	8.55E-11	6.57E-09
OBSCN	1.02133	16.4877	4.012869167	1.33E-15	2.44E-13
OBSL1	0.669846	17.1239	4.676038052	9.00E-10	5.59E-08
ODF2L	3.76536	20.6504	2.455310017	6.82E-07	1.79E-05
OGFOD2	0	21.4834	Inf	1.31E-05	0.000216387
OGFOD3	0	14.6138	Inf	3.96E-06	7.98E-05
OPA3	2.03833	12.8917	2.660982977	7.23E-07	1.88E-05
OPHN1	15.5298	417.609	4.749041649	1.55E-15	2.44E-13
OPRL1	0	15.9826	Inf	1.69E-05	0.000266218
ORAI2	0	16.4274	Inf	2.44E-15	3.58E-13
ORC4	24.9229	611.592	4.617025682	1.33E-15	2.44E-13
OSBP2	0	12.7825	Inf	3.34E-06	6.95E-05
OSBPL7	0	16.5067	Inf	1.20E-05	0.000201663
OTOF	0	16.7239	Inf	3.64E-11	3.04E-09
OTOG	0	18.5318	Inf	1.69E-14	2.17E-12
OTOP1	0	28.8296	Inf	4.31E-05	0.000573472
OTUB1	1.94769	26.0152	3.739518821	6.31E-05	0.000783683
OTUD3	0	7.93971	Inf	6.61E-05	0.000810778
OTUD7A	0	7.69818	Inf	5.58E-08	2.15E-06
P2RX5-TAX	0	19.2725	Inf	7.21E-11	5.67E-09
P2RX6	0	24.8205	Inf	2.59E-06	5.60E-05
P2RY2	2.18135	15.2434	2.804891552	8.17E-08	2.99E-06
PACS2	2.13017	18.5915	3.125602699	5.47E-08	2.13E-06
PACSIN1	0	23.2876	Inf	1.84E-09	1.07E-07
PADI1	0	17.704	Inf	2.59E-06	5.60E-05
PAK6	0	16.9862	Inf	3.07E-07	9.07E-06
PALD1	0	17.265	Inf	3.07E-07	9.07E-06
PALM3	0	32.4249	Inf	9.30E-07	2.34E-05
PANK1	0	7.57406	Inf	3.96E-05	0.000536192
PANK2	1.06168	13.0539	3.620059997	2.65E-08	1.13E-06
PAPLN	0	16.1075	Inf	1.55E-08	7.06E-07
PAPPA2	0.63312	8.79257	3.79573404	3.92E-05	0.000536192
PAQR4	0	21.9157	Inf	1.01E-05	0.000174751
PARP15	2.80387	103.342	5.203863439	1.55E-15	2.44E-13
PARP3	0	22.0918	Inf	5.12E-05	0.000658307
PART1	0	6.26237	Inf	1.42E-05	0.000232345
PASK	0	10.4579	Inf	4.70E-06	9.28E-05
PAX1	0	12.6451	Inf	2.82E-06	6.03E-05
PAX2	0	14.9659	Inf	6.61E-06	0.00012264
PAX5	0	13.8943	Inf	3.64E-11	3.04E-09
PAX6	0	6.54982	Inf	8.54E-06	0.000151469
PAX7	0	7.98891	Inf	8.53E-05	0.000998703
PBXIP1	0	17.527	Inf	2.18E-05	0.000327135
PCDH1	0	16.1216	Inf	3.07E-08	1.29E-06
PCDH10	0	6.65048	Inf	6.61E-06	0.00012264
PCDH11Y	0	13.544	Inf	2.84E-13	3.16E-11
PCDH12	0.742628	10.0506	3.758498108	5.38E-05	0.000688952
PCDH19	0	7.55548	Inf	8.54E-07	2.17E-05

PCDH7	0.980706	8.02436	3.032493722	3.43E-05	0.00047778
PCDH8	0	18.9524	Inf	5.57E-07	1.50E-05
PCDHAC2	0	9.07717	Inf	3.07E-05	0.000432954
PCDHB7	0	16.7558	Inf	7.20E-06	0.000131598
PCDHB8	0	28.9387	Inf	3.34E-07	9.75E-06
PCLO	0	5.99151	Inf	6.08E-12	5.62E-10
PCNX3	0.742176	16.7408	4.495463313	1.45E-08	6.67E-07
PCSK5	0.799463	6.39995	3.000957463	4.63E-05	0.000611824
PDDC1	4.17488	23.3579	2.484103939	7.59E-06	0.000138112
PDE1B	0	16.5223	Inf	7.84E-06	0.000141315
PDE2A	1.03772	15.4212	3.893425903	1.63E-05	0.000260839
PDE4A	0	13.5877	Inf	2.59E-07	7.83E-06
PDE4C	0	14.6501	Inf	5.58E-08	2.15E-06
PDE4DIP	2.89271	24.6603	3.091696748	1.33E-15	2.44E-13
PDE6A	0	11.7659	Inf	3.64E-06	7.46E-05
PDGFRB	0.85284	14.1826	4.055703121	3.30E-06	6.92E-05
PDLIM2	0	16.1068	Inf	1.43E-08	6.56E-07
PDLIM4	0	27.7982	Inf	6.07E-06	0.000114316
PDPR	1.04205	10.9689	3.395922446	5.98E-07	1.61E-05
PDX1	0	19.7688	Inf	7.84E-05	0.000933973
PDZD4	0	17.7937	Inf	7.20E-07	1.87E-05
PDZD7	0	15.7015	Inf	2.00E-06	4.49E-05
PDZD8	0	12.4983	Inf	2.00E-07	6.31E-06
PEAK1	0.824766	6.71194	3.024673054	3.69E-05	0.000508002
PEAR1	0	14.5281	Inf	1.55E-06	3.62E-05
PEG13	0	13.1411	Inf	7.84E-07	2.02E-05
PERM1	0	17.472	Inf	1.20E-05	0.000201663
PFAS	0.904945	17.8508	4.302014809	1.96E-07	6.23E-06
PGAP3	0	20.5951	Inf	3.07E-05	0.000432954
PGGHG	0	21.764	Inf	1.42E-06	3.37E-05
PGPEP1	2.02597	12.3058	2.602653734	4.23E-05	0.000571134
PGR	0	5.25723	Inf	2.38E-06	5.24E-05
PHF21A	0	8.04348	Inf	1.55E-05	0.000248612
PHF21B	0	14.4702	Inf	2.18E-05	0.000327135
PHF24	1.60803	12.8016	2.992957909	4.99E-05	0.000648193
PHF8	0	10.4904	Inf	5.57E-07	1.50E-05
PHLPP1	0	14.4561	Inf	2.00E-08	8.81E-07
PHYHD1	0	37.1151	Inf	2.82E-07	8.42E-06
PIDD1	0	18.7729	Inf	2.59E-05	0.000377427
PIGG	0	9.38969	Inf	1.10E-05	0.000187799
PIGO	0	19.2538	Inf	2.00E-07	6.31E-06
PIGZ	0	24.2363	Inf	3.64E-06	7.46E-05
PIK3AP1	0	14.3358	Inf	2.18E-06	4.85E-05
PIK3CD	0	13.8615	Inf	6.61E-07	1.74E-05
PIK3R5	0	12.8982	Inf	1.42E-05	0.000232345
PIK3R6	0	16.3326	Inf	1.84E-05	0.000285786
PIM3	0	24.8235	Inf	1.55E-05	0.000248612
PIP5K1C	0	16.5141	Inf	3.97E-09	2.13E-07
PITPNM1	2.30626	19.8904	3.108445217	1.61E-05	0.000257503
PITPNM2	2.10624	14.4701	2.78033315	5.98E-06	0.000113855
PITPNM3	0	17.1194	Inf	5.58E-11	4.48E-09

PITX1	0	22.973	Inf	3.64E-05	0.00050071
PITX2	0	18.5509	Inf	2.38E-07	7.25E-06
PKD1	2.0681	13.9032	2.749039124	1.10E-10	8.23E-09
PKD1L1	0	5.79497	Inf	5.57E-05	0.000705723
PKD1L2	0	13.6163	Inf	4.32E-10	2.86E-08
PKMYT1	2.18232	31.0796	3.832033367	2.85E-05	0.000409114
PKNOX2	0	15.8325	Inf	1.55E-05	0.000248612
PKP1	0	19.626	Inf	1.20E-09	7.31E-08
PLA2G4B	0	19.2283	Inf	5.57E-05	0.000705723
PLA2G4E	0	15.6946	Inf	6.61E-07	1.74E-05
PLA2G4F	0	18.3929	Inf	5.57E-06	0.000106758
PLAC4	0.973749	7.70903	2.984927496	5.38E-05	0.000688905
PLB1	0	10.4749	Inf	7.84E-06	0.000141315
PLCB3	2.55227	20.2436	2.987613011	4.17E-07	1.18E-05
PLCH2	0	18.0133	Inf	4.71E-11	3.84E-09
PLD2	0	19.2349	Inf	3.07E-06	6.49E-05
PLEC	1.77603	17.2811	3.282467196	1.33E-15	2.44E-13
PLEKHA6	1.97186	14.1283	2.840958852	2.87E-06	6.14E-05
PLEKHG2	0	11.8999	Inf	3.07E-08	1.29E-06
PLEKHG4B	0.423198	14.7493	5.123169866	1.58E-13	1.82E-11
PLEKHG5	0	18.1166	Inf	8.55E-10	5.35E-08
PLEKHH1	1.49025	11.7322	2.976847292	5.79E-05	0.00073122
PLEKHH3	0	18.1597	Inf	1.55E-05	0.000248612
PLEKHM1	1.83926	15.212	3.048012504	2.95E-05	0.000421596
PLEKHM3	0	6.85687	Inf	3.07E-06	6.49E-05
PLEKHN1	0	25.0863	Inf	1.01E-05	0.000174751
PLEKHO1	0	17.9672	Inf	8.53E-05	0.000998703
PLEKHO2	0	16.7976	Inf	7.84E-06	0.000141315
PLIN4	1.53412	19.3376	3.655925511	1.53E-08	7.01E-07
PLXNA1	0.537517	15.8331	4.880489558	2.42E-11	2.06E-09
PLXNA2	0.851648	9.41672	3.466895463	2.36E-07	7.25E-06
PLXNA3	0	18.6418	Inf	2.82E-11	2.39E-09
PLXNA4	0.316191	8.89418	4.813993393	8.32E-11	6.43E-09
PLXNB1	3.31325	17.7019	2.417585247	1.19E-06	2.91E-05
PLXNB3	0	18.4537	Inf	1.43E-10	1.05E-08
PML	2.52854	14.0909	2.478387255	8.14E-06	0.000146188
PNMA2	0	12.809	Inf	8.54E-06	0.000151469
PNMA3	0	16.893	Inf	6.61E-06	0.00012264
PNMA5	0	17.6736	Inf	1.10E-05	0.000187799
PNMAL2	0	19.4443	Inf	2.38E-08	1.03E-06
PNO1	1.50545	56.0631	5.218784822	1.82E-14	2.32E-12
PNPLA6	0	16.3748	Inf	1.55E-07	5.11E-06
PNPLA7	0	18.6063	Inf	3.34E-08	1.39E-06
PODN	0	21.0673	Inf	1.20E-06	2.91E-05
PODNL1	0	21.6427	Inf	3.64E-07	1.04E-05
POFUT1	0	11.0312	Inf	2.82E-06	6.03E-05
POFUT2	0	17.2495	Inf	1.31E-07	4.45E-06
POLE	0.608761	14.1084	4.534534638	8.19E-09	4.09E-07
POLH	0	8.66014	Inf	1.01E-06	2.52E-05
POLR1A	2.28812	11.132	2.282478193	7.51E-05	0.000907629
POLRMTP1	0	16.6997	Inf	8.54E-06	0.000151469

POM121	1.58751	13.6213	3.101026814	7.99E-08	2.93E-06
POM121L1C	0.510436	30.0708	5.880489356	1.55E-15	2.44E-13
POM121L2	0	16.7257	Inf	5.12E-05	0.000658307
POM121L8F	0	20.3351	Inf	6.22E-14	7.44E-12
POM121L9F	0	14.5227	Inf	5.57E-07	1.50E-05
POMT2	0	12.4701	Inf	1.10E-05	0.000187799
POTEC	0	22.3253	Inf	3.36E-13	3.73E-11
POTEM	0	37.118	Inf	1.33E-15	2.44E-13
POU2AF1	0	23.8846	Inf	1.10E-06	2.72E-05
POU2F2	0	10.799	Inf	3.96E-07	1.12E-05
POU6F1	0	11.6225	Inf	6.61E-06	0.00012264
PPAN	0	20.3556	Inf	7.20E-05	0.000872523
PPFIA3	1.02765	21.7257	4.401981765	5.34E-08	2.08E-06
PPFIA4	0	14.7058	Inf	1.20E-08	5.70E-07
PPL	3.906	18.5885	2.250646502	1.15E-05	0.000195933
PPM1F	2.81955	19.2044	2.767900059	6.92E-06	0.000127851
PPP1R12B	0	5.09921	Inf	8.54E-06	0.000151469
PPP1R13L	0	18.7181	Inf	1.42E-05	0.000232345
PPP1R16A	0	24.4951	Inf	2.82E-07	8.42E-06
PPP1R16B	0	10.8746	Inf	2.59E-06	5.60E-05
PPP1R18	6.35764	25.9629	2.029888307	2.71E-05	0.000394584
PPP1R26P1	0	23.3496	Inf	4.32E-08	1.73E-06
PPP1R3B	1.66347	19.2759	3.534530469	9.27E-08	3.31E-06
PPP1R9B	0	13.0947	Inf	5.12E-05	0.000658307
PPP2R2D	4.63842	22.0741	2.250649254	1.15E-05	0.000195933
PPT2-EGFL	0	22.1218	Inf	1.20E-05	0.000201663
PRAG1	0	15.088	Inf	1.42E-06	3.37E-05
PRDM11	0.859913	11.8281	3.78188383	1.97E-09	1.14E-07
PRDM12	0	21.9681	Inf	3.64E-05	0.00050071
PRDM16	0	11.5635	Inf	3.97E-09	2.13E-07
PRDM8	0	12.0533	Inf	7.84E-05	0.000933973
PRELP	0	11.1098	Inf	5.12E-06	9.95E-05
PREX1	0	15.9219	Inf	1.55E-09	9.13E-08
PRKAR1B	0	20.5611	Inf	1.01E-05	0.000174751
PRKAR2A-A	0	19.3902	Inf	3.07E-06	6.49E-05
PRKCG	0	16.9311	Inf	4.70E-05	0.000613083
PRNCR1	0	3.96431	Inf	8.53E-05	0.000998703
PROB1	0	28.6401	Inf	1.84E-08	8.19E-07
PROM2	3.04634	19.3119	2.664341237	2.22E-05	0.000331976
PRPF40B	0	14.8603	Inf	2.00E-05	0.000306049
PRR12	1.40133	13.1394	3.229030756	4.42E-06	8.82E-05
PRR36	0	17.9409	Inf	2.00E-07	6.31E-06
PRRT3	0	14.3687	Inf	2.38E-05	0.000350829
PRRT4	0	17.1451	Inf	1.10E-05	0.000187799
PRSS36	0	20.0422	Inf	2.38E-05	0.000350829
PRX	0	17.067	Inf	1.55E-08	7.06E-07
PSAPL1	0	15.8424	Inf	7.84E-07	2.02E-05
PSD	0	16.9384	Inf	2.59E-07	7.83E-06
PSD2	0	12.0429	Inf	3.96E-05	0.000536192
PSD4	3.86833	21.8995	2.501115152	6.14E-06	0.000115576
PSMG3-AS1	0	18.8407	Inf	2.82E-10	1.94E-08

PTAFR	0	16.1659	Inf	9.30E-07	2.34E-05
PTCH1	0	9.67598	Inf	6.07E-08	2.30E-06
PTCH2	0	19.3484	Inf	3.64E-08	1.49E-06
PTCHD4	0	29.5589	Inf	1.32E-13	1.53E-11
PTDSS2	0	23.6775	Inf	7.20E-06	0.000131598
PTGIS	0	9.69366	Inf	3.96E-05	0.000536192
PTK7	1.09607	18.3244	4.063354117	3.04E-06	6.47E-05
PTOV1	0	26.9834	Inf	6.61E-05	0.000810778
PTPN23	2.76777	17.1379	2.630394373	3.18E-05	0.000447066
PTPN3	0	5.76272	Inf	2.59E-05	0.000377427
PTPN7	0	21.508	Inf	2.00E-08	8.81E-07
PTPRH	0	14.1565	Inf	3.07E-05	0.000432954
PTPRN	0	21.0569	Inf	1.55E-07	5.11E-06
PTPRN2	0	10.374	Inf	4.31E-05	0.000573472
PTPRS	1.98984	13.6705	2.780341675	5.98E-06	0.000113855
PTPRT	0.383892	6.92739	4.173539496	9.09E-07	2.31E-05
PTPRU	0	16.8144	Inf	1.31E-08	6.13E-07
PYCRL	0	14.6164	Inf	7.84E-05	0.000933973
PYGB	2.3593	19.9304	3.078539882	2.18E-05	0.000327135
PYGM	0	15.1395	Inf	3.64E-05	0.00050071
QRICH2	0	10.5849	Inf	2.00E-05	0.000306049
QSOX2	0	11.7043	Inf	5.12E-05	0.000658307
RAB11FIP4	1.07563	12.5117	3.540024022	8.57E-08	3.09E-06
RAB36	0.951597	13.2996	3.804888322	3.62E-05	0.00050071
RAB3IP	2.38365	24.332	3.351610589	3.11E-15	4.50E-13
RAB44	0	16.2933	Inf	2.18E-06	4.85E-05
RAB4B-EGL	1.71047	22.8466	3.739514775	6.31E-05	0.000783683
RAD54L2	1.4446	9.0727	2.65086188	2.56E-05	0.000376121
RADIL	0	16.5612	Inf	1.10E-05	0.000187799
RAI1	3.81608	17.8342	2.22448318	1.92E-06	4.35E-05
RAMP2-AS1	0	24.2405	Inf	2.82E-07	8.42E-06
RANBP10	0.8282	15.6776	4.242581709	4.05E-07	1.15E-05
RANGAP1	2.04324	24.49	3.583262195	4.59E-08	1.82E-06
RAP1GAP2	0.72842	12.1779	4.063351027	3.04E-06	6.47E-05
RAPGEF1	0.751678	15.2264	4.340316294	1.20E-07	4.13E-06
RAPGEF3	2.21439	18.4124	3.055696452	1.57E-07	5.17E-06
RAPGEFL1	0	16.1486	Inf	5.12E-06	9.95E-05
RARG	0	18.9712	Inf	4.70E-06	9.28E-05
RASA3	0	13.3428	Inf	5.12E-06	9.95E-05
RASGRF1	0	11.6312	Inf	8.54E-08	3.08E-06
RASL10B	0	17.1398	Inf	3.64E-05	0.00050071
RASSF5	0	12.6235	Inf	5.57E-05	0.000705723
RAX	0	19.1462	Inf	1.01E-05	0.000174751
RBBP8NL	0	23.8886	Inf	2.82E-06	6.03E-05
RBM14	0	10.9855	Inf	1.42E-05	0.000232345
RBM19	2.58933	16.4911	2.671036886	2.06E-05	0.000313817
RBM20	0	11.0818	Inf	2.38E-07	7.25E-06
RBM33	1.88789	10.145	2.425922253	1.53E-05	0.000248596
RBM34	1.98256	30.339	3.935737101	1.10E-05	0.000187799
RBMS2	2.29702	12.9531	2.495462089	6.59E-06	0.00012264
RBP3	0	15.827	Inf	2.82E-06	6.03E-05

RD3	0	13.0349	Inf	3.07E-05	0.000432954
RELT	0	17.6305	Inf	5.57E-06	0.000106758
REXO1	0	15.3379	Inf	1.69E-06	3.89E-05
REXO1L2P	0	103.566	Inf	4.70E-10	3.09E-08
RFT1	0	12.1711	Inf	8.54E-06	0.000151469
RGMA	0	17.9295	Inf	1.20E-06	2.91E-05
RGS12	1.38756	12.7649	3.201560174	6.00E-06	0.000113855
RGS3	4.00147	16.046	2.003611704	3.75E-05	0.000515697
RGS6	0	13.3447	Inf	6.07E-08	2.30E-06
RGS8	0	11.8345	Inf	2.59E-06	5.60E-05
RHBDL3	0	18.9589	Inf	4.70E-08	1.85E-06
RHOBTB2	0	16.2119	Inf	1.43E-08	6.56E-07
RHOU	6.14518	80.2688	3.707312155	1.33E-15	2.44E-13
RHPN2P1	0	27.0192	Inf	2.82E-05	0.000404432
RIC3	0	7.05981	Inf	6.61E-05	0.000810778
RILPL1	0	12.3838	Inf	6.61E-05	0.000810778
RIMS1	0	7.75613	Inf	1.69E-06	3.89E-05
RIMS3	0	13.7744	Inf	5.12E-09	2.68E-07
RIMS4	0	13.4034	Inf	1.84E-06	4.18E-05
RIN1	0	20.236	Inf	3.96E-05	0.000536192
RIN3	0	15.3026	Inf	1.01E-05	0.000174751
RNF139-AS	0	9.47222	Inf	3.34E-05	0.000466589
RNF165	0	10.0417	Inf	1.55E-06	3.62E-05
RNF207	1.22718	17.0427	3.795735133	3.92E-05	0.000536192
RNF213	2.51917	10.0462	1.995629514	3.08E-09	1.69E-07
RNF222	0	16.8657	Inf	6.61E-05	0.000810778
RNF44	0	14.0123	Inf	1.84E-05	0.000285786
ROBO3	1.07008	17.9845	4.070963488	2.81E-06	6.03E-05
ROBO4	0	18.4242	Inf	1.55E-06	3.62E-05
RP1L1	0	23.2334	Inf	1.55E-15	2.44E-13
RPAP1	0	18.5016	Inf	6.61E-08	2.47E-06
RPH3A	0	10.2554	Inf	5.12E-05	0.000658307
RPL23AP53	0	14.4456	Inf	1.20E-07	4.12E-06
RPS6KA2	0.749251	10.6042	3.823042871	3.09E-05	0.000435638
RPS6KA4	0	16.6268	Inf	6.07E-05	0.000757821
RPS6KL1	0	11.4507	Inf	1.42E-05	0.000232345
RPTN	0	33.8181	Inf	7.85E-11	6.09E-09
RRP7A	1.78666	15.2511	3.09357628	1.87E-05	0.000290534
RSPO1	0	22.3275	Inf	2.18E-06	4.85E-05
RSRP1	2.0582	14.6863	2.835015891	3.09E-06	6.53E-05
RTEL1	0	18.5795	Inf	9.30E-09	4.55E-07
RTEL1-TNF	0	18.0729	Inf	2.00E-09	1.16E-07
RTEL1P1	0	11.7195	Inf	7.20E-05	0.000872523
RTKN	0	20.1232	Inf	2.18E-05	0.000327135
RTN4RL1	0	14.0317	Inf	7.84E-05	0.000933973
RTN4RL2	0	28.1548	Inf	7.84E-06	0.000141315
RUBCN	0.991683	11.8862	3.583264727	4.59E-08	1.82E-06
RUFY4	0	16.5303	Inf	9.30E-06	0.000162965
RUSC2	0.81545	13.8493	4.086072829	2.39E-06	5.25E-05
RXRA	1.33328	15.6267	3.550961451	7.33E-08	2.71E-06
RYR1	0	15.432	Inf	1.33E-15	2.44E-13

RYR3	0.625803	6.83651	3.449479546	2.98E-07	8.87E-06
S1PR2	0	17.4396	Inf	7.84E-06	0.000141315
SALL2	0	13.9763	Inf	8.54E-07	2.17E-05
SART1	1.35327	17.8361	3.720278578	7.39E-05	0.000893921
SATB2	0	7.1072	Inf	3.07E-05	0.000432954
SATL1	0	39.0598	Inf	1.10E-07	3.84E-06
SBF1	5.44484	22.4226	2.041992015	1.48E-07	4.94E-06
SBF1P1	0	13.3717	Inf	1.69E-07	5.47E-06
SBK1	0	17.7881	Inf	4.32E-08	1.73E-06
SCAF1	0	19.1893	Inf	1.42E-07	4.77E-06
SCAMP4	0	21.3126	Inf	2.18E-05	0.000327135
SCAMP5	0	14.2469	Inf	8.53E-05	0.000998703
SCARA5	0	12.1498	Inf	8.53E-05	0.000998703
SCARF1	0	14.4421	Inf	7.84E-05	0.000933973
SCART1	0	16.6266	Inf	1.84E-07	5.88E-06
SCIMP	0	12.9856	Inf	3.34E-05	0.000466589
SCN1B	0	14.3883	Inf	1.10E-07	3.84E-06
SCN2B	0	14.7131	Inf	1.10E-06	2.72E-05
SCN3B	0	9.02483	Inf	3.64E-05	0.00050071
SCN4A	1.24872	18.4464	3.884817364	3.18E-10	2.17E-08
SCN4B	0	13.0597	Inf	7.20E-06	0.000131598
SCN5A	0.566841	14.0895	4.635532492	1.73E-09	1.01E-07
SCNM1	0	39.0745	Inf	1.84E-07	5.88E-06
SCRIB	4.66954	20.4873	2.133377525	4.72E-05	0.000615287
SCRT1	0	14.3763	Inf	3.96E-05	0.000536192
SCRT2	0	15.0636	Inf	4.31E-05	0.000573472
SCUBE1	0	18.1651	Inf	1.69E-06	3.89E-05
SCUBE3	0	8.38312	Inf	5.12E-06	9.95E-05
SDC3	1.90319	19.1919	3.334006044	1.29E-06	3.13E-05
SDK1	0.458863	11.7304	4.676044918	9.00E-10	5.59E-08
SDK2	0	16.2823	Inf	3.33E-15	4.77E-13
SEC14L4	0	21.5018	Inf	3.96E-05	0.000536192
SEC14L5	0	11.0169	Inf	1.42E-06	3.37E-05
SEC31B	0	14.3001	Inf	3.96E-06	7.98E-05
SELENON	1.12491	17.0155	3.918968059	1.29E-05	0.000214835
SELPLG	0	29.1695	Inf	1.84E-07	5.88E-06
SEMA3B	0	16.1455	Inf	3.96E-05	0.000536192
SEMA3E	0	27.0333	Inf	1.78E-15	2.69E-13
SEMA3G	0	14.4698	Inf	1.20E-06	2.91E-05
SEMA4C	0	16.5045	Inf	1.69E-05	0.000266218
SEMA4F	0	13.1993	Inf	2.18E-07	6.76E-06
SEMA4G	0	13.0268	Inf	2.18E-05	0.000327135
SEMA5B	1.70777	17.6744	3.371475647	8.15E-07	2.10E-05
SEMA6B	0	21.3234	Inf	1.10E-07	3.84E-06
SEMA6C	0	18.3179	Inf	8.54E-07	2.17E-05
SEMA7A	0	16.0245	Inf	2.82E-05	0.000404432
9-Sep	5.33822	21.0719	1.980889736	2.14E-06	4.80E-05
SERPINB9	0	93.1488	Inf	1.33E-15	2.44E-13
SETBP1	0	7.42529	Inf	1.84E-07	5.88E-06
SETD1A	0.755407	13.2305	4.130469621	1.47E-06	3.49E-05
SEZ6	0	15.0637	Inf	4.70E-06	9.28E-05

SFI1	0	12.762	Inf	2.82E-05	0.000404432
SFXN5	0	20.9888	Inf	2.59E-10	1.81E-08
SGSM1	0	16.3302	Inf	4.70E-09	2.48E-07
SH2B1	0.874731	16.1715	4.20847027	6.07E-07	1.62E-05
SH2B3	1.74915	13.7704	2.976844548	5.79E-05	0.00073122
SH2D3C	2.29054	18.2352	2.992966361	4.99E-05	0.000648193
SH2D5	0	18.2138	Inf	1.84E-06	4.18E-05
SH3BP2	3.98213	15.0144	1.914734599	5.93E-06	0.000112999
SH3PXD2A	4.33282	13.606	1.650864786	2.62E-05	0.000382409
SH3PXD2B	0	15.5629	Inf	3.97E-11	3.29E-09
SH3RF3	0	16.8155	Inf	1.43E-08	6.56E-07
SH3RF3-AS	0	24.3851	Inf	2.59E-06	5.60E-05
SH3TC1	1.09953	21.0083	4.25600055	3.45E-07	9.99E-06
SHANK1	1.05788	13.6622	3.690941926	8.82E-09	4.35E-07
SHANK3	0	15.3928	Inf	8.55E-10	5.35E-08
SHC3	0	9.75269	Inf	1.20E-08	5.70E-07
SHF	0	16.8826	Inf	5.57E-05	0.000705723
SHISA5	6.98957	32.1502	2.201552104	2.12E-05	0.000321455
SHISA6	0	9.53389	Inf	1.55E-06	3.62E-05
SHISA7	0	15.4282	Inf	1.84E-08	8.19E-07
SHISA9	21.1683	642.431	4.923564193	1.55E-15	2.44E-13
SHROOM1	1.18481	26.9347	4.506738369	1.23E-08	5.82E-07
SHROOM2	0	12.263	Inf	1.55E-08	7.06E-07
SHROOM4	0	9.87367	Inf	1.43E-08	6.56E-07
SIDT1	0	10.1934	Inf	4.31E-05	0.000573472
SIGLEC1	0.725168	17.7042	4.60963258	2.61E-09	1.47E-07
SIM2	0	9.65244	Inf	8.53E-05	0.000998703
SIN3B	0.877251	14.0453	4.000953943	5.78E-06	0.000110263
SIPA1L3	3.04799	14.1817	2.218100408	1.73E-05	0.000272367
SIX4	0	10.3729	Inf	4.70E-06	9.28E-05
SKA1	0	28.3631	Inf	1.42E-07	4.77E-06
SKOR1	0	21.1644	Inf	3.34E-05	0.000466589
SLC11A1	0	20.9263	Inf	2.18E-07	6.76E-06
SLC12A3	0	9.60148	Inf	4.70E-05	0.000613083
SLC12A4	0.969584	19.1258	4.302010393	1.96E-07	6.23E-06
SLC12A5	0	15.71	Inf	6.61E-09	3.35E-07
SLC12A7	1.83753	14.7913	3.008909147	4.29E-05	0.000573472
SLC12A9	0	19.3355	Inf	2.18E-06	4.85E-05
SLC14A2	0	14.6678	Inf	4.32E-06	8.63E-05
SLC18A3	0	20.8405	Inf	8.53E-05	0.000998703
SLC1A4	0	11.0089	Inf	5.57E-05	0.000705723
SLC1A5	3.04001	24.3362	3.00095594	4.63E-05	0.000611824
SLC1A6	0	12.9066	Inf	5.12E-05	0.000658307
SLC1A7	0	16.0495	Inf	2.00E-05	0.000306049
SLC22A11	0	22.4129	Inf	6.07E-08	2.30E-06
SLC22A12	0	18.66	Inf	1.31E-05	0.000216387
SLC22A13	0	22.2265	Inf	2.38E-05	0.000350829
SLC22A23	0.681938	11.2802	4.048008258	3.57E-06	7.37E-05
SLC24A4	0	10.3039	Inf	3.34E-09	1.83E-07
SLC25A22	0	20.533	Inf	3.64E-06	7.46E-05
SLC25A23	0	20.4317	Inf	1.55E-06	3.62E-05

SLC25A25-	1.26838	23.0003	4.18059374	8.39E-07	2.16E-05
SLC25A45	0	17.4286	Inf	7.20E-06	0.000131598
SLC26A1	0	19.2588	Inf	3.07E-07	9.07E-06
SLC26A11	0	17.2255	Inf	5.57E-05	0.000705723
SLC26A9	0	14.5513	Inf	1.84E-06	4.18E-05
SLC27A4	0	16.0501	Inf	7.20E-05	0.000872523
SLC28A2	0	21.5267	Inf	5.12E-05	0.000658307
SLC29A2	0	18.6445	Inf	4.31E-05	0.000573472
SLC2A10	0	12.8219	Inf	3.96E-05	0.000536192
SLC2A11	0	17.2864	Inf	2.82E-06	6.03E-05
SLC30A3	0	24.8784	Inf	5.12E-06	9.95E-05
SLC32A1	0	22.943	Inf	1.55E-05	0.000248612
SLC34A1	0	21.4222	Inf	7.84E-07	2.02E-05
SLC35C1	0	17.203	Inf	2.82E-06	6.03E-05
SLC35E4	1.81562	15.4179	3.086072085	2.02E-05	0.000308151
SLC35F6	0	13.6635	Inf	4.70E-05	0.000613083
SLC38A7	0	17.6402	Inf	2.00E-07	6.31E-06
SLC39A3	0	18.9981	Inf	6.61E-07	1.74E-05
SLC39A4	0	19.5936	Inf	6.61E-05	0.000810778
SLC43A2	0	32.7177	Inf	1.33E-15	2.44E-13
SLC44A4	0	19.1722	Inf	5.57E-05	0.000705723
SLC45A4	0	12.8805	Inf	3.34E-09	1.83E-07
SLC47A2	0	16.5292	Inf	3.64E-05	0.00050071
SLC4A1	0	17.1449	Inf	9.30E-08	3.31E-06
SLC4A2	3.37591	17.9173	2.408004902	1.89E-05	0.000292332
SLC4A3	0	20.427	Inf	4.70E-08	1.85E-06
SLC4A5	0	9.03096	Inf	2.00E-05	0.000306049
SLC4A8	1.47648	7.77096	2.395931021	2.17E-05	0.000327135
SLC52A1	0	23.2041	Inf	2.82E-05	0.000404432
SLC6A10P	0	52.4368	Inf	1.33E-15	2.44E-13
SLC6A11	0	8.74087	Inf	1.69E-11	1.47E-09
SLC6A12	1.22718	16.0657	3.710565061	8.00E-05	0.000951668
SLC6A17	0	19.1313	Inf	5.58E-11	4.48E-09
SLC6A19	0	11.1639	Inf	2.00E-05	0.000306049
SLC6A2	0	11.7158	Inf	7.84E-06	0.000141315
SLC6A3	0	13.9158	Inf	3.64E-05	0.00050071
SLC6A4	0	10.571	Inf	1.84E-06	4.18E-05
SLC6A7	0	14.3874	Inf	3.34E-05	0.000466589
SLC6A9	0	22.0849	Inf	1.55E-07	5.11E-06
SLC7A1	0	9.86219	Inf	1.10E-06	2.72E-05
SLC7A5P2	3.81907	48.6463	3.67103681	1.21E-08	5.73E-07
SLC8A2	0.953459	16.9523	4.152166312	1.16E-06	2.84E-05
SLC8A3	0	9.03786	Inf	7.84E-05	0.000933973
SLC9A1	2.0553	15.9079	2.952322502	7.25E-05	0.000878344
SLC9A3	0	14.4045	Inf	2.00E-05	0.000306049
SLC9A4	4.71061	29.4804	2.645770301	8.97E-07	2.28E-05
SLC9A5	0	14.0155	Inf	3.64E-05	0.00050071
SLC9A8	0.770943	11.1158	3.849843774	2.43E-05	0.000358952
SLCO2A1	0	13.2697	Inf	2.82E-05	0.000404432
SLFNL1	0	25.4982	Inf	3.64E-05	0.00050071
SLFNL1-AS	0	19.3079	Inf	2.00E-08	8.81E-07

SLIT1	0	14.5661	Inf	2.18E-10	1.53E-08
SLIT2	0	8.07068	Inf	4.70E-06	9.28E-05
SLIT3	0	13.3295	Inf	1.31E-11	1.15E-09
SLX4	1.33237	17.9142	3.749036938	3.42E-09	1.87E-07
SMG1P7	0	59.4958	Inf	3.97E-10	2.65E-08
SMG6	0	13.956	Inf	5.58E-10	3.61E-08
SMIM10L2A	0	16.2881	Inf	8.54E-08	3.08E-06
SMIM17	0	51.89	Inf	8.55E-12	7.74E-10
SMIM5	0	16.5047	Inf	1.01E-07	3.58E-06
SMPD3	0	16.1985	Inf	8.54E-08	3.08E-06
SMTN	1.26575	19.7056	3.960541288	8.62E-06	0.000152845
SNAPC4	1.04618	19.7114	4.235827247	4.40E-07	1.23E-05
SNED1	0.713071	10.4706	3.876154574	1.92E-05	0.000296702
SNX20	0	14.3087	Inf	1.55E-07	5.11E-06
SOBP	0	8.57693	Inf	4.70E-05	0.000613083
SOCS7	2.44328	13.5078	2.466901613	9.37E-06	0.00016398
SOD2	19.7468	43.3407	1.134103574	9.68E-07	2.43E-05
SOGA1	0	11.4605	Inf	7.99E-15	1.09E-12
SOGA3	0	5.72655	Inf	2.82E-06	6.03E-05
SOHLH1	0	28.6361	Inf	2.38E-05	0.000350829
SORCS2	0	13.4595	Inf	1.84E-07	5.88E-06
SOX11	0	7.46531	Inf	4.70E-06	9.28E-05
SOX12	0	15.7321	Inf	9.30E-07	2.34E-05
SOX13	2.39289	20.5317	3.101027102	1.74E-05	0.000273494
SPACA9	0	21.6622	Inf	7.20E-05	0.000872523
SPATA20	0	20.6673	Inf	2.18E-05	0.000327135
SPATA31E1	0	23.9424	Inf	1.31E-09	7.85E-08
SPC25	3.62045	151.8	5.389858954	1.78E-15	2.69E-13
SPDYE1	0	21.1463	Inf	2.82E-05	0.000404432
SPEG	0	18.1444	Inf	1.33E-15	2.44E-13
SPIB	0	17.3352	Inf	1.01E-05	0.000174751
SPN	0.692009	11.9365	4.108445263	1.88E-06	4.26E-05
SPOCD1	0	23.8269	Inf	1.43E-08	6.56E-07
SPOCK2	1.55815	16.3327	3.389857276	6.46E-07	1.72E-05
SPON2	0	21.3964	Inf	5.12E-05	0.000658307
SPOUT1	1.13699	16.293	3.840960798	2.63E-05	0.000383981
SPPL2B	0	21.1459	Inf	2.18E-07	6.76E-06
SPPL3	0	13.0498	Inf	4.31E-05	0.000573472
SPRED3	0	12.456	Inf	5.12E-06	9.95E-05
SPRNP1	0	22.8898	Inf	7.20E-05	0.000872523
SPTB	0.937952	13.9386	3.893427759	2.71E-10	1.88E-08
SPTBN2	1.23888	16.4928	3.73472799	4.34E-09	2.31E-07
SPTBN4	0.533223	14.9991	4.813993115	8.32E-11	6.43E-09
SPTBN5	0.41569	19.8193	5.575254156	1.55E-15	2.44E-13
SRC	0	18.702	Inf	2.82E-07	8.42E-06
SRCAP	3.25682	15.2686	2.229031894	2.16E-07	6.76E-06
SRCIN1	1.38245	20.9721	3.923172123	1.55E-10	1.13E-08
SREBF2	0	14.7511	Inf	8.54E-08	3.08E-06
SRF	3.42615	22.0227	2.684331135	1.78E-05	0.000279949
SRGAP3	0	7.81305	Inf	1.69E-06	3.89E-05
SRL	0	11.3754	Inf	4.70E-06	9.28E-05

SRRM3	0	18.286	Inf	2.82E-06	6.03E-05
SRRM4	0.574865	8.39034	3.867434172	2.07E-05	0.000315709
SRRM5	2.03216	36.4909	4.166450833	9.86E-07	2.47E-05
SSC5D	0	19.1994	Inf	6.61E-09	3.35E-07
SSPO	0.625201	22.2595	5.153957291	1.55E-15	2.44E-13
SSTR1	0	14.1644	Inf	1.10E-05	0.000187799
SSTR3	0	18.8507	Inf	2.00E-07	6.31E-06
SSTR5	0	19.6657	Inf	4.70E-05	0.000613083
ST14	0	19.3324	Inf	6.07E-06	0.000114316
ST3GAL1	1.39811	10.945	2.968722176	6.24E-05	0.000777378
ST3GAL2	0	22.3764	Inf	5.12E-09	2.68E-07
ST5	0	10.8109	Inf	7.84E-05	0.000933973
ST6GALNAC	0	20.1712	Inf	3.64E-05	0.00050071
STAB1	1.84448	16.6964	3.178251018	2.38E-08	1.03E-06
STAG3L5P-	0	13.1202	Inf	8.53E-05	0.000998703
STARD9	0.625401	10.1514	4.02075337	2.28E-11	1.94E-09
STK10	0	12.5329	Inf	6.07E-07	1.62E-05
STK11IP	0	18.4366	Inf	3.34E-06	6.95E-05
STK35	0	10.0809	Inf	5.12E-06	9.95E-05
STK36	0	10.4817	Inf	6.61E-05	0.000810778
STRA6	0	17.7524	Inf	1.31E-08	6.13E-07
STS	0	8.33794	Inf	7.20E-06	0.000131598
STUM	1.89739	13.8745	2.870347628	1.98E-06	4.49E-05
STX1B	0	16.5083	Inf	4.32E-07	1.21E-05
SUFU	1.82309	14.1913	2.960549065	6.73E-05	0.000824237
SV2A	0	15.5337	Inf	1.55E-06	3.62E-05
SV2B	0	5.83884	Inf	1.69E-07	5.47E-06
SVOP	0	10.8447	Inf	2.38E-06	5.24E-05
SYDE1	1.47536	19.9674	3.758507556	5.38E-05	0.000688952
SYNE1	1.38402	4.8052	1.795731688	3.18E-05	0.000447668
SYNE3	0.721785	7.88505	3.449478836	2.98E-07	8.87E-06
SYNGAP1	0.810837	12.4082	3.935738112	1.10E-05	0.000187799
SYNGR1	0	12.4723	Inf	3.34E-06	6.95E-05
SYNM	0	10.1433	Inf	7.20E-07	1.87E-05
SYNPO	1.18122	18.1284	3.939902005	1.13E-10	8.43E-09
SYNPO2L	0	18.3917	Inf	1.69E-08	7.60E-07
SYPL2	0	13.8021	Inf	6.07E-05	0.000757821
SYT12	0	20.1902	Inf	3.96E-07	1.12E-05
SYT15	0.855986	21.5038	4.650860614	1.36E-09	8.12E-08
SYT2	0	10.8042	Inf	1.10E-07	3.84E-06
SYT7	0	17.8838	Inf	1.69E-10	1.23E-08
SZT2	3.13743	13.4754	2.102672922	4.57E-08	1.82E-06
TAF1	0	6.02637	Inf	1.42E-05	0.000232345
TAF1C	1.15972	22.2608	4.262657009	3.18E-07	9.39E-06
TANGO2	0	16.6837	Inf	7.84E-07	2.02E-05
TAOK2	2.97097	16.3594	2.461113894	1.01E-05	0.000174751
TAP2	0.783713	10.5373	3.74903601	5.83E-05	0.000734196
TAS1R3	0	15.3995	Inf	5.57E-05	0.000705723
TATDN3	1.86781	24.4526	3.710568261	8.00E-05	0.000951668
TBC1D16	1.72454	13.8055	3.000959643	2.72E-09	1.53E-07
TBX2	0	19.1299	Inf	5.12E-06	9.95E-05

TBX5	0	11.5621	Inf	8.53E-05	0.000998703
TCHH	1.39332	25.1426	4.173535306	8.50E-13	8.97E-11
TCHHL1	0	17.228	Inf	8.54E-06	0.000151469
TCOF1	0	17.113	Inf	2.82E-09	1.57E-07
TECTA	0	7.86411	Inf	7.84E-05	0.000933973
TEF	0	13.4966	Inf	7.20E-06	0.000131598
TEKT4P2	6.69616	35.8353	2.419975527	1.64E-05	0.000261826
TENM2	0	10.0721	Inf	4.70E-09	2.48E-07
TENM4	0.359694	9.99061	4.795730768	1.16E-10	8.63E-09
TERT	1.21282	19.4181	4.00096469	5.78E-06	0.000110263
TET3	2.13959	11.8856	2.473808463	5.15E-07	1.41E-05
TFCP2L1	0	14.528	Inf	4.71E-12	4.49E-10
TFDP2	0.961074	7.77872	3.016813353	3.98E-05	0.000538538
TG	0.576497	9.33207	4.016812124	4.92E-06	9.71E-05
TGOLN2	0	14.2926	Inf	2.38E-08	1.03E-06
THBD	0	15.9295	Inf	5.57E-06	0.000106758
THY1	0	17.9564	Inf	1.10E-06	2.72E-05
TIAM1	0	7.96485	Inf	2.18E-05	0.000327135
TIE1	0	15.6966	Inf	7.84E-06	0.000141315
TIMM50	0.891046	12.9263	3.858665657	2.25E-05	0.000336458
TINCR	0	18.0138	Inf	3.07E-06	6.49E-05
TJAP1	0	17.2756	Inf	4.31E-05	0.000573472
TKFC	1.14905	15.856	3.786515385	4.24E-05	0.000571998
TLCD2	0	20.4536	Inf	7.85E-11	6.09E-09
TLE3	0	14.8038	Inf	5.58E-08	2.15E-06
TLL2	0	7.44853	Inf	8.53E-05	0.000998703
TLR9	0	20.829	Inf	2.18E-07	6.76E-06
TLX2	0	23.8365	Inf	7.20E-05	0.000872523
TMC8	1.10277	20.1922	4.194594274	7.14E-07	1.87E-05
TMCC2	0	14.2365	Inf	2.59E-06	5.60E-05
TMEM104	0	15.1584	Inf	1.20E-06	2.91E-05
TMEM105	0	22.5849	Inf	6.61E-07	1.74E-05
TMEM120B	2.59554	17.736	2.772574049	1.36E-07	4.63E-06
TMEM129	0	17.9292	Inf	7.84E-05	0.000933973
TMEM132B	0	6.53062	Inf	1.10E-06	2.72E-05
TMEM132D	0	13.8787	Inf	2.38E-07	7.25E-06
TMEM132E	0	19.4205	Inf	3.34E-08	1.39E-06
TMEM147-A	0	30.9982	Inf	8.53E-05	0.000998703
TMEM151B	0	16.7982	Inf	3.96E-07	1.12E-05
TMEM170A	0	11.8157	Inf	1.31E-05	0.000216387
TMEM179	0	26.4396	Inf	9.30E-09	4.55E-07
TMEM184A	0	15.7286	Inf	6.07E-09	3.12E-07
TMEM184B	0	20.2302	Inf	3.34E-07	9.75E-06
TMEM198	0	21.2353	Inf	3.64E-05	0.00050071
TMEM201	0	16.0116	Inf	1.84E-09	1.07E-07
TMEM212	2.59071	48.3539	4.222212819	5.17E-07	1.41E-05
TMEM229B	0	16.0005	Inf	4.70E-06	9.28E-05
TMEM41B	3.53125	33.2145	3.233562329	9.54E-09	4.66E-07
TMEM51-AS	0	9.57362	Inf	3.34E-06	6.95E-05
TMEM63C	0	17.7148	Inf	1.10E-08	5.30E-07
TMEM86A	0	15.1125	Inf	3.34E-05	0.000466589

TMEM8B	0	14.4763	Inf	1.31E-05	0.000216387
TMEM92	0	19.5936	Inf	3.34E-05	0.000466589
TMEM94	0	16.3096	Inf	1.20E-08	5.70E-07
TMPPE	0	12.7006	Inf	8.53E-05	0.000998703
TMPRSS9	0	17.1655	Inf	2.00E-05	0.000306049
TNC	0	8.06984	Inf	2.00E-06	4.49E-05
TNFAIP8L2	0	41.6013	Inf	2.00E-07	6.31E-06
TNFRSF1B	1.32602	20.644	3.960548095	8.62E-06	0.000152845
TNFRSF8	0	13.7995	Inf	7.84E-05	0.000933973
TNFSF14	0	16.9609	Inf	2.00E-07	6.31E-06
TNIP1	0	23.5249	Inf	9.30E-07	2.34E-05
TNK1	0	21.6783	Inf	6.61E-06	0.00012264
TNK2-AS1	0	16.3893	Inf	3.07E-06	6.49E-05
TNKS1BP1	3.34923	18.5163	2.466894484	9.37E-06	0.00016398
TNNI1	0	13.8237	Inf	9.30E-08	3.31E-06
TNR	0	10.382	Inf	4.31E-05	0.000573472
TNRC18	7.37515	18.0628	1.292277252	6.91E-05	0.00084593
TNS1	1.42267	18.625	3.710567465	5.83E-13	6.30E-11
TNS4	0	13.4442	Inf	3.64E-05	0.00050071
TNXB	0.349905	13.3091	5.249305925	9.55E-15	1.29E-12
TOM1L2	0	17.4764	Inf	2.59E-09	1.46E-07
TOMM40	0	27.9878	Inf	8.53E-05	0.000998703
TONSL	2.15864	23.1999	3.425924019	4.06E-07	1.15E-05
TOR2A	0	21.6126	Inf	3.64E-05	0.00050071
TOR4A	0	22.8825	Inf	1.84E-08	8.19E-07
TP53I11	0	17.2209	Inf	1.42E-07	4.77E-06
TP73	0	17.0176	Inf	1.55E-08	7.06E-07
TP73-AS1	1.50988	13.4894	3.159320381	9.47E-06	0.000165243
TPCN2	0	13.7226	Inf	2.18E-06	4.85E-05
TPPP	0	15.2467	Inf	2.38E-08	1.03E-06
TPRN	0	20.316	Inf	4.70E-05	0.000613083
TRAF3	0.626688	9.53476	3.927377351	1.19E-05	0.000201626
TRAF3IP2	0	12.2913	Inf	2.59E-07	7.83E-06
TRAM2	0	10.9338	Inf	4.32E-07	1.21E-05
TRANK1	1.37711	10.0294	2.864519593	2.14E-06	4.79E-05
TREM1	0	11.0914	Inf	1.20E-05	0.000201663
TREML2	0	14.0943	Inf	4.31E-05	0.000573472
TRERF1	0	6.59786	Inf	8.53E-05	0.000998703
TRH	0	27.3689	Inf	6.61E-05	0.000810778
TRHDE-AS1	0	9.43614	Inf	2.38E-05	0.000350829
TRIL	0	14.5876	Inf	8.54E-07	2.17E-05
TRIM26	0	14.7034	Inf	5.12E-05	0.000658307
TRIM41	0	16.9458	Inf	8.54E-06	0.000151469
TRIM56	0	13.964	Inf	3.96E-06	7.98E-05
TRIM66	0.499654	11.7124	4.550963516	6.41E-09	3.28E-07
TRIM67	0	8.75069	Inf	7.20E-07	1.87E-05
TRIM9	0	9.66782	Inf	3.34E-06	6.95E-05
TRIOBP	3.13817	20.6211	2.71612588	6.33E-12	5.84E-10
TRMT61A	0	23.8074	Inf	4.32E-07	1.21E-05
TRO	0	10.902	Inf	3.96E-05	0.000536192
TRPM2	0	14.881	Inf	2.38E-08	1.03E-06

TRPM4	3.55357	20.851	2.552775552	6.99E-05	0.000853216
TRPM5	0	19.1997	Inf	6.07E-07	1.62E-05
TRPV1	0	18.4578	Inf	2.59E-08	1.11E-06
TRPV4	0	17.1679	Inf	3.07E-05	0.000432954
TSC2	5.03596	19.7489	1.971433569	5.52E-05	0.000705723
TSC22D2	1.94964	14.9177	2.935745458	8.42E-05	0.000997309
TSHZ2	0	7.02387	Inf	4.70E-08	1.85E-06
TSHZ3	0	11.454	Inf	4.70E-08	1.85E-06
TSIX	0.789661	4.47044	2.50111149	1.89E-08	8.41E-07
TSNARE1	1.15889	16.9144	3.867436462	2.07E-05	0.000315709
TSNAX-DIS	0	6.70704	Inf	4.32E-06	8.63E-05
TSPAN11	0	16.6627	Inf	2.00E-08	8.81E-07
TSPAN14	0	14.4485	Inf	2.18E-07	6.76E-06
TSPAN18	0	14.2499	Inf	1.20E-05	0.000201663
TSPAN9	3.36001	19.7152	2.552770914	6.99E-05	0.000853216
TSPEAR	0	13.4807	Inf	3.96E-05	0.000536192
TSPOAP1	0	24.6944	Inf	1.55E-15	2.44E-13
TSPYL5	0	16.999	Inf	5.57E-07	1.50E-05
TTBK1	0	17.5359	Inf	6.62E-11	5.25E-09
TTC16	0	18.4379	Inf	4.70E-05	0.000613083
TTC28	0	7.41884	Inf	5.58E-08	2.15E-06
TTC28-AS1	0	12.5942	Inf	7.84E-07	2.02E-05
TTC7A	1.68825	16.0537	3.24930538	3.51E-06	7.27E-05
TTLL10	0	17.4903	Inf	3.64E-05	0.00050071
TTLL3	0	20.444	Inf	2.59E-08	1.11E-06
TTN	0.542112	2.55628	2.237383022	5.79E-13	6.28E-11
TUB	0.738129	10.6426	3.849833863	2.43E-05	0.000358952
TUBGCP6	1.61925	14.0368	3.11581643	1.49E-05	0.000242721
TUSC5	0	19.4193	Inf	1.84E-06	4.18E-05
TYK2	3.44147	20.5991	2.581484467	5.25E-05	0.000674957
TYRO3	2.26868	18.1615	3.000958328	4.63E-05	0.000611824
UBE2O	0	16.0993	Inf	7.20E-08	2.67E-06
UBE2S	4.09163	55.3755	3.758500225	5.38E-05	0.000688952
UBIAD1	0	14.1898	Inf	1.20E-10	8.95E-09
UCK2	0	13.4925	Inf	3.64E-06	7.46E-05
UCKL1	0	22.8264	Inf	6.07E-05	0.000757821
UCKL1-AS1	0	49.7837	Inf	2.00E-15	3.00E-13
UGDH-AS1	82.1221	5649.54	6.104219074	1.55E-15	2.44E-13
UGGT1	2.22274	9.75213	2.133378156	4.72E-05	0.000615287
UGT8	3.65165	537.412	7.201336232	1.55E-15	2.44E-13
ULBP3	0	24.9532	Inf	3.34E-07	9.75E-06
ULK2	0	6.08455	Inf	3.07E-05	0.000432954
UMODL1	0	11.4054	Inf	7.20E-06	0.000131598
UNC13A	0	13.6267	Inf	5.58E-12	5.20E-10
UNC5A	0	17.6673	Inf	3.96E-06	7.98E-05
UNC5B	0	17.33	Inf	1.01E-10	7.68E-09
UNC5C	0	7.15597	Inf	1.55E-06	3.62E-05
UNK	0	11.193	Inf	2.59E-05	0.000377427
UNKL	0.7797	12.4145	3.992963213	6.26E-06	0.000117596
URB1	1.80353	11.8454	2.715431591	3.26E-07	9.61E-06
UROC1	0	16.5167	Inf	2.38E-05	0.000350829

USH1G	0	19.6925	Inf	1.69E-06	3.89E-05
USP13	0	6.3575	Inf	8.53E-05	0.000998703
USP19	2.73106	16.1054	2.560011578	6.50E-05	0.000806613
USP2	0	15.8984	Inf	5.12E-06	9.95E-05
USP35	0	20.5981	Inf	4.32E-08	1.73E-06
USP36	1.64605	12.6675	2.944051764	7.81E-05	0.000933973
USP4	0	10.8005	Inf	4.70E-05	0.000613083
USP6	0.493881	7.60154	3.944056351	1.01E-05	0.000174751
VANGL2	0	10.8731	Inf	1.84E-05	0.000285786
VARS	4.53948	23.6912	2.383752325	2.49E-05	0.000367051
VASH1	0	15.012	Inf	3.07E-08	1.29E-06
VAV2	0	17.15	Inf	1.31E-07	4.45E-06
VAX1	0	10.352	Inf	1.31E-05	0.000216387
VGF	0	23.3005	Inf	1.42E-05	0.000232345
VIPR2	0	12.6128	Inf	1.69E-05	0.000266218
VRTN	0	20.3163	Inf	2.00E-06	4.49E-05
VSIG10L	0	15.7218	Inf	2.00E-05	0.000306049
VSIR	0	21.3995	Inf	3.07E-09	1.69E-07
VSTM4	1.20265	11.9148	3.308465985	1.76E-06	4.04E-05
VWA1	1.04596	14.0633	3.749035582	5.83E-05	0.000734196
VWA5B1	0	16.4718	Inf	8.54E-07	2.17E-05
VWA5B2	0	18.7462	Inf	3.64E-07	1.04E-05
VWA7	0	17.8992	Inf	6.07E-05	0.000757821
VWCE	0	21.5774	Inf	5.12E-07	1.40E-05
VWF	1.10277	12.1446	3.461111143	2.55E-07	7.75E-06
WASH3P	0	32.743	Inf	1.20E-07	4.12E-06
WASH5P	4.28595	83.7856	4.289015389	2.30E-07	7.11E-06
WBSCR17	2.7704	23.7708	3.10102426	1.74E-05	0.000273494
WDFY4	1.46004	11.0639	2.921780222	1.02E-06	2.54E-05
WDR81	4.04577	18.7288	2.210772255	2.35E-06	5.19E-05
WDR87	0	12.1712	Inf	5.58E-10	3.61E-08
WDR90	0.879467	16.9592	4.269294862	2.93E-07	8.75E-06
WFIKKN2	0	17.5879	Inf	1.01E-06	2.52E-05
WIZ	0	14.0373	Inf	2.00E-06	4.49E-05
WNK2	0	13.78	Inf	1.84E-10	1.32E-08
WNK4	0	12.1745	Inf	7.84E-05	0.000933973
WNT3A	0	18.8552	Inf	2.82E-05	0.000404432
WNT7B	0	20.2644	Inf	2.82E-07	8.42E-06
WNT9B	0	13.5797	Inf	3.07E-08	1.29E-06
WSCD1	0	14.144	Inf	1.55E-07	5.11E-06
WSCD2	0	17.2058	Inf	3.96E-08	1.62E-06
WT1-AS	0	12.4479	Inf	4.70E-05	0.000613083
WTIP	0	23.0785	Inf	7.84E-05	0.000933973
XIRP1	0	19.4784	Inf	2.82E-11	2.39E-09
XKR5	0	11.9096	Inf	1.31E-05	0.000216387
XKR8	0	18.8949	Inf	7.84E-05	0.000933973
XKR9	0	18.1192	Inf	9.30E-06	0.000162965
XRCC3	0	19.4377	Inf	7.20E-05	0.000872523
XRRA1	0	10.3798	Inf	6.07E-05	0.000757821
XYLT1	1.04394	11.7738	3.495469325	1.60E-07	5.24E-06
XYLT2	0	18.6741	Inf	3.07E-06	6.49E-05

YIF1B	6.60315	37.2359	2.495467904	6.59E-06	0.00012264
ZAN	0	14.6599	Inf	1.84E-11	1.60E-09
ZBTB32	0	24.1295	Inf	7.84E-05	0.000933973
ZBTB39	0	9.32881	Inf	1.84E-05	0.000285786
ZBTB4	3.18922	16.5738	2.377628891	2.67E-05	0.000388676
ZBTB42	0	16.9641	Inf	1.31E-05	0.000216387
ZBTB46	0	11.5323	Inf	1.20E-05	0.000201663
ZBTB47	0.884897	13.8547	3.968722123	7.96E-06	0.000143202
ZBTB7C	0	17.9647	Inf	1.20E-07	4.12E-06
ZBTB8A	0	7.95659	Inf	1.69E-05	0.000266218
ZC3H12D	0	13.089	Inf	5.57E-06	0.000106758
ZC3H18	0	14.7507	Inf	2.82E-05	0.000404432
ZCCHC24	0	10.3112	Inf	7.84E-05	0.000933973
ZDHHC18	0	16.2042	Inf	7.84E-05	0.000933973
ZDHHC22	0	17.5814	Inf	1.31E-05	0.000216387
ZDHHC3	1.14294	8.82947	2.949577171	7.03E-07	1.84E-05
ZDHHC8	0.972292	16.2551	4.06335897	3.04E-06	6.47E-05
ZFHX2	0	14.6472	Inf	5.13E-12	4.80E-10
ZFHX3	2.10958	10.6099	2.330383355	3.45E-08	1.43E-06
ZFHX4	0	6.54712	Inf	2.59E-08	1.11E-06
ZFP41	0	18.7267	Inf	1.84E-10	1.32E-08
ZFR2	0	14.9083	Inf	8.54E-08	3.08E-06
ZFYVE28	0.831025	16.1721	4.282471341	2.49E-07	7.59E-06
ZHX3	0.975504	8.80154	3.17353628	8.13E-06	0.000146158
ZNF142	0.781702	10.7177	3.7772328	4.59E-05	0.000609123
ZNF154	0	8.04263	Inf	3.07E-05	0.000432954
ZNF2	0	15.8936	Inf	6.07E-06	0.000114316
ZNF264	2.40391	9.28535	1.949573408	7.13E-05	0.000870537
ZNF316	2.01953	11.9689	2.567199085	6.06E-05	0.000757821
ZNF319	0	12.8675	Inf	3.96E-05	0.000536192
ZNF341	0	16.6209	Inf	1.42E-05	0.000232345
ZNF366	0	10.0246	Inf	7.20E-06	0.000131598
ZNF408	0	20.8824	Inf	5.12E-05	0.000658307
ZNF423	0.61128	11.2469	4.201550228	6.58E-07	1.74E-05
ZNF426	1.32928	61.0845	5.522089416	1.55E-15	2.44E-13
ZNF445	0	8.49586	Inf	6.07E-08	2.30E-06
ZNF454	0	38.8876	Inf	1.33E-15	2.44E-13
ZNF469	0.738185	18.9689	4.683509787	1.55E-15	2.44E-13
ZNF471	0	8.16817	Inf	7.20E-05	0.000872523
ZNF488	0	14.4531	Inf	1.55E-05	0.000248612
ZNF490	0.799135	17.4601	4.44947877	2.78E-08	1.19E-06
ZNF496	1.4512	14.0563	3.275898622	2.58E-06	5.60E-05
ZNF497	0	17.9104	Inf	6.07E-06	0.000114316
ZNF500	0	16.4808	Inf	1.43E-08	6.56E-07
ZNF503	0	15.0492	Inf	3.07E-05	0.000432954
ZNF503-AS	2.91891	23.8832	3.032494516	3.43E-05	0.00047778
ZNF512B	1.64577	17.8334	3.437747149	3.48E-07	1.01E-05
ZNF516	0	10.3942	Inf	3.64E-08	1.49E-06
ZNF517	2.51711	19.8163	2.976847445	5.79E-05	0.00073122
ZNF541	0	11.953	Inf	3.64E-05	0.00050071
ZNF543	0	26.4202	Inf	8.54E-09	4.24E-07

ZNF544	0	7.04993	Inf	1.31E-05	0.000216387
ZNF555	0	6.90947	Inf	1.55E-05	0.000248612
ZNF592	1.78743	11.542	2.690934587	1.66E-05	0.000264306
ZNF608	1.72653	14.6614	3.086075564	2.02E-05	0.000308151
ZNF618	1.03113	12.7238	3.625231463	2.45E-08	1.06E-06
ZNF646	0	21.0392	Inf	1.10E-11	9.81E-10
ZNF687	0	15.7344	Inf	6.07E-08	2.30E-06
ZNF703	0	15.5743	Inf	6.07E-05	0.000757821
ZNF718	0	14.1218	Inf	1.55E-06	3.62E-05
ZNF74	0	13.5506	Inf	3.64E-05	0.00050071
ZNF746	0	14.0662	Inf	4.70E-05	0.000613083
ZNF783	0	14.1407	Inf	3.96E-06	7.98E-05
ZNF793	0	9.20111	Inf	4.32E-06	8.63E-05
ZNF805	0	11.5789	Inf	1.55E-10	1.13E-08
ZNF827	0	7.02124	Inf	3.64E-05	0.00050071
ZNF831	1.03639	12.3762	3.577929511	4.96E-08	1.94E-06
ZNF853	0	14.2161	Inf	7.84E-05	0.000933973
ZNF862	0	16.188	Inf	3.97E-10	2.65E-08
ZNF865	0	15.8395	Inf	3.34E-07	9.75E-06
ZNRF3	0	8.15003	Inf	3.34E-05	0.000466589
ZSCAN2	2.21204	18.8821	3.093569846	1.87E-05	0.000290534
ZSCAN22	2.09913	21.0749	3.327662364	1.40E-06	3.34E-05
ZSWIM5	0	10.6325	Inf	8.54E-06	0.000151469
ZSWIM8	3.21499	16.7788	2.383753384	2.49E-05	0.000367051
ZXDB	0	10.1615	Inf	2.18E-05	0.000327135
ZXDC	0	9.99098	Inf	1.42E-05	0.000232345
